# Cuproptosis-related gene FDX1 as a prognostic biomarker for kidney renal clear cell carcinoma correlates with immune checkpoints and immune cell infiltration

**DOI:** 10.3389/fgene.2023.1071694

**Published:** 2023-01-23

**Authors:** Yimin Yao, Haixin Chen, Minjun Lou, Tingting Chen

**Affiliations:** ^1^ Medical Laboratory, The First Affiliated Hospital of Zhejiang Chinese Medical University (Zhejiang Provincial Hospital of Chinese Medicine), Hangzhou, China; ^2^ The First Clinical Medical College, Zhejiang Chinese Medical University, Hangzhou, China

**Keywords:** cuproptosis-related gene, fdx1, prognostic biomarker, kidney renal clear cell carcinoma, immune checkpoints, immune cell infiltration

## Abstract

**Background:** Kidney renal clear cell carcinoma (KIRC) is not sensitive to radiotherapy and chemotherapy, and only some KIRC patients can benefit from immunotherapy and targeted therapy. Cuproptosis is a new mechanism of cell death, which is closely related to tumor progression, prognosis and immunity. The identification of prognostic markers related to cuproptosis in KIRC may provide targets for treatment and improve the prognosis of KIRC patients.

**Methods:** Ten cuproptosis-related genes were analyzed for differential expression in KIRC-TCGA and a prognostic model was constructed. Nomogram diagnostic model was used to screen independent prognostic molecules. The screened molecules were verified in multiple datasets (GSE36895 and GSE53757), and in KIRC tumor tissues by RT-PCR and immunohistochemistry (IHC). Clinical correlation of cuproptosis-related independent prognostic molecules was analyzed. According to the molecular expression, the two groups were divided into high and low expression groups, and the differences of immune checkpoint and tumor infiltrating lymphocytes (TILs) between the two groups were compared by EPIC algorithm. The potential Immune checkpoint blocking (ICB) response of high and low expression groups was predicted by the “TIDE” algorithm.

**Results:** FDX1 and DLAT were protective factors, while CDKN2A was a risk factor. FDX1 was an independent prognostic molecule by Nomogram, and low expressed in tumor tissues compared with adjacent tissues (*p* < 0.05). FDX1 was positively correlated with CD274, HAVCR2, PDCD1LG2, and negatively correlated with CTLA4, LAG3, and PDCD1. The TIDE score of low-FDX1 group was higher than that of high-FDX1 group. The abundance of CD4^+^ T cells, CD8^+^ T cells and Endothelial cells in FDX1-low group was lower than that in FDX1-high group (*p* < 0.05).

**Conclusion:** FDX1, as a key cuproptosis-related gene, was also an independent prognostic molecule of KIRC. FDX1 might become an interesting biomarker and potential therapeutic target for KIRC.

## Introduction

Renal cell carcinoma (RCC) accounts for about 2% of adult malignant tumors, and kidney renal clear cell carcinoma (KIRC) is the most important pathological type (Hakimi et al., 2016). Advanced RCC patients are not sensitive to radiotherapy and chemotherapy, and about 30% of RCC patients have metastasized at the initial diagnosis (Jakubek et al., 2020). Immunotherapy brings a turning point for advanced RCC patients, which can improve the survival (Li et al., 2020). However, not all RCC patients are effective with existing immunotherapies. How to mine the new key prognostic molecules and develop the corresponding targeted drugs are expected to bring dawn to advanced RCC patients who are ineffective to the existing targeted drugs.

Copper ions, as an important cofactor of many enzymes in the body, participate in oxidative stress, lipid metabolism and energy metabolism (Meydan et al., 2017). It can combine with copper carrier to induce apoptosis (Xiong et al., 2020). The mechanism of cuproptosis is mainly through the combination of copper ions and liposylated protein in the tricarboxylic acid cycle (TCA), which reduces the level of iron sulfur cluster protein, causes cytotoxic stress, and finally leads to cell death (Tsvetkov et al., 2022). Copper ions maintain a steady state in the body. When cuproptosis-related genes mutation or expression change, it can lead to copper ions imbalance and induce a series of diseases (Bao et al., 2022; Wang et al., 2022). Cuproptosis, different from traditional programmed death, has become a hot spot in tumor treatment field in recent years. Some copper chelating agents for tumor treatment, such as tetrathiomolybdate (TM), are in clinical trials (Lopez et al., 2019; Li Y. et al., 2022). In breast cancer and malignant pleural mesothelioma, the copper chelating strategy successfully delayed the progression and metastasis of tumors (Zhou et al., 2019). When using platinum compounds, copper chelators can regulate copper homeostasis and reduce cisplatin resistance (Zhu et al., 2017). Therefore, we explored the key regulatory molecules by studying the cuproptosis-related genes in KIRC, which help to develop new treatment strategies and bring hope to advanced RCC patients. In addition, the unregulation of immune checkpoints can inhibit the anti-tumor function of immune cells, leading to tumor cells escape (Mayadev et al., 2020). Analyzing the relationship between cuproptosis-related genes and immune cell status also help us master the immune statues of KIRC patients, optimize treatment strategies constantly, and prolong the prognosis. Zilong Bian et al. found that cuproptosis-related genes were closely related to the Overall Survival (OS) and Progression-Free-Survival (PFS) in KIRC through the prognostic risk score, and were also related to the immune infiltration and PD-1 expression (Bian et al., 2022). Wangli Mei et al. found the expression of cuproptosis-related genes (ATP7B, DBT, DLAT, LIAS and PDHB) were significantly different between tumor tissues and adjacent tissues, and speculated that these genes might be independent prognostic factors of OS in KIRC (Mei et al., 2022). However, their research was not verified by clinical samples, nor had they excavated the core gene of cuproptosis-related genes in KIRC.

In our study, we analyzed the expression changes of cuproptosis-related genes in KIRC-TCGA database, and constructed the relevant signature. FDX1 was screened as a key cuproptosis-related gene through COX regression analysis and closely related to the prognosis of KIRC. The differential expression of FDX1 was verified in clinical tumor tissues by RT-qPCR and immunohistochemistry (IHC). The correlation of FDX1 with immune cell infiltration and immune checkpoints were also analyzed. Finally, we concluded that FDX1 was the key cuproptosis-related gene of KIRC and FDX1 may become a new therapeutic target to improve the prognosis of KIRC patients.

## Materials and methods

### Data sources

The KIRC-TCGA database (3 May 2022) including clinial information and processed RNA-sequencing expression (level 3) data were downloaded from The Cancer Genome Atlas (TCGA) (https://portal.gdc.com). Seventy-two paracancerous samples and 532 tumor samples were collected to analyze the expression differences and clinical correlation.

GSE36895 (Peña-Llopis et al., 2012) and GSE53757 (von Roemeling et al., 2014) datasets from the Gene Expression Omnibus (GEO) database were downloaded to verify the screened key genes. GSE36895 dataset included 76 samples, of which 52 samples were collected and divided into KIRC group (29 primary tumors samples) and control group (23 normal kidney cortices samples). GSE53757 dataset included 144 samples, of which 72 tumor tissues were defined as KIRC group, and the other 72 normal kidney tissues were defined as control group.

Ten KIRC tissues and six paracancerous tissues from KIRC patients who underwent tumor resection in Zhejiang Province Hospital of Traditional Chinese Medicine were collected for validation. Histological diagnosis and tumor grade were assessed by three experienced pathologists following WHO/ISUP grading of renal clear cell carcinoma. All procedures were approved by the Ethics Committee of our Hospital.

### Cuproptosis-related genes were used to constructed prognostic model

Univariate Cox analysis on 532 RNA-seq data from TCGA-KIRC dataset was performed by “survival” R package to gain potential prognostic genes (*p* < .05, coding genes, and the prognosis type was overall survival (OS)). Then, these potential prognostic genes intersected with 10 cuproptosis-related genes (FDX1、LIAS、LIPT1、DLD、DLAT、PDHA1、PDHB、MTF1、GLS and CDKN2A) (Tsvetkov et al., 2022) to obtain cuproptosis-related potential prognostic genes using “ggplot2” R package. LASSO regression analysis was used to reduce the dimension and construct a prognostic model (signature). Potential prognostic genes related to cuproptosis were identified by “glmnet” R package and “survival” R package, and 10 folds cross validation was used.

The “ggplot2” R package was used to evaluate the risk score and grouping of the prognosis model for RNA-seq data and clinical information in TCGA-KIRC dataset. Use “ggplot2” R package and “timeROC” R package to evaluate the area under the characteristic curve and evaluate the predictive ability of the prognosis model. The survival of KIRC patients in high-risk group and low-risk group was analyzed with “survminer” R package and “survival” R package.

### Screening of independent prognostic molecules related to cuproptosis (nomogram prognostic model)

Univariate and multivariate cox regression analysis were performed to identify the proper terms to build the Nomogram. The forest was used to show the *p*-value, HR and 95% CI of each variable through “forestplot” R package. Univariate analysis showed differences, which were considered to be related to prognosis, while multivariate analysis also showed differences, which were considered to be independent prognostic factors. A Nomogram was developed based on multivariate cox proportional hazards analysis to predict the 1, 3, 5-year overall recurrence.

The Nomogram provided a graphical representation of the factors which can be used to calculate the risk of recurrence for an individual patient by the points associated with each risk factor through “rms” R package.

### Differential expression of cuproptosis-related independent prognostic molecules

The differential expression of cuproptosis-related independent prognostic molecules in TCGA-KIRC was analyzed through “ggplot2” R package. Gene expression profile for cuproptosis-related independent prognostic molecules were downloaded from GSE36895 and GSE53757, boxplots were constructed using “ggplot2” R package to compare the differences between different groups (Gene expression levels of different groups samples were compared using Wilcoxon rank sum test. Significance identification: NS, *p* ≥ .05; *, *p* < .05; **, *p* < .01; ***, *p* < .001.

### Clinical tissue validation by RT-qPCR and IHC

RNA was extracted from 3 to 5 10 μM thick scrolls obtained from formalin-fixed paraffin embedded (FFPE) tissues using the Spacegen nucleic acid extraction kit (Batch number: HS221121701). CT value was obtained by RT-qPCR, the sequence of the forward primer for FDX1 is TTC​AAC​CTG​TCA​CCT​CAT​CTT​TG, the reverse primer sequence is TGC​CAG​ATC​GAG​CAT​GTC​ATT, and the relative quantitative analysis was carried out by “ggplot2” R package.

KIRC FFPE tissues and paracancerous FFPE tissues were sliced into 4–6 μm sections, and FDX1 florescence intensity was analyzed by IHC. After deparaffinization, rehydration and microwave antigen retrieval, the slides were incubated with ADX (BOSTER, Cat #M04441) antibody at 1:100 dilution at 4°C overnight. Then, the slides were incubated with secondary antibody at room temperature for 30 min and stained with DAB substrate, followed by haematoxylin counterstaining.

### Clinical correlation of cuproptosis-related independent prognostic molecules

The RNA-seq data and clinical data of 609 samples of TCGA-KIRC were analyzed by “ggplot2” R package to analyze the clinical correlation of independent prognostic molecules related to cuproptosis, including T stage, N stage, M stage, Age, Gender, Pathological stage, Historical grade and Laterality.

### Correlation between cuproptosis-related independent prognostic molecules and other cuproptosis-related genes

The selected molecule was an independent prognostic molecule related to cuproptosis. Input the other cuproptosis-related genes in the molecular list. Through the “ggplot2” R package, select the statistical method as Spearman, and perform single gene co-expression analysis on the RNA-seq data of 532 samples of TCGA-KIRC.

In order to further understand the correlation between 10 cuproptosis-related molecules, input 10 cuproptosis-related gene names in the molecular list, and select Spearman correlation analysis method to perform correlation analysis on RNA-seq data of 532 samples of TCGA KIRC through “ggplot2” R package.

### Immune checkpoint expression analysis and immune checkpoint blocking (ICB) response

According to the expression of cuproptosis-related independent prognostic key gene, the KIRC samples from KIRC-TCGA database were divided into high and low expression groups. The expression level above the median level was defined as high expression group, and the expression level below the median level was defined as low expression group. Referring to some high-quality literatures, the expression differences of immune checkpoint related genes (SIGLEC15, TIGIT, CD274, HAVCR2, PDCD1, CTLA4, LAG3 and PDCD1LG2) were compared between these two groups by “ggplots2” and “pheatmap” R packages (Ravi et al., 2018; Wang et al., 2019; Zeng et al., 2019; Yi et al., 2020). Spearman’s correlation analysis was used to describe the correlation between quantitative variables without normal distribution, and *p*-value less than 0.05 was considered statistically significance.

The overall survival rate was selected as the prognosis type, and Cox regression was selected as the statistical method. The “survminer” package was used for visualization and the “survival” package was used for statistics. The “TIDE” algorithm predicted the potential ICB response of high and low expression groups, “ggplot2” and “ggpubr” packages were used for mapping analysis.

### Analysis of immune infiltrate

The KIRC samples from KIRC-TCGA were stratified into two groups based on the expression of cuproptosis-related independent prognostic molecule. Immune score evaluation was performed by “immunedeconv” R package using EPIC, data were visualized using “ggplot2” and “pheatmap” R packages. Further survival analysis of immune infiltrating cells was carried out using TIMER2.0 (http://timer.cistrome.org/), a web server designed for comprehensive analysis of tumor-infiltrating immune cells.

EPIC algorithm was used to evaluate the immune infiltration of GSE53575 dataset to further verify the correlation between cuproptosis-related independent prognostic molecule and KIRC immune infiltration. The expression values of EPIC algorithm of immune infiltrating cells B cell, CD4^+^ T cell, CD8^+^ T cell, Endothelial cell, Macrophage and NK cell were extracted, and the expression of immune infiltrating cells were analyzed visually through “ggplot2” and “pheatmap” R packages.

### R software version

All the analysis methods and R packages were implemented by R (foundation for statistical computing 2020) version 4.0.3. *p* < .05 was considered statistically significant.

## Results

### FDX1, DLA and CDKN2A cuproptosis-related genes prognostic model (signature)

**FIGURE 1:**
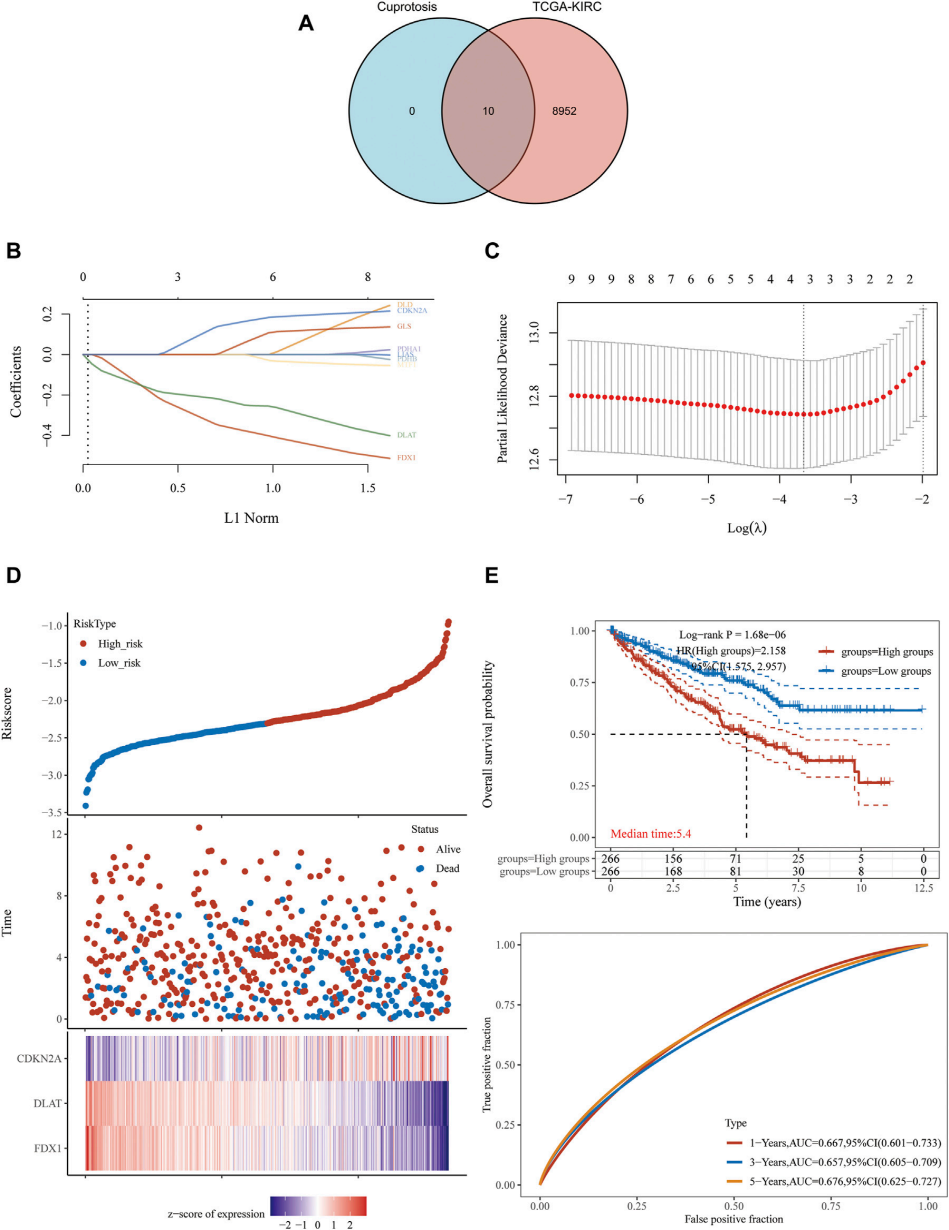
FDX1, DLAT and CDKN2A cuproptosis-related genes prognostic model. **(A)** 8,962 potential prognostic molecules of KIRC and 10 cuproptosis-related genes Venn diagram. **(B)** LASSO variable trajectory diagram. **(C)** LASSO coefficient screening diagram. **(D)** The prognostic risk factor graph, red represents high-risk group, blue represents low-risk group. **(E)** Kaplan-Meier survival curve and time dependent ROC.

In order to explore whether cuproptosis-related genes can be used as effective biomarkers to indicate the prognosis of KIRC, we obtained 8,962 potential prognostic molecules of KIRC from KIRC-TCGA database. Then, these 8,962 prognostic molecules were intersected with 10 cuproptosis-related genes (FDX1, LIAS, LIPT1, DLD, DLAT, PDHA1, PDHB, MTF1, GLS and CDKN2A), we found these 10 genes were all in these intersection (Figure 1A).

LASSO regression analysis was used to fit the overall survival of KIRC patients according to the selected 10 potential prognostic genes related to cuproptosis. Key genes will be assigned to a non-zero coefficient and selected to build a prognostic model (signature). Last, we built a prognostic scoring formula: Riskscore = (−0.3336) *FDX1 + (−0.215) *DLAT + (0.1233) *CDKN2A. Three genes in the model were multiplied by a weight. Negative numbers represent protective genes while positive numbers represent risk genes. We concluded that FDX1 and DLAT were protective factors, while CDKN2A was a risk factor. LASSO variable trajectory diagram showed that the selection method L1 norm, at the position of 3, the vertical line cut to the variables with the corresponding coefficient of non-0 were FDX1, DLAT and CDKN2A (Figure 1B).

LASSO coefficient screening diagram indicated that when lambda. min was 3, the partial likelihood deviance was the smallest, and the corresponding model was the better (Figure 1C).

The prognostic risk factor graph showed the Riskscore, survival time and survival status in KIRC-TCGA. The top graph represented the scatter plot of the Riskscore from low to high and the middle figure represented the scatter diagram distribution of survival time and survival state corresponding to Riskscore of different samples. The corresponding death patients in the high-risk area were higher than those in the low-risk area. The bottom figure represented the expression heat map of three molecules including FDX1, DLAT and CDKN2A in the signature.

FDX1 and DLAT were low expression and CDKN2A was high expression under the corresponding high-risk region, FDX1 and DLAT were high expression and CDKN2A was low expression under the corresponding low-risk region (Figure 1D). In the prognostic model, the survival probability of high-risk group and low-risk group was significantly different, and the prognosis of high-risk group was poor through log rank test according to the Kaplan-Meier survival curve [*p* = 1.68e−06, HR = 2.158, 95%CL (1.575, 2.957)]. The AUC of this prognostic model at 1, 3 and 5 years were .667, .657 and .676, respectively. It indicated that cuproptosis-related genes (FDX1, DLAT and CDKN2A) as predictive prognostic models had good accuracy (Figure 1E).

### FDX1 is an independent prognostic molecule associated with cuproptosis by Nomogram

**FIGURE 2:**
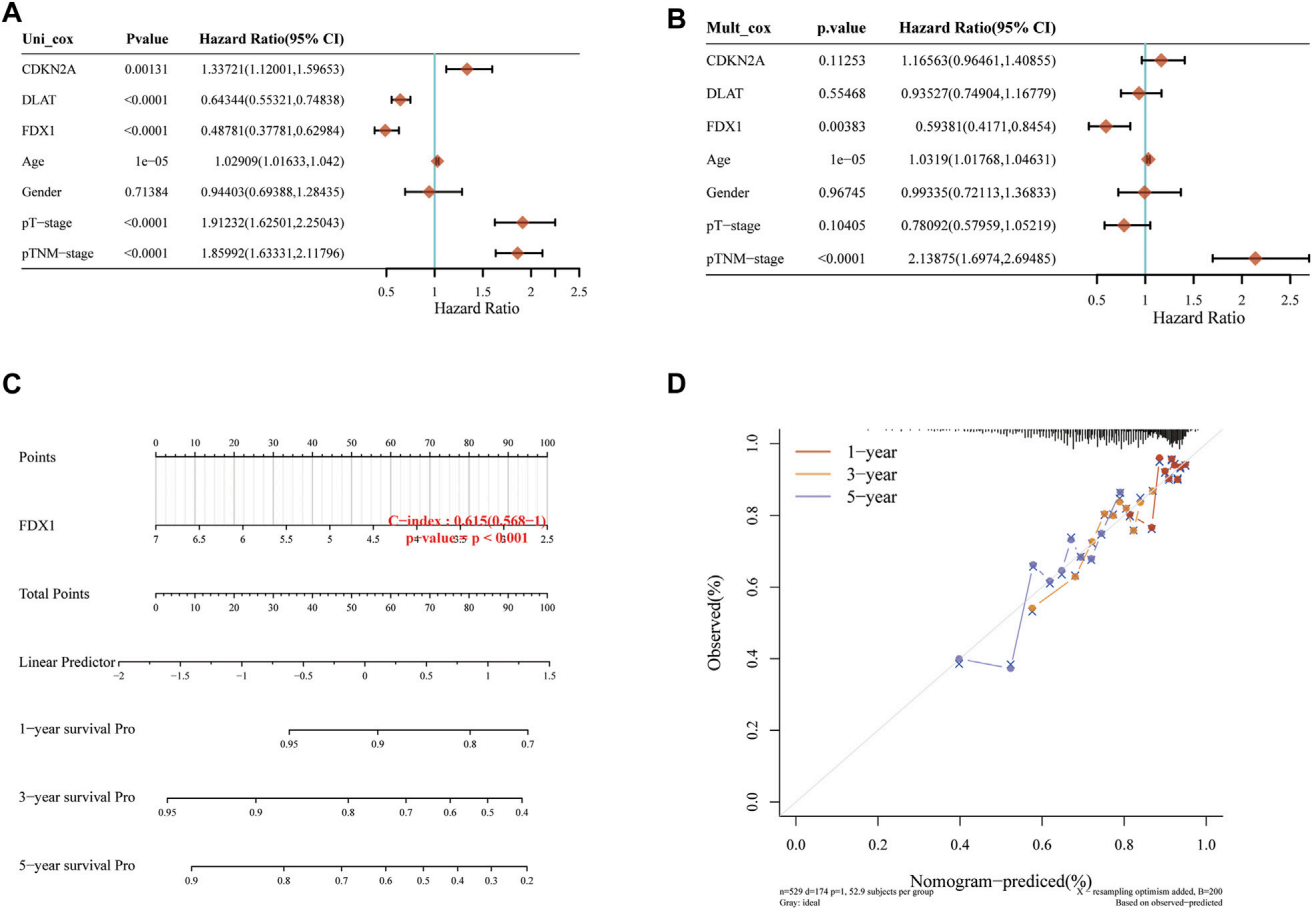
Independent prognostic molecules associated with cuproptosis. **(A)** Univariate Cox analysis of cuproptosis-related genes. **(B)** Multivariate Cox analysis of cuproptosis-related genes. **(C)** one, two and three-year overall survival of KIRC patients were predicted by Nomogram. **(D)** Calibration curve of the overall survival Nomogram model in the discovery group.

Univariate and multivariate Cox regression can identify variables in Nomogram. The prognosis of FDX1 showed significant differences in univariate and multivariate, which indicated that FDX1 was a variable independent of other clinical factors (Figures 2A, B). The total points and 1, 3, 5-year survivals were inversely proportional to the expression of FDX1 (Figures 2C, D). The closer the Nomogram model was to the calibration curve, the better the predicted result of the model.

### The expression difference of FDX1 in KIRC

**FIGURE 3:**
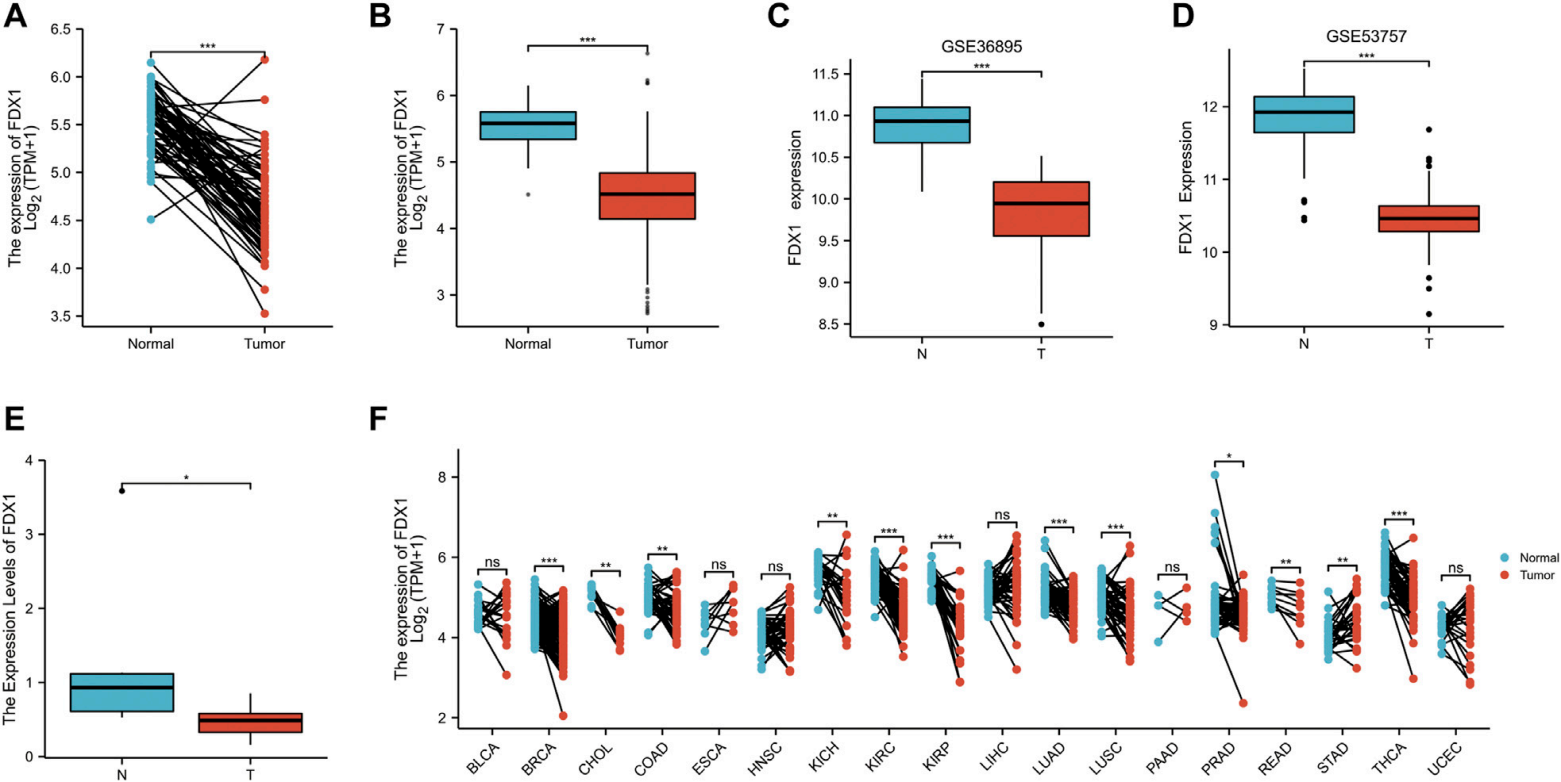
The expression difference of FDX1 in KIRC. **(A)** Differential expression of FDX1 in paired samples in TCGA-KIRC database. **(B)** Differential expression of FDX1 in unpaired samples in TCGA-KIRC database. **(C)** Differential expression of FDX1 in GSE36895. **(D)** Differential expression of FDX1 in GSE53757. **(E)** Differential expression of FDX1 between KIRC patients and normal renal tissue by RT-qPCR. **(F)** Differential expression of FDX1 in TCGA pan cancer. NS, *p* > .05; *, *p* < .05; **, *p* < .01; ***, *p* < .001.

According to Nomogram model, we concluded that FDX1 was an independent prognostic factor of KIRC related to prognosis and survival. In KIRC-TCGA database, the expression of FDX1 was decreased in tumor tissues compared with adjacent tissues (*p* < .05) (Figures 3A, B). In GSE36895 and GSE53757, the expression of FDX1 was also verified to be lower in tumor tissues than in normal renal tissues (*p* < .05) (Figures 3C, D). Consistent with the bioinformatics analysis, our clinical samples showed that FDX1 expression was lower in renal tumor tissues than in adjacent tissues (*p* < .05) (Figure 3E).

The FDX1 expression was further analyzed in Pan cancer in TCGA database, and the FDX1 expression was lower in tumor tissues than in normal tissues in breast cancer (BRCA), Cholangio carcinoma (CHOL), Colon adenocarcinoma (COAD), Kidney Chromophobe (KICH), Kidney renal clear cell carcinoma (KIRC), Kidney renal papillary cell carcinoma (KIRP), Lung Adenocarcinoma (LUAD), Lung Squamous cell carcinoma (LUSC), Prostatic cancer (PRAD), Rectum adenocarcinoma (READ) and Thyroid carcinoma (THCA) (*p* < 0.05). However, FDX1 expression in tumor tissues of Stomach adenocarcinoma (STAD) was higher than in normal tissues (*p* < .05) (Figure 3F).

### FDX1 fluorescence intensity in KIRC tissues by IHC

**FIGURE 4:**
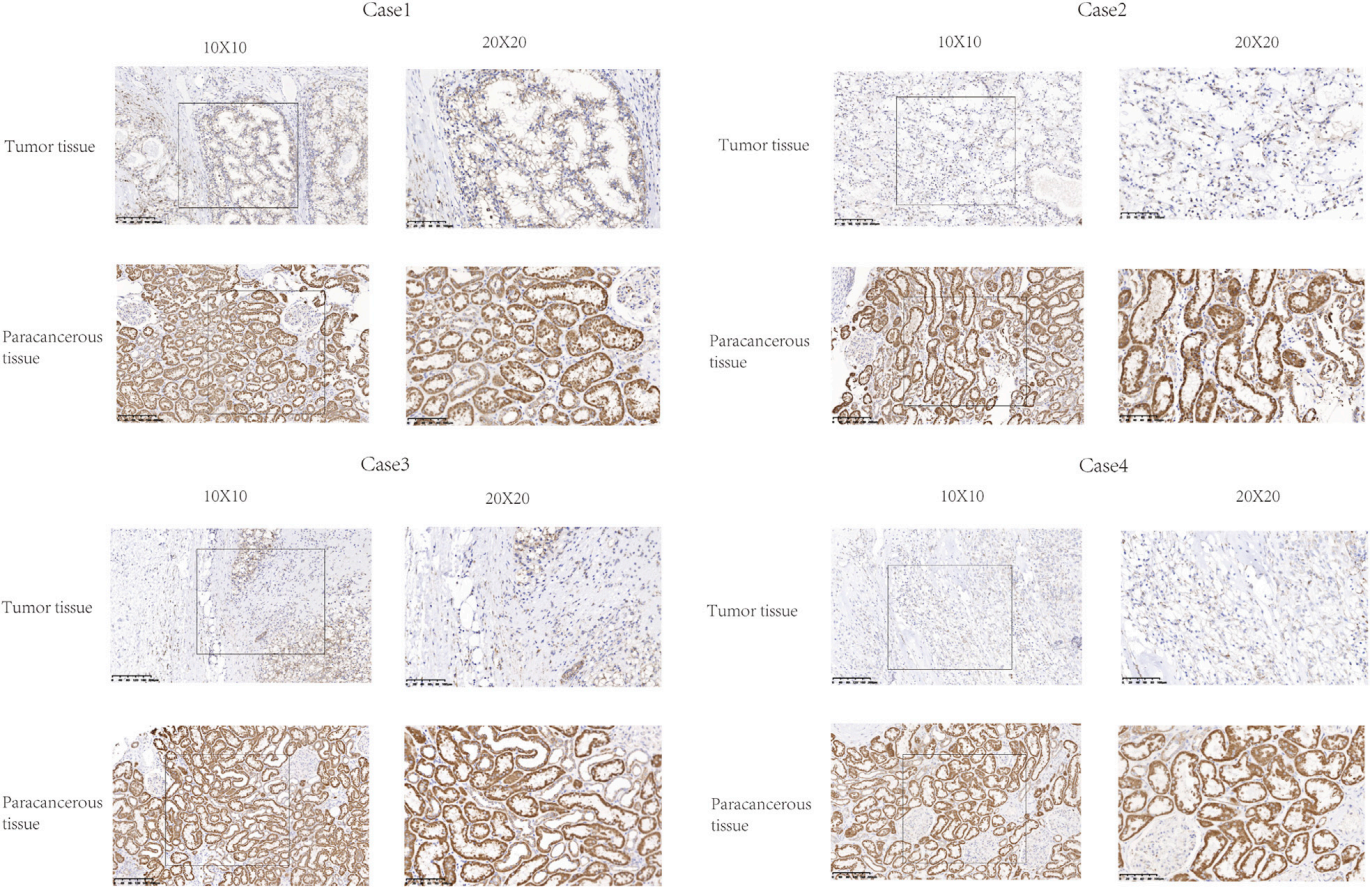
Representative images of FDX1 expression in KIRC tissues and their matched paracancerous tissues. Original magnifications ×100 and 400× (inset panels).

The expression of FDX1 was analyzed by IHC in six KIRC tissues and paired paracancerous tissues. The expression of FDX1 was downregulated in these six tumor tissues compared with the adjacent tissues (Figure 4).

### The correlation between FDX1 and clinical characteristics in KIRC

**FIGURE 5:**
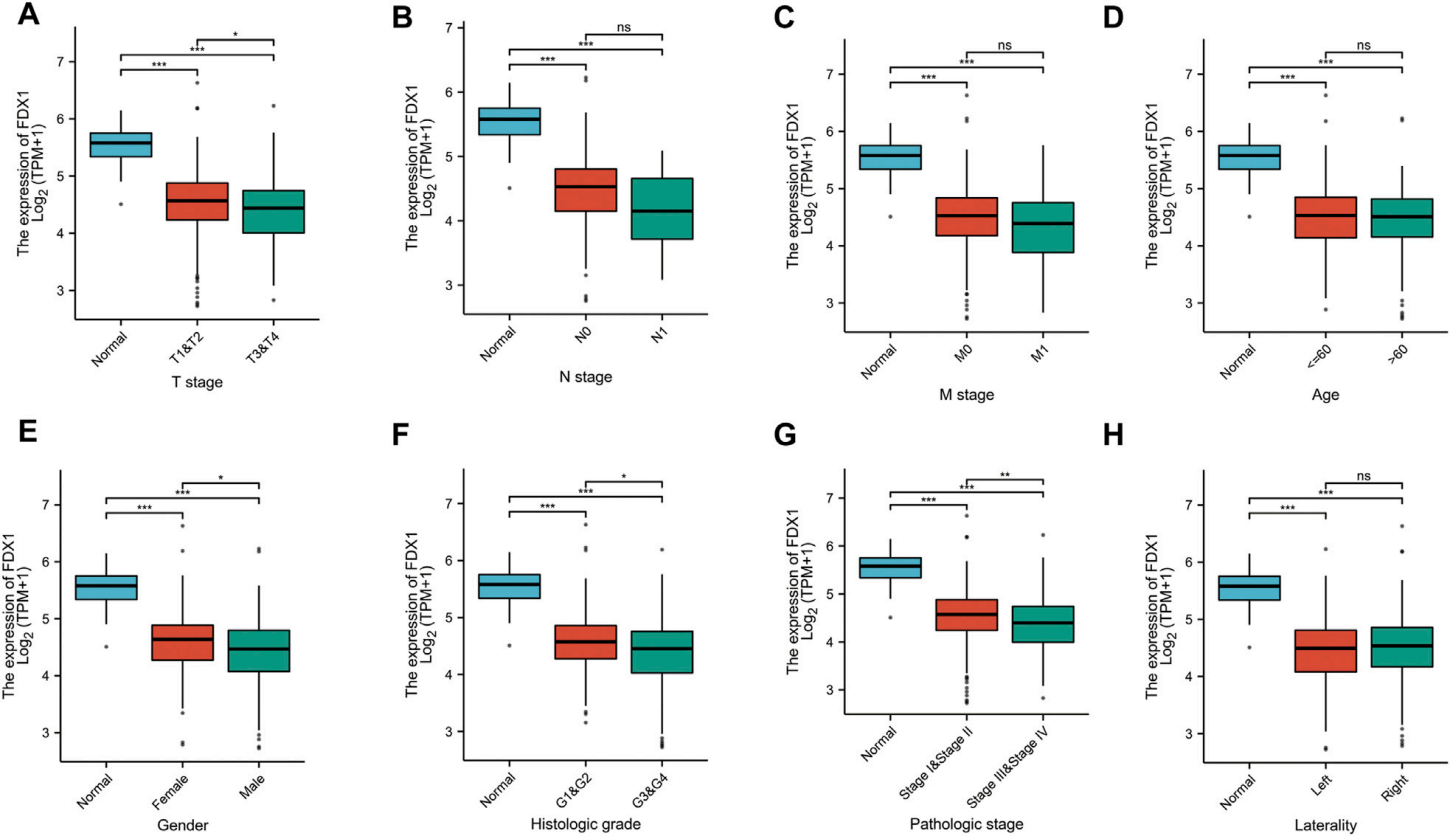
The correlation between FDX1 and clinical characteristics in KIRC. **(A)** The correlation between FDX1 and T stage. **(B)** The correlation between FDX1 and N stage. **(C)** The correlation between FDX1 and M stage. **(D)** The correlation between FDX1 and Age. **(E)** The correlation between FDX1 and Gender. **(F)** The correlation between FDX1 and Histologic grade. **(G)** The correlation between FDX1 and Pathologic stage. **(H)** The correlation between FDX1 and Laterality. NS, *p* > .05; *, *p* < .05; **, *p* < .01; ***, *p* < .001.

To explore the value of FDX1 in KIRC, we analyzed the correlation between the FDX1 expression and the clinical characteristics of KIRC (T stage, N stage, M stage, Age, Gender, Histologic grade, Pathologic stage, Laterality). T3&T4 was lower than T1&T2, Stage III&Stage IV was lower than Stage I&Stage II, G3&G4 was lower than G1&G2, male was lower than female (*p* < .05). But the years, N stage, M stage, left or right had no statistically significant (*p* > .05) (Figure 5).

### Correlation analysis between FDX1 and other cuproptosis-related genes

**FIGURE 6:**
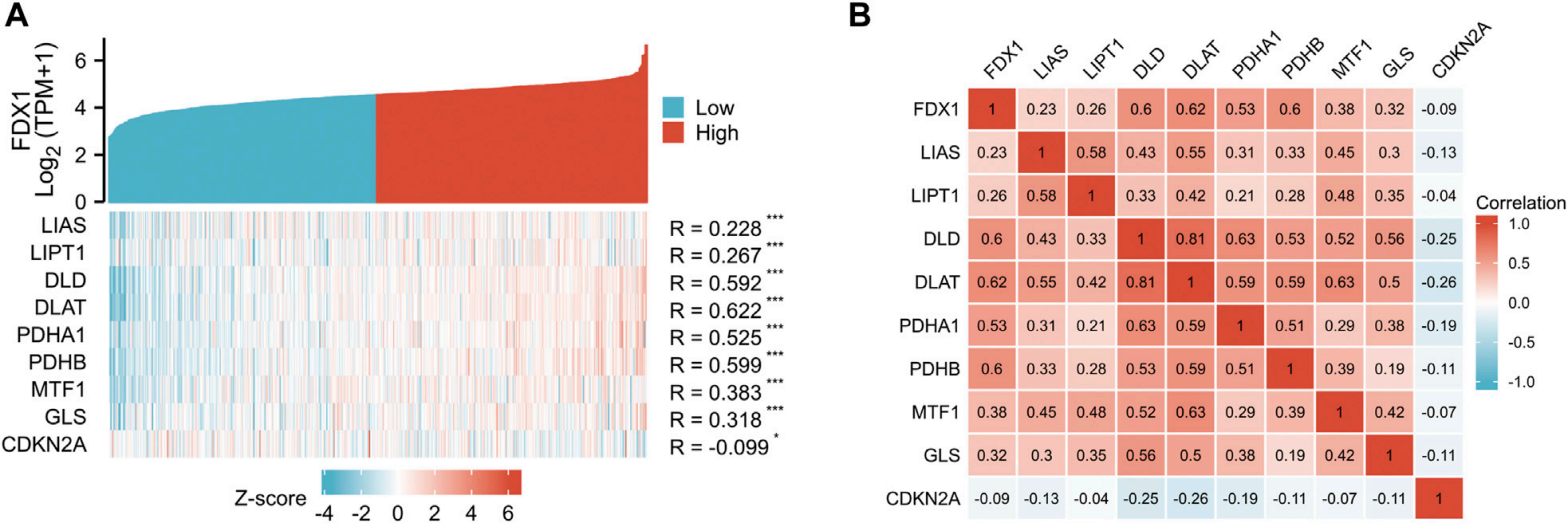
Correlation between FDX1 and other nine cuproptosis genes. **(A)** FDX1 and other nine cuproptosis-related molecules co-expression heatmap. **(B)** 10 cuproptosis-related genes correlation heatmap.

The coexpression Heatmap of single gene showed that FDX1 expression was correlated with the other nine cuproptosis-related genes in KIRC, and was strongly and positively correctly with DLD, DLAT, PDHA1 and PDHB (Figure 6A). Correlation Heatmap analysis showed that FDX1, DLD, DLAT, PDHA1 and PDHB were positively correlated and the correlation coefficient was greater than .5 (Figure 6B).

### Expression of immune checkpoint and prognosis in high and low expression groups of FDX1

**FIGURE 7:**
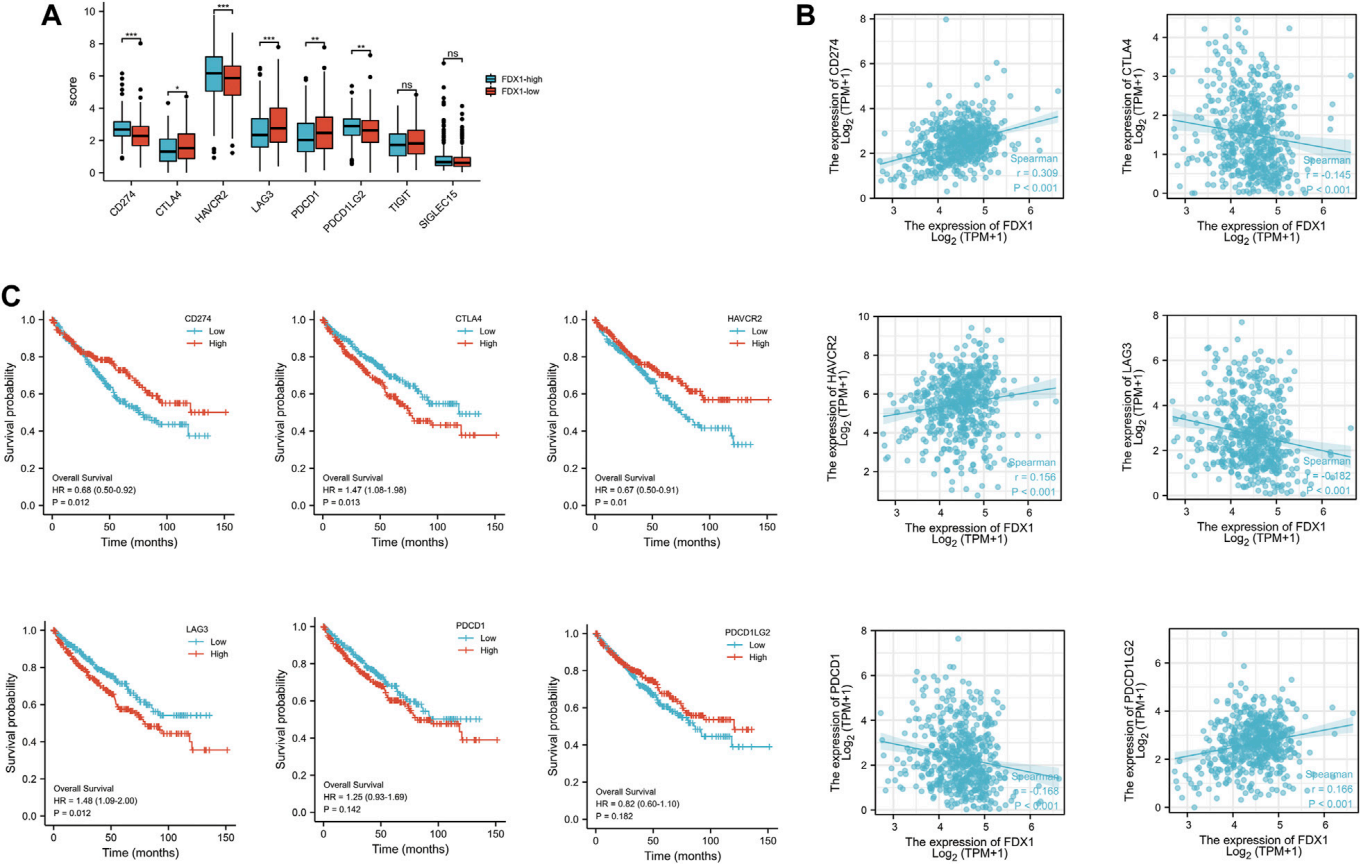
The expression and the prognosis analysis of immune checkpoints. **(A)** Expression of immune checkpoint in high and low expression groups of FDX1. **(B)** Scatter plot of immune checkpoint association with FDX1. **(C)** Overall survival curve of immune checkpoints in KIRC patients.

KIRC-TCGA database was divided into high expression group and low expression group according to FDX1. Eight immune checkpoints (SIGLEC15, TIGIT, CD274, HAVCR2, PDCD1, CTLA4, LAG3 and PDCD1LG2) were selected to be analyzed between high and low groups. The expression of CD274, HAVCR2 and PDCD1LG2 in FDX1-low group was lower than that in FDX1-high group (*p* < .05). However, the expression of CTLA4, LAG3 and PDCD1 in FDX1-low group was higher than that in FDX1-high group (*p* < .05) (Figure 7A).

The correlation analysis between FDX1 and immune checkpoint showed that FDX1 was positively correlated with CD274, HAVCR2, PDCD1LG2, and negatively correlated with CTLA4, LAG3, and PDCD1 (Figure 7B). Kaplan-Meier survival curve analysis illustrated that the overall survival (OS) was poor when CTLA1 and LAG3 expression was high, while CD274 and HAVCR2 expression was low (Figure 7C).

### Evaluation of immune blocking therapy and immune infiltration

**FIGURE 8:**
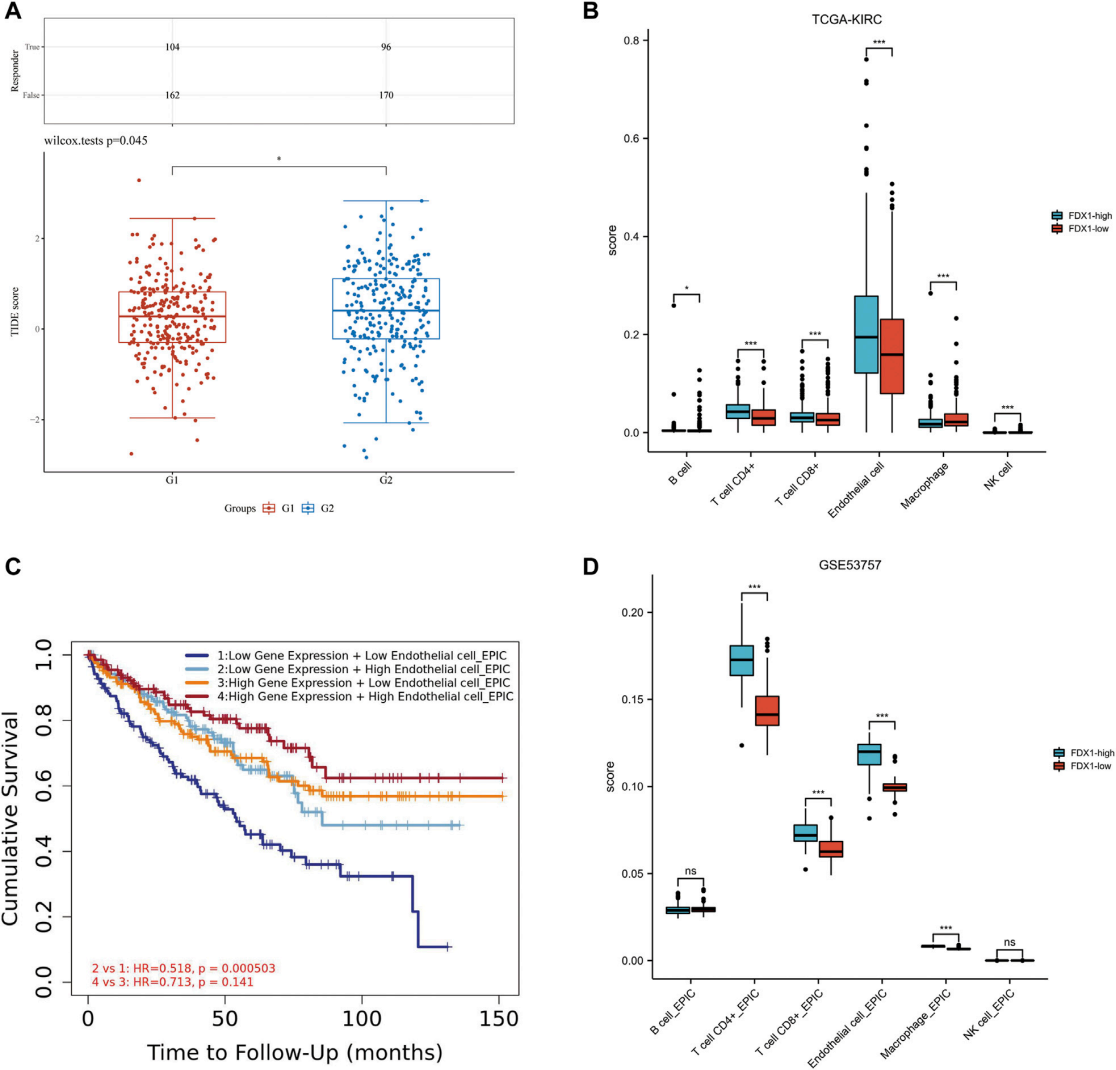
Relationship among FDX1 expression, ICB and immune cell infiltration in KIRC. **(A)** Distribution of immune response scores in high and low expression groups of FDX1. **(B)** The abundance of immune cell infiltration in FDX1-low group and FDX1-high group in TCGA-KIRC. **(C)** The Kaplan-Meier curve of Endothelial cells in KIRC. G1 group represented FDX1-high group, G2 group represented FDX1-low group. **(D)** The abundance of immune cell infiltration in FDX1-low group and FDX1-high group in GSE53757.

TIDE algorithm was used to predict the potential immunotherapeutic response. TIDE used a set of genes to evaluate two different mechanisms of tumor immune escape, including the dysfunction of tumor infiltrating cytotoxic T lymphocytes (CTLs) and the rejection of CTLs by immunosuppressive factors. High TIDE score predicted poor efficacy of immune checkpoint block therapy and short survival after ICB. The TIDE score of low-FDX1 group was higher than that of high-FDX1 group, indicating that the efficacy of ICB in low-FDX1 group was poor (Figure 8A).

The correlation between FDX1 and immune infiltration was further analyzed. In KIRC-TCGA database and GSE53757 dataset, the abundance of CD4^+^ T cells, CD8^+^ T cells and Endothelial cells in FDX1-low group was lower than that in FDX1-high group (*p* < 0.05) (Figures 8B, C). The Kaplan-Meier curve of Endothelial cells in KIRC showed that FDX1 was low expression and low Endothelial cell infiltration, and the cumulative survival was poor (Figure 8D).

## Discussion

RCC is one of the top ten cancers with high mortality (Clark et al., 2019). As the main pathological type of RCC, KIRC accounts for more than 80% of RCC (Alsaab et al., 2018). Advanced patients with KIRC are not sensitive to radiotherapy and chemotherapy, and miss the opportunity of surgery because of metastasis (Mu et al., 2020). Targeted therapy can benefit some patients, however, many advanced patients are ineffective to the existing targeted drug (Li et al., 2018). Because KIRC lacks precise targets and therapeutic mechanisms, it is necessary to identify the key molecules that affect the prognosis of KIRC. Targeting these molecules might become a new therapeutic target for KIRC and bring hope to patients with advanced KIRC.

Copper is an essential cofactor for organisms, and excess copper induces cell death if its concentration exceeds the steady-state threshold (Tsvetkov et al., 2022). Cuproptosis is different from the known death mechanism and mainly depends on mitochondrial respiration (Ren et al., 2019). It occurs by direct binding of copper to the fatty acylated components of the TCA cycle, which leads to the aggregation of lipo-acylated proteins, the subsequent loss of iron sulfur cluster proteins, proteotoxic stress and ultimately to cell death (Li S. R. et al., 2022). In some cancer, we found that the content of copper ions in tumor tissues and serum of tumor patients was higher than that of normal people (da Silva et al., 2022). Therefore, we analyzed whether there was differential expression of cuproptosis-related genes in KIRC. In our study, ten cuproptosis-related genes were differentially expressed in tumor tissues of KIRC compared with adjacent tissues (*p* < .05). Through our established prognostic model, we concluded that two protective genes (FDX1 and DLAT) and one pathogenic gene (CDKN2A) were closely related to prognosis. Through univariate and multivariate Cox regression analysis, we concluded that FDX1 was an independent prognostic gene of KIRC. However, the HR of pT-stage in univariate and multivariate Cox regression analysis were inconsistent. We speculated that because pTNM-stage caused a collinearity interference to pT-stage, it was not true that pT-stage was a risk factor of KIRC in univariate Cox regression analysis. FDX1, as a protein coding gene, has been reported to be involved in not only iron death but also cuproptosis (Zhang et al., 2022). FDX1 can regulate glucose, lipid and amino acid metabolism, affect the prognosis of LUAD and may participate in the occurrence and development of polycystic ovary syndrome (PCOS) (Wang et al., 2021; Zhang et al., 2021). Recently, Harvard team found that FDX1 is the key cuproptosis-related gene which encodes Elesclopmol (Tsvetkov et al., 2022). By analyzing the correlation between FDX1 and other cuproptosis-related genes in KIRC, we also found that FDX1 was positively correlated with DLD, DLAT, PDHA1 and PDHB, indicating that FDX1 was a key cuproptosis-related gene. Through bioinformatics and clinical tissues verification, we found that FDX1 was low expressed in tumor tissues of KIRC. In Zhang C’s study, they also found that FDX1 was low expressed in tumor tissues of KIRC, and speculated that FDX1might play a role in KIRC as a tumor suppressor gene (Zhang et al., 2022). Zilong Bian’s study showed that FDX1 was significantly related to the level of immune infiltration and the expression of programmed cell death protein 1 (PD-1) in KIRC, and they inferred that FDX1 could be used as a potential prognostic predictor for KIRC (Bian et al., 2022). Their researches were consistent with our conclusion, which verified the results of our data mining.

Many studies have shown that FDX1 was closely related to immune regulation in Pan-cancer (Liu, 2022; Zhang et al., 2022). We found that FDX1 was low expressed in KIRC tumor tissues and may be an independent prognostic gene of KIRC. Next, we analyzed the relationship between FDX1 and other eight immune checkpoints (SIGLEC15, TIGIT, CD274, HAVCR2, PDCD1, CTLA4, LAG3 and PDCD1LG2). We concluded that the expressions of CD274, HAVCR2 and PDCD1LG2 were positively correlated with FDX1 in KIRC, while CTLA4, LAG3 and PDCD1 were negatively correlated with FDX1 (*p* < .05). Kaplan-Meier survival curve analysis illustrated that the OS was poor when CTLA1 and LAG3 expression was high, while CD274 and HAVCR2 expression was low. Therefore, the FDX1 expression was positively related with CD274 and HAVCR2 expression, but negatively related with CTLA4 and LAG3 expression, indicating that the OS of KIRC patients was poor.

Immunotherapy can effectively inhibit the progression of KIRC, suggesting that immune cell infiltration may play an important role in the treatment of KIRC. Therefore, we analyzed the relationship between FDX1 and immune infiltration. The tumor infiltrating lymphocytes (TILs) also differentially expressed between low-FDX1 group and high-FDX1 group. Through the TIDE algorithm, the TIDE score in low-FDX1 group was higher than in high-FDX1 group, indicating that the lower the FDX1 expression, the worse the effect of ICB. Interestingly, the abundance of endothelial cells in low-FDX1 group was lower than that in high-FDX1 group (*p* < .05). According to the Kaplan-Meier curve of endothelial cells, the expression of FDX1 was low, the infiltration of endothelial cells was low, and the cumulative survival rate was low. Tumor metastasis depended to a large extent on the rapid and effective escape from the blood flow through the endothelial barrier (Morad et al., 2019). Tumor cell extravasation was similar to leukocyte migration through the endothelium. However, it is still unclear how tumor cells interact with endothelial cells during extravasation and how these processes are regulated. Studies have shown that tumor cells induce programmed necroptosis of endothelial cells, thereby promoting tumor cell extravasation and metastasis (Wettschureck et al., 2019). We inferred that FDX1 may participate in the interaction between tumor cells and endothelial cells, mediate the damage of endothelial cell barrier, and thus lead to tumor invasion and metastasis. Targeting FDX1 mediated cuproptosis of vascular endothelial cells to inhibit metastasis of KIRC may be a promising therapeutic approach.

Cuproptosis status affects treatment options about immunotherapy and targeted therapy for KIRC patients. Some studies revealed that FDX1 can regulate protein lipoylation modification, and high expression of lipoylated protein may become a new direction of tumor therapy (Zhang et al., 2021). According to our model, we found that FDX1 was not only an independent prognostic factor of KIRC, but also can regulate the status of immune cells and the expression of immune checkpoints in the tumor microenvironment (TME). Targeting FDX1 may become a new therapeutic target for KIRC.

However, this study still had some shortcomings. Firstly, we only verified the difference expression of FDX1 in tumor tissues and adjacent tissues of KIRC through clinical samples, and lacked further clinical experiments to verify immune infiltration and TILs. The number of samples we selected was not very large, nor does it cover different stages of KIRC. The insufficient number of samples made it impossible to further verify the prognostic value of FDX1 in KIRC. Secondly, we lack clinical relevance research and cannot analyze it with clinical information. Thirdly, although FDX1 was predicted as an independent prognostic factor of KIRC according to our Nomogram model, other important factors with predictive value were not considered in this study. In the next study, we will select more KIRC samples covering different stages to analyze and verify the regulatory mechanism of cuproptosis-related genes in KIRC, so as to lay a theoretical foundation for later development of targeted drugs.

In conclusion, our study showed that FDX1 was an independent factor affecting the prognosis of KIRC. The FDX1 expression in paired and unpaired tumor samples illustrated that the FDX1 downregulated compared with normal kidney tissues (*p* < .05). There was a significant correlation between FDX1 and immune infiltration in tumor samples of KIRC. The abundance of B cells, CD4 + T cells, CD8 + T cells, endothelial cells and NK cells in low-FDX1 group was lower than that in high-FDX1 group. Kaplan-Meier curve of endothelial cells showed low FDX1 expression and poor cumulative survival. Targeting FDX1 in KIRC may regulate cuproptosis to improve the prognosis of patients. Nevertheless, the specific pathogenesis and molecular targets still need to be further verified.

## Conclusion

FDX1, as a key cuproptosis-related gene, was also an independent prognostic molecule of KIRC. It was closely related to immune cell infiltration and immune checkpoint regulation. In KIRC, FDX1 expression was decreased, and targeted regulation of FDX1 expression may be helpful to improve patient prognosis.

## Data Availability

The original contributions presented in the study are included in the article/supplementary material, further inquiries can be directed to the corresponding author.
