# Supplementary figures and images for: Cuproptosis-related gene FDX1 as a prognostic biomarker for kidney renal clear cell carcinoma correlates with immune checkpoints and immune cell infiltration
Source: Front Genet. 2023 Jan 23;14:1071694. doi: 10.3389/fgene.2023.1071694 (PMC9900009; doi:10.3389/fgene.2023.1071694)

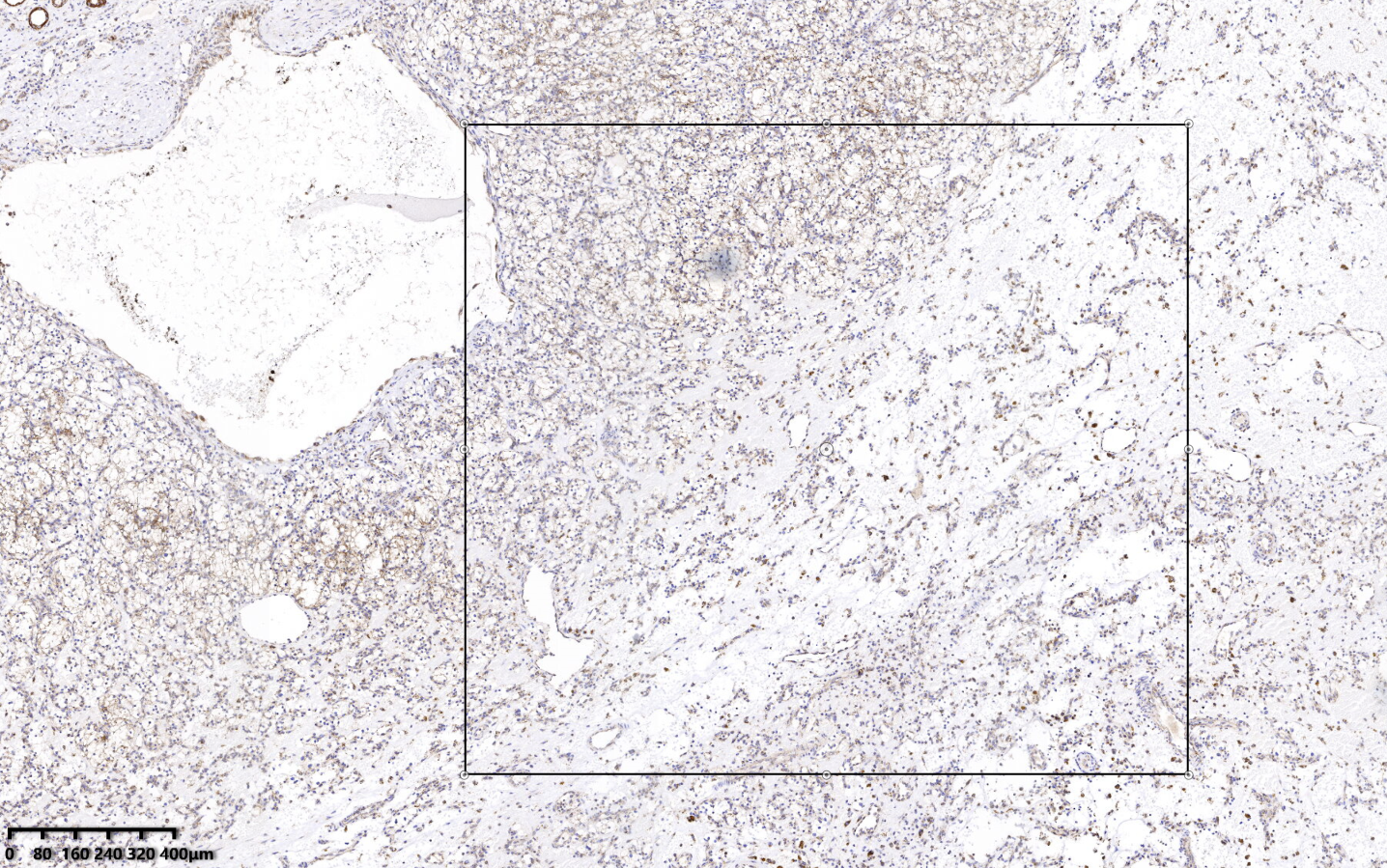

Supplement: Supplementary file 1 [file Presentation4.ZIP › Case2-T/20220819225218.tiff]

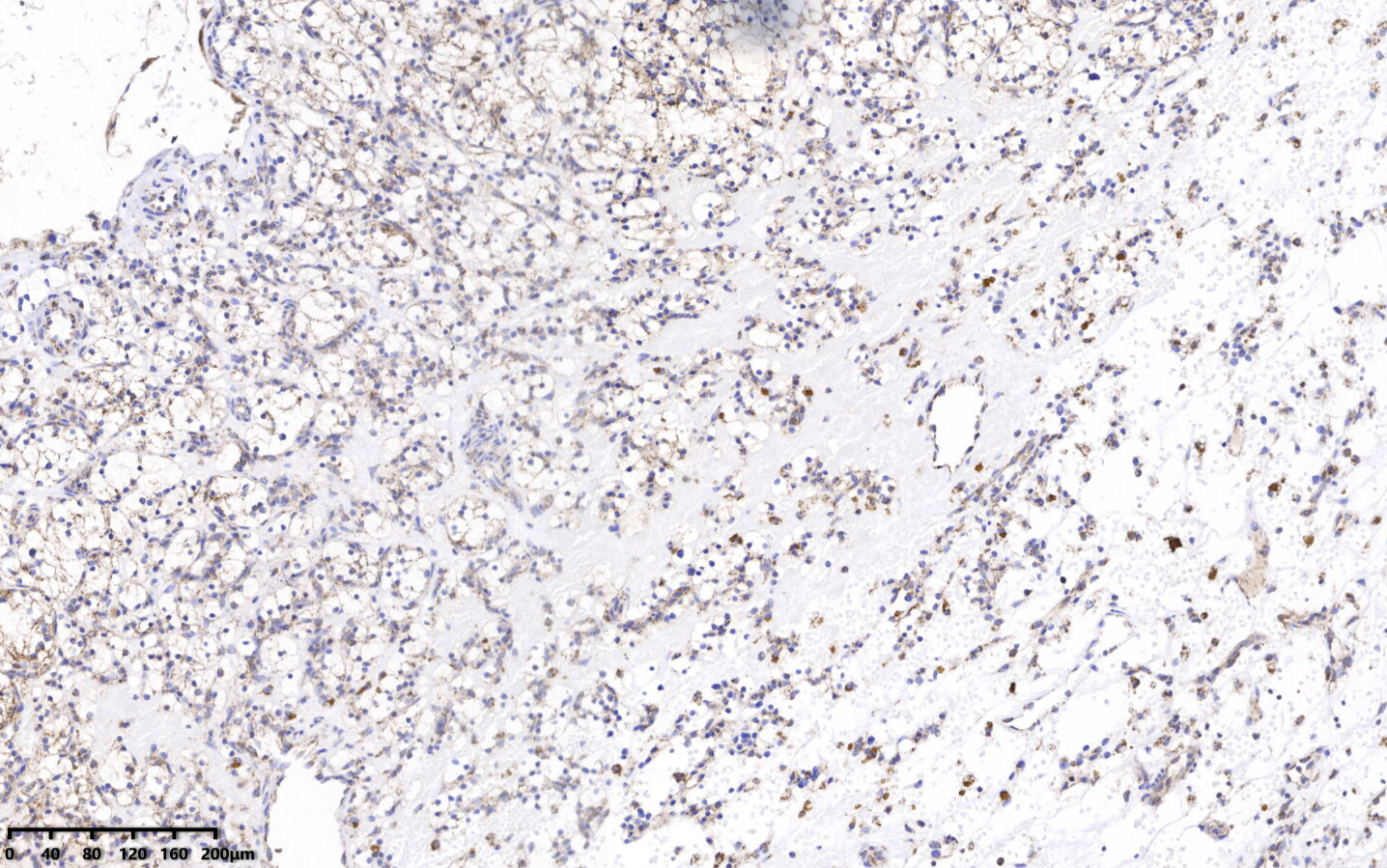

Supplement: Supplementary file 1 [file Presentation4.ZIP › Case2-T/20220819225312.tiff]

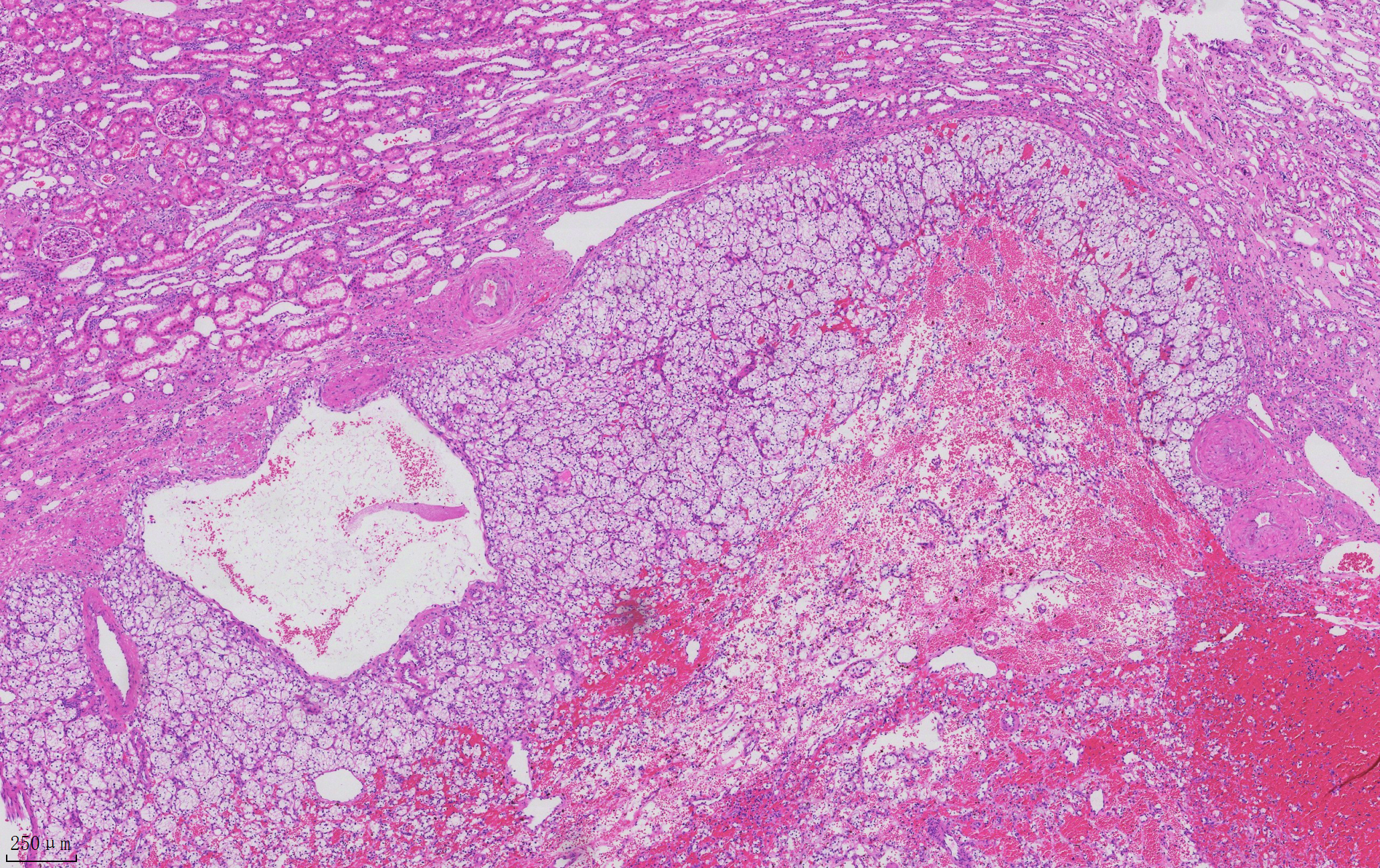

Supplement: Supplementary file 1 [file Presentation4.ZIP › Case2-T/20220819_224909.tiff]

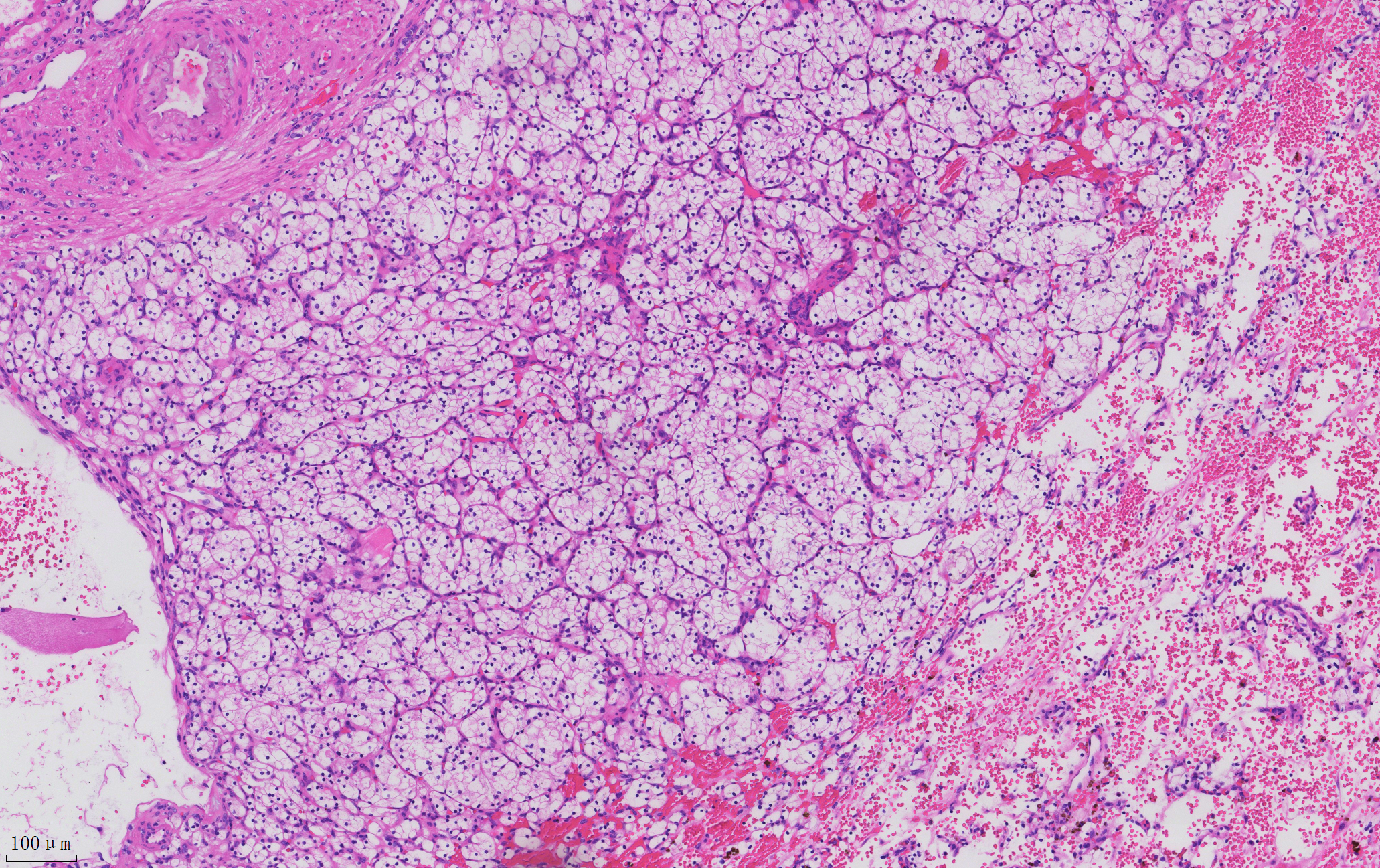

Supplement: Supplementary file 1 [file Presentation4.ZIP › Case2-T/20220819_224956.tiff]

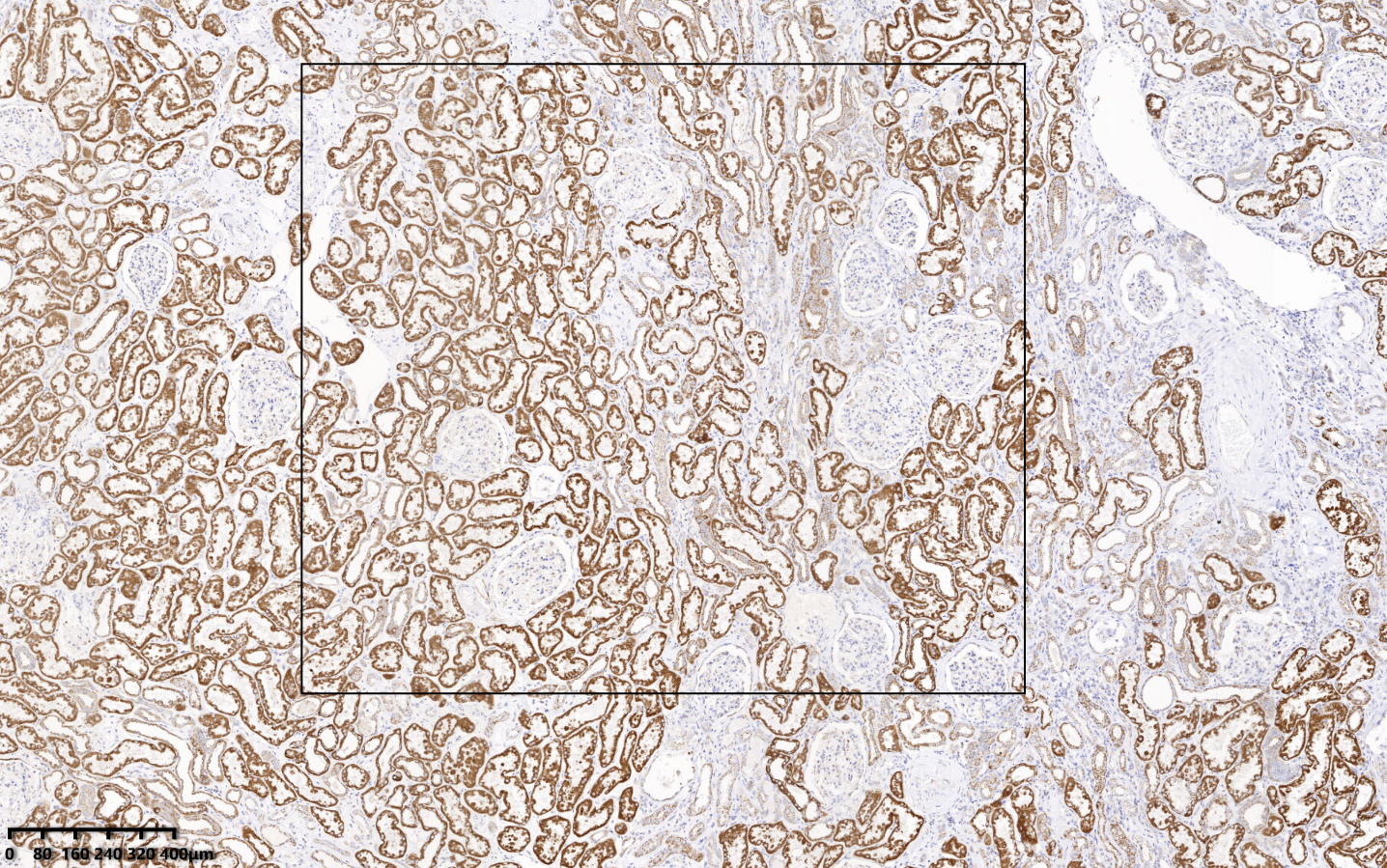

Supplement: Supplementary file 2 [file Presentation1.ZIP › Case1-P/20220820162628.tiff]

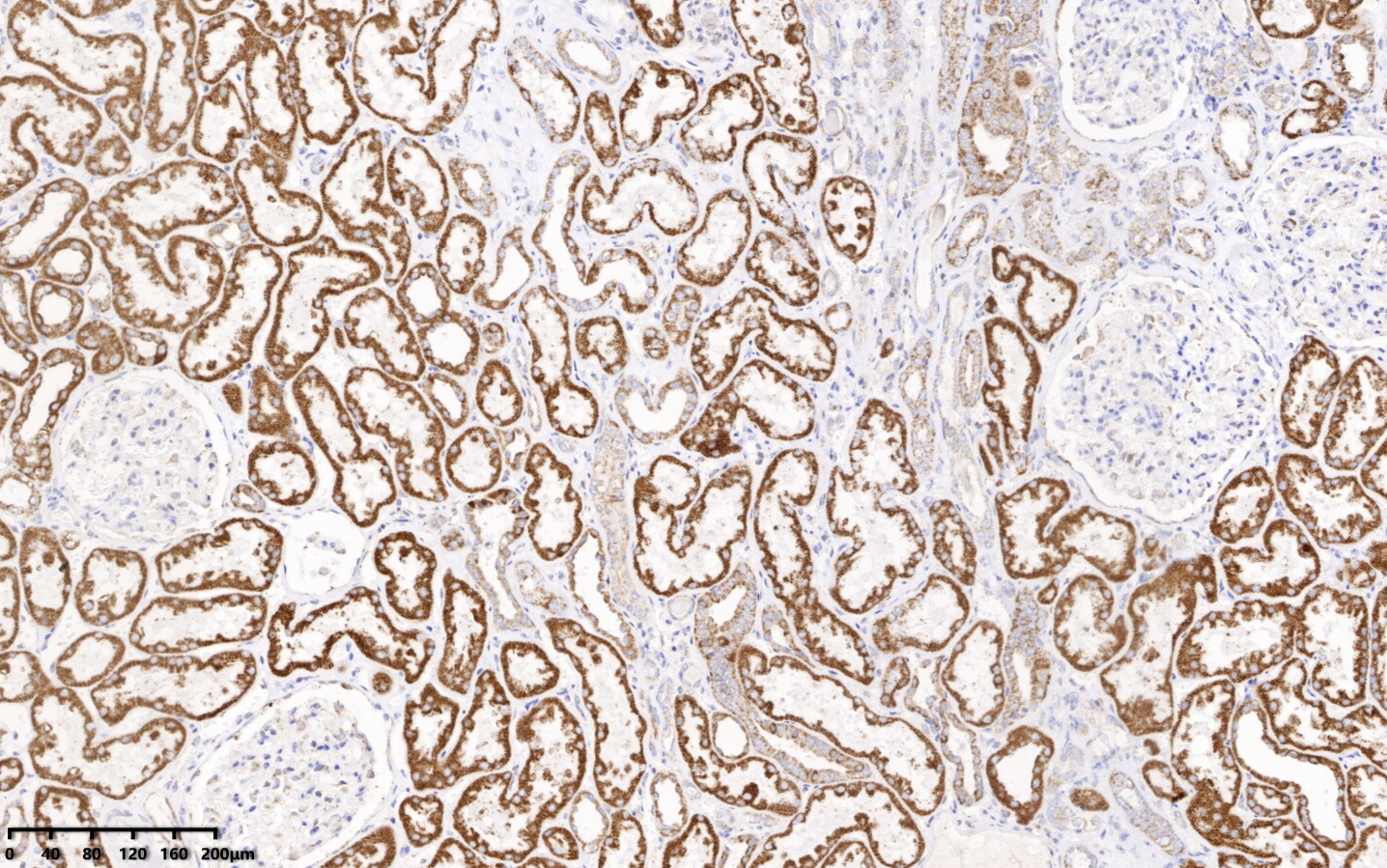

Supplement: Supplementary file 2 [file Presentation1.ZIP › Case1-P/20220820162652.tiff]

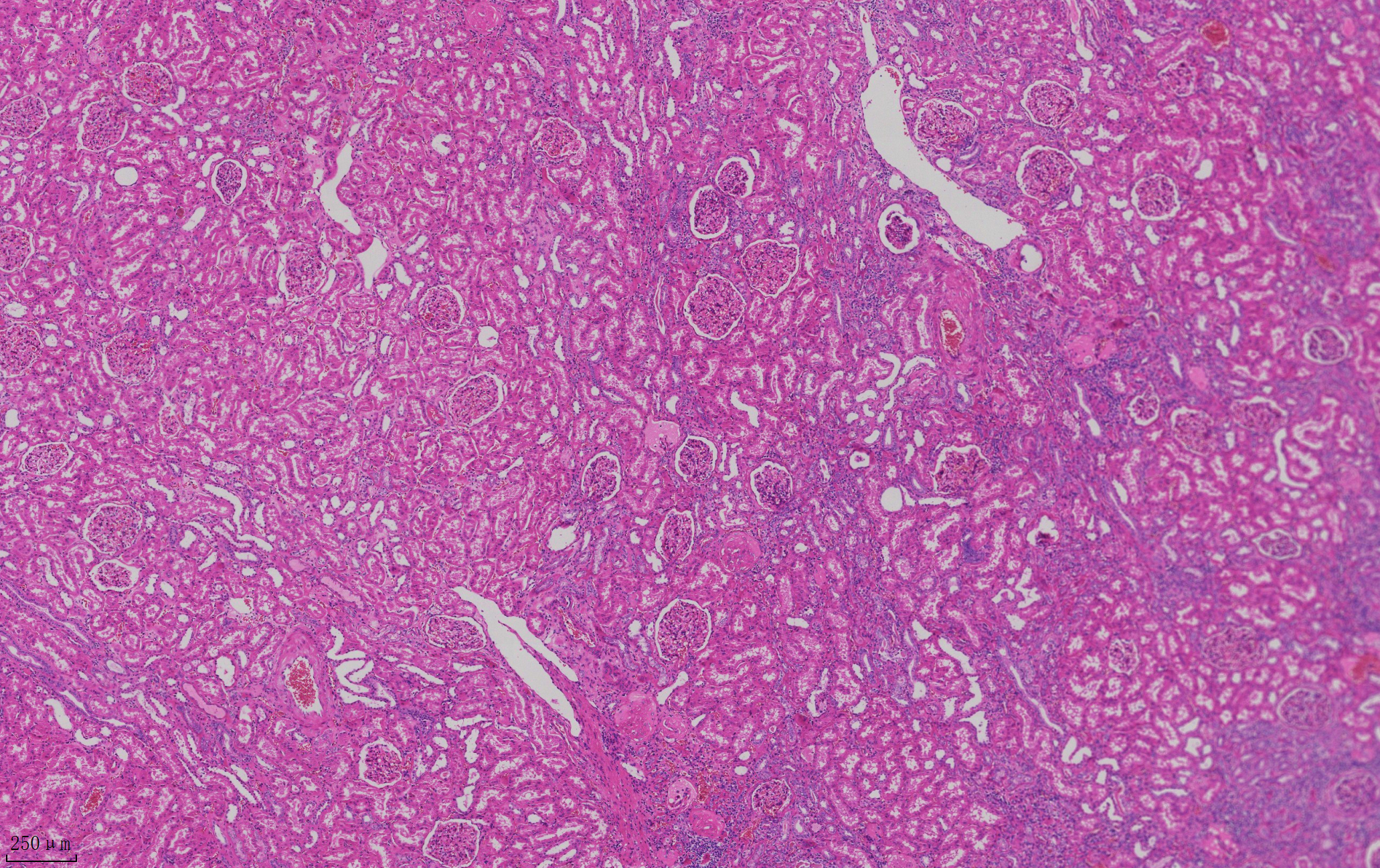

Supplement: Supplementary file 2 [file Presentation1.ZIP › Case1-P/20220820_162429.tiff]

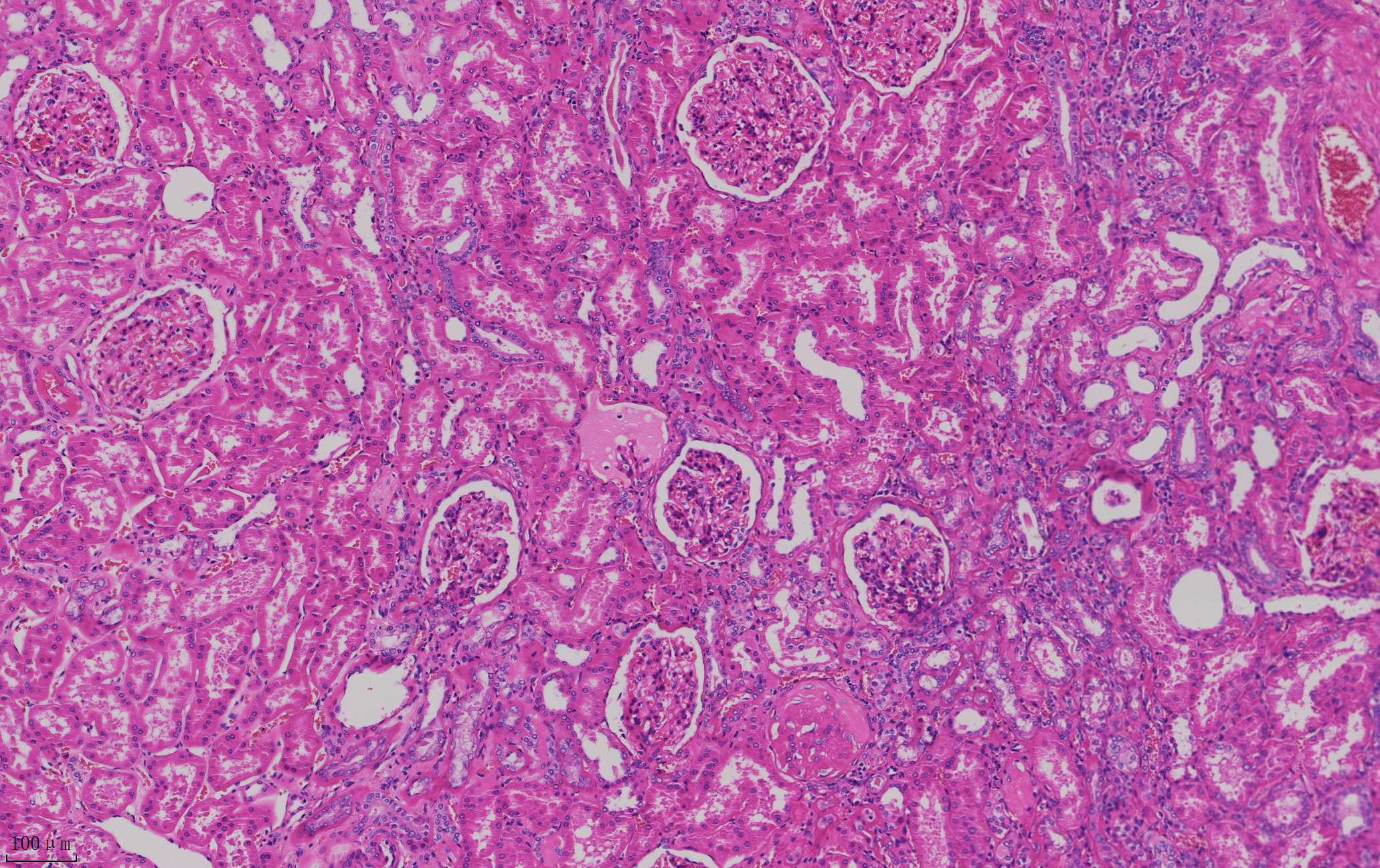

Supplement: Supplementary file 2 [file Presentation1.ZIP › Case1-P/20220820_162456.tiff]

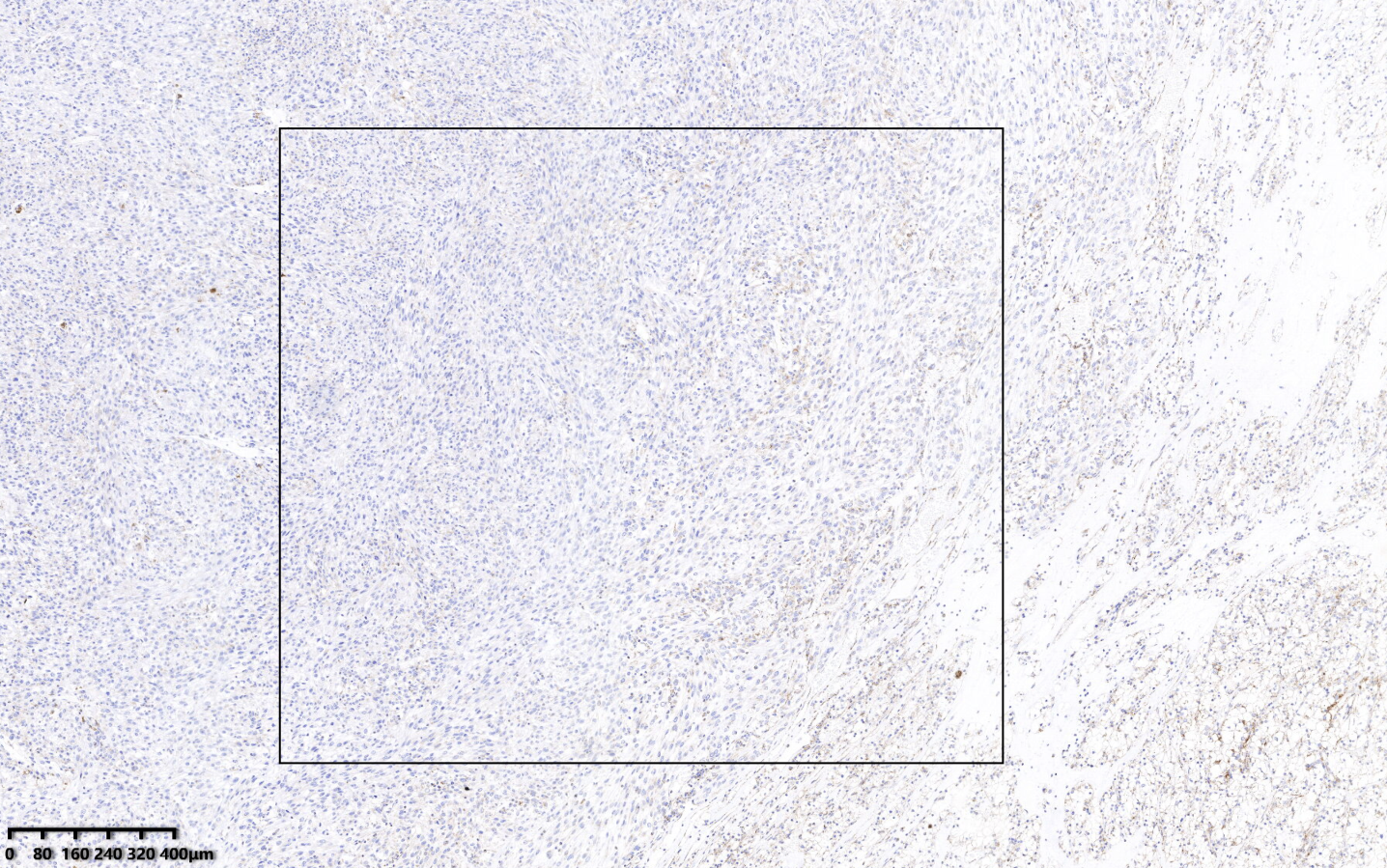

Supplement: Supplementary file 3 [file Presentation2.ZIP › Case1-T/20220820162056.tiff]

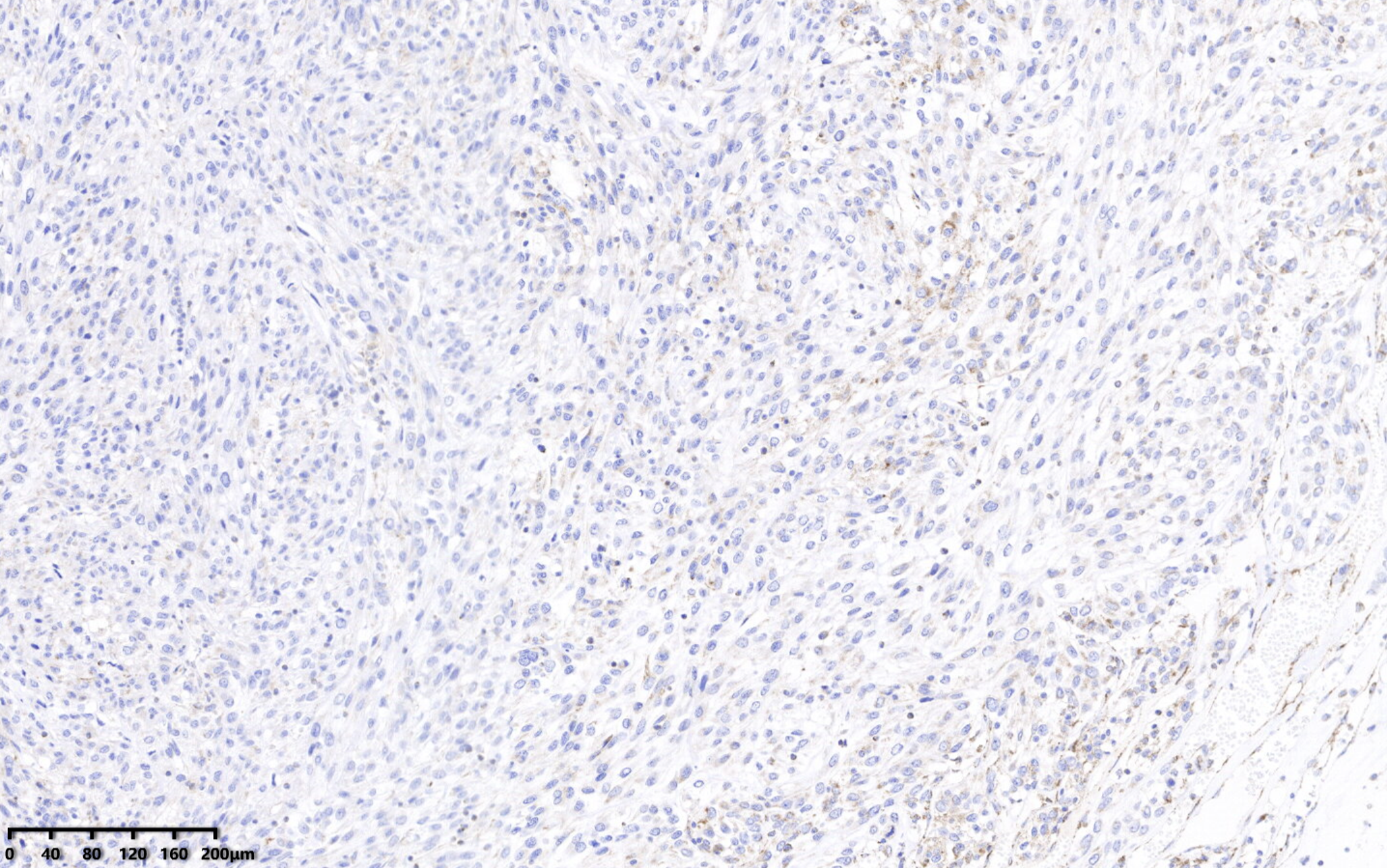

Supplement: Supplementary file 3 [file Presentation2.ZIP › Case1-T/20220820162138.tiff]

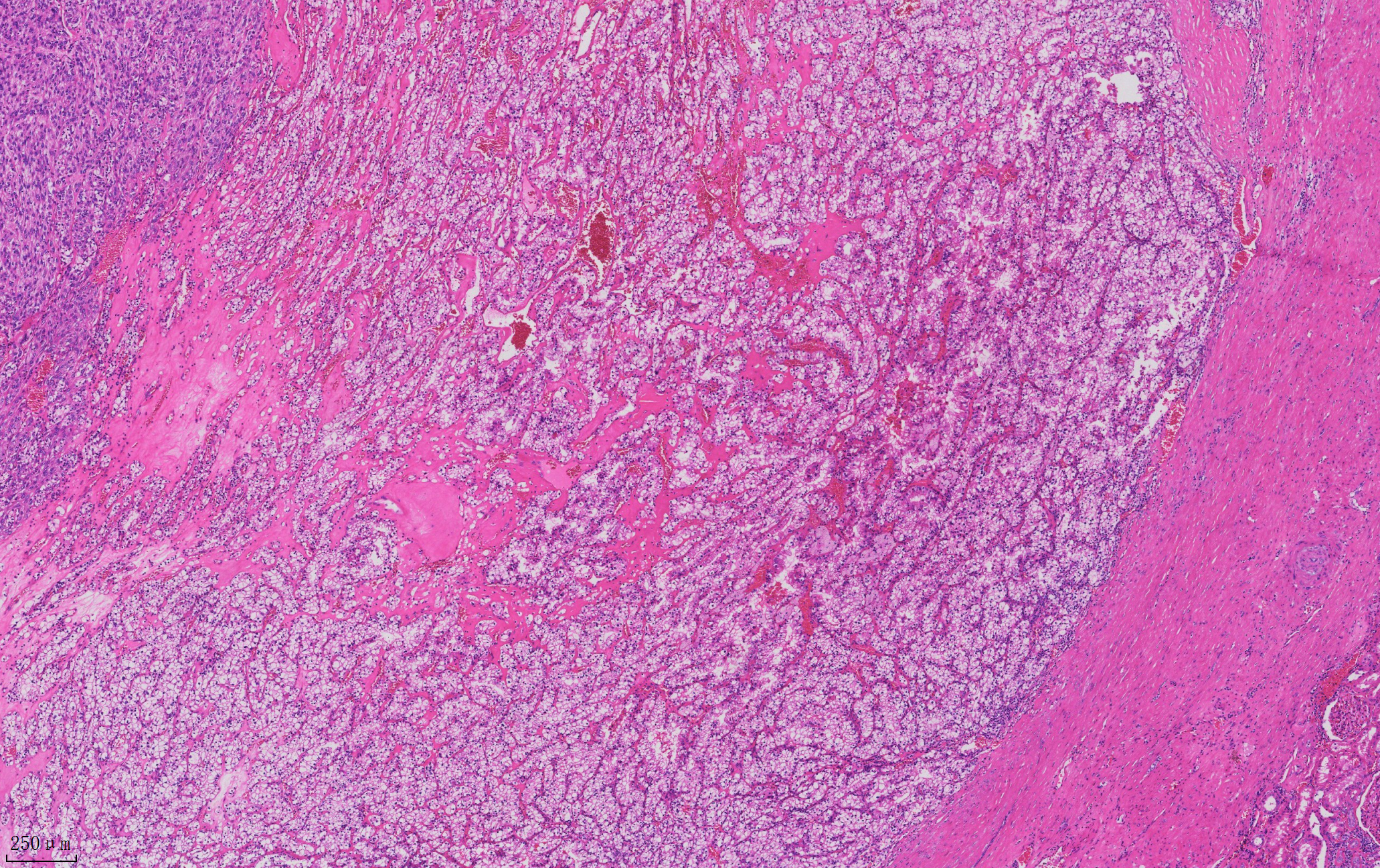

Supplement: Supplementary file 3 [file Presentation2.ZIP › Case1-T/20220820_161646.tiff]

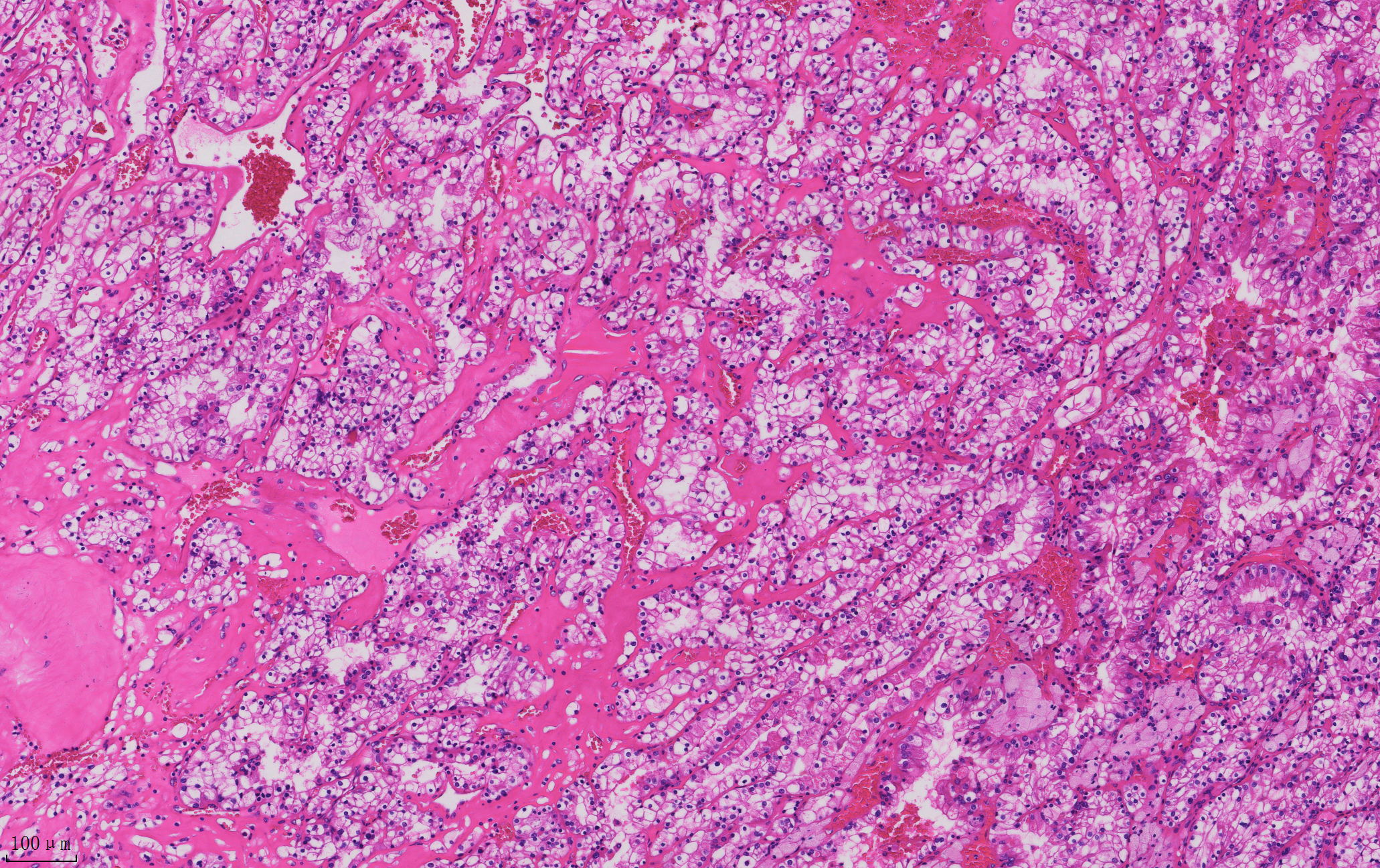

Supplement: Supplementary file 3 [file Presentation2.ZIP › Case1-T/20220820_161708.tiff]

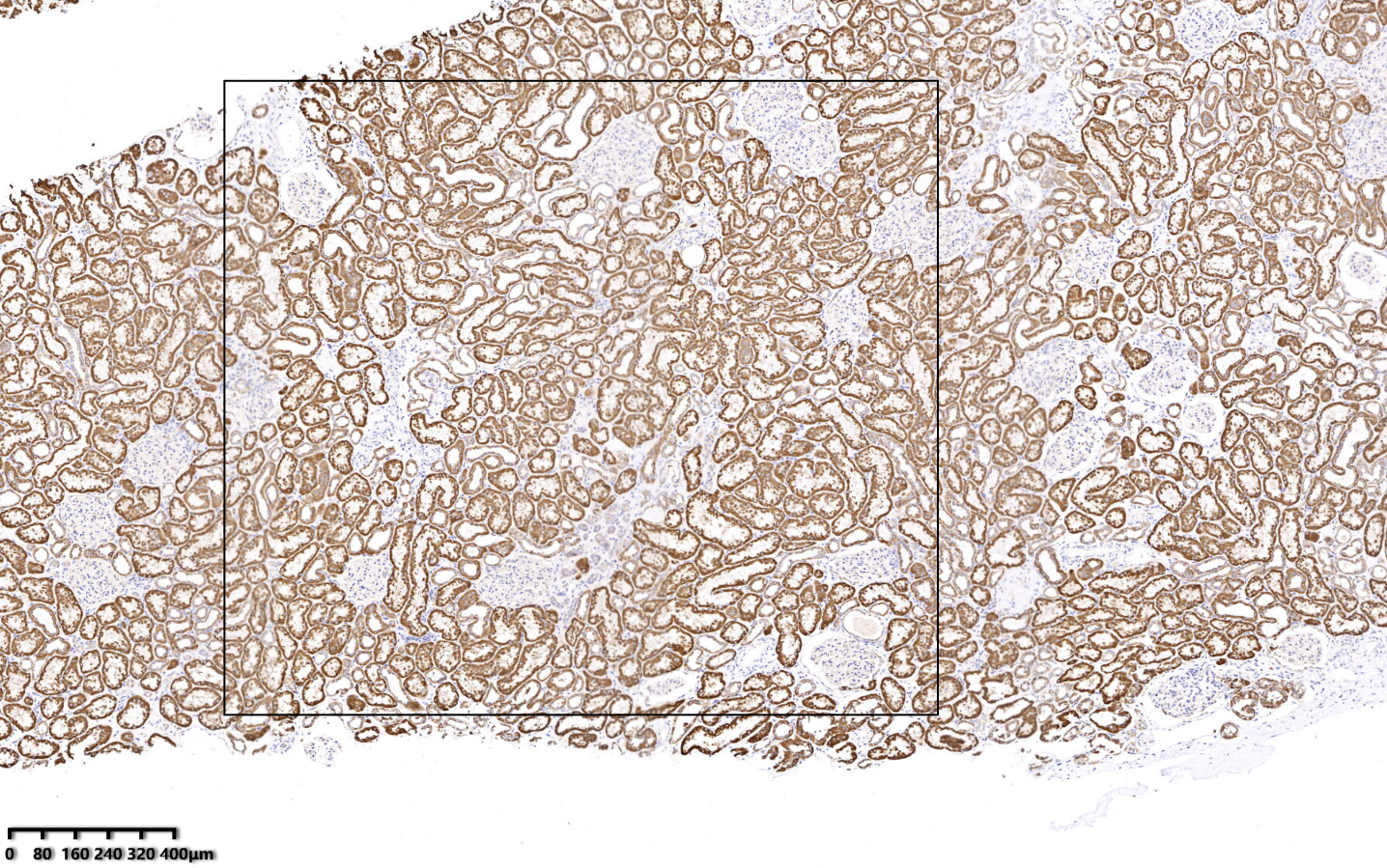

Supplement: Supplementary file 5 [file Presentation3.ZIP › Case2-P/20220819233319.tiff]

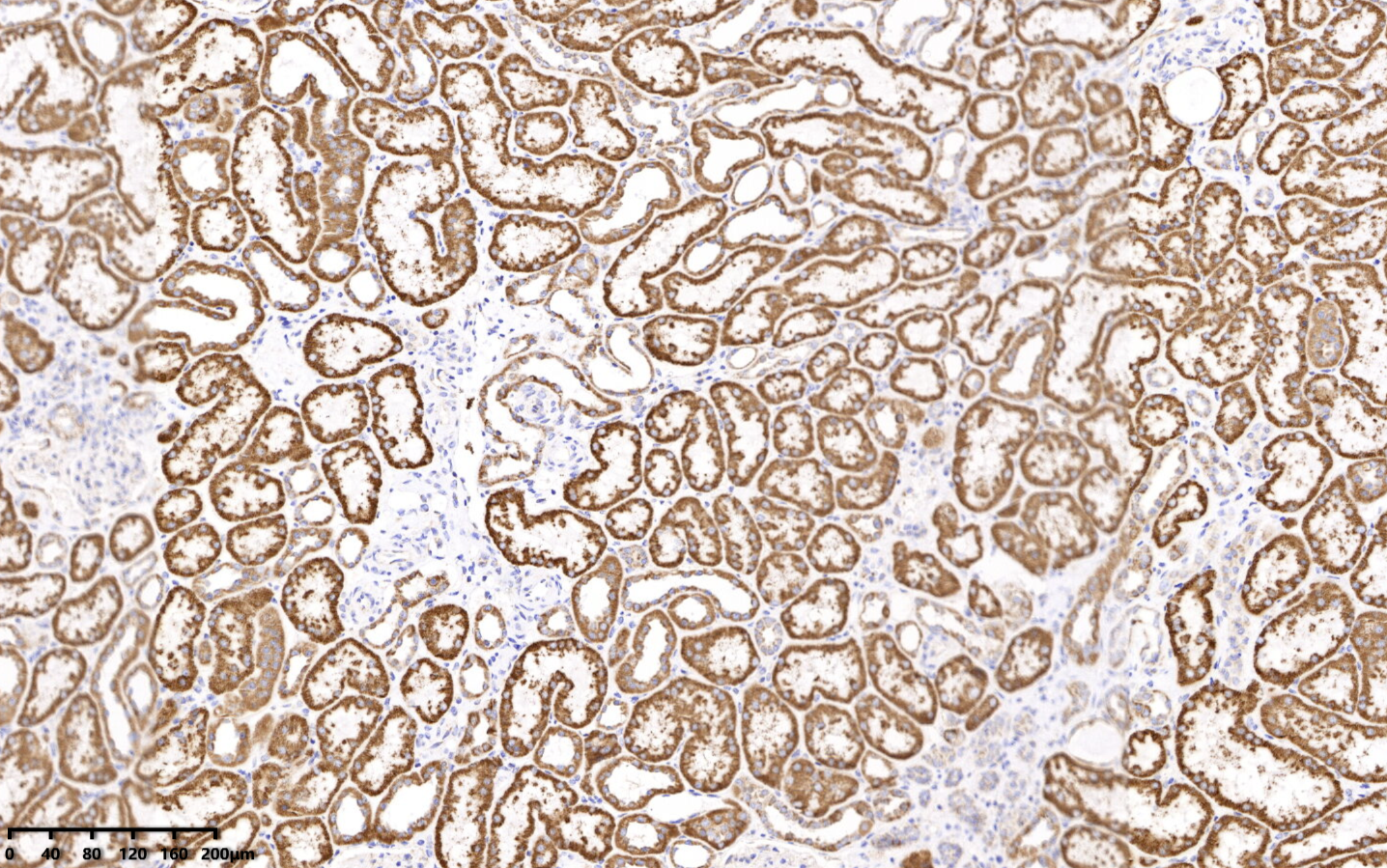

Supplement: Supplementary file 5 [file Presentation3.ZIP › Case2-P/20220819233455.tiff]

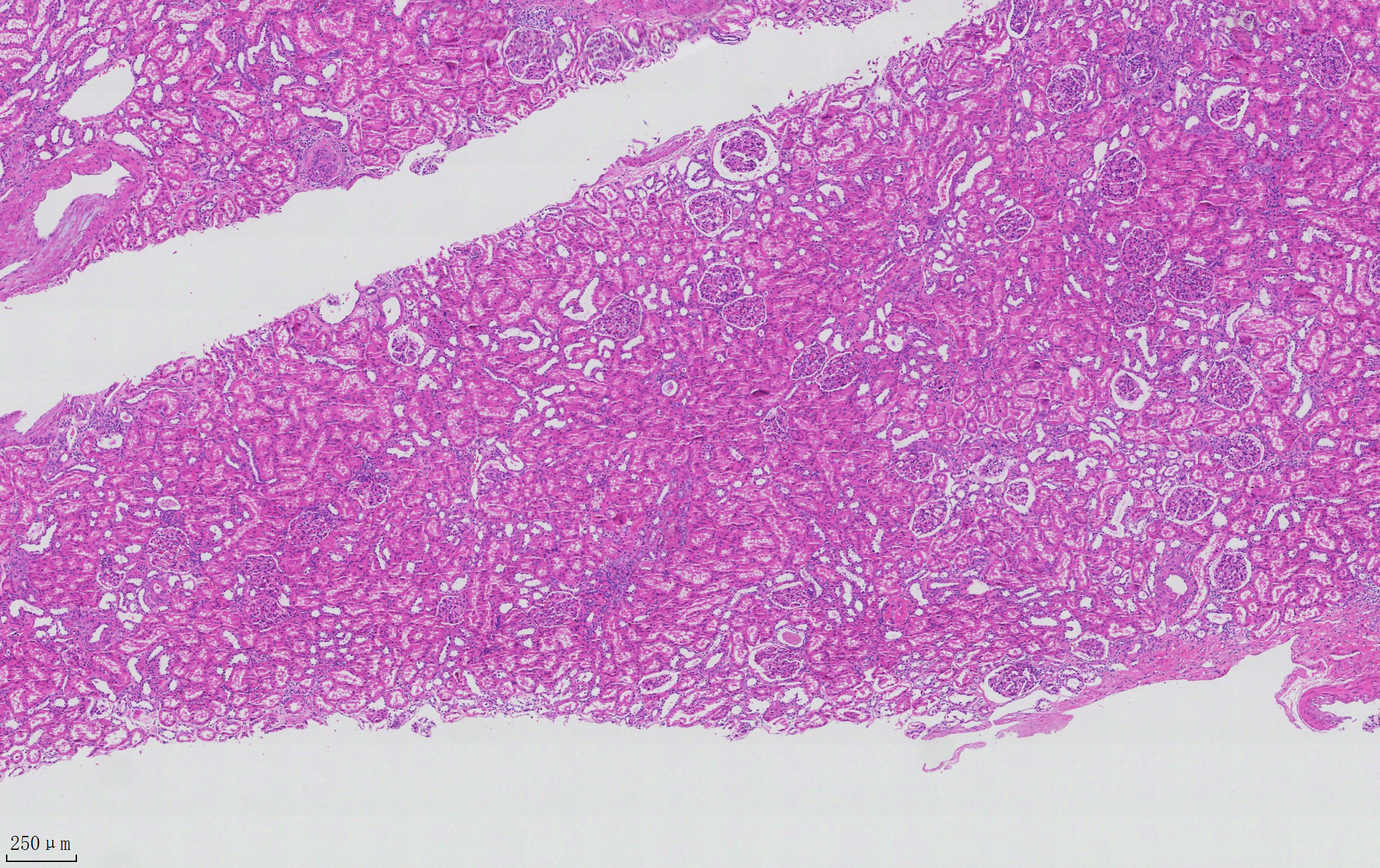

Supplement: Supplementary file 5 [file Presentation3.ZIP › Case2-P/20220819_233147.tiff]

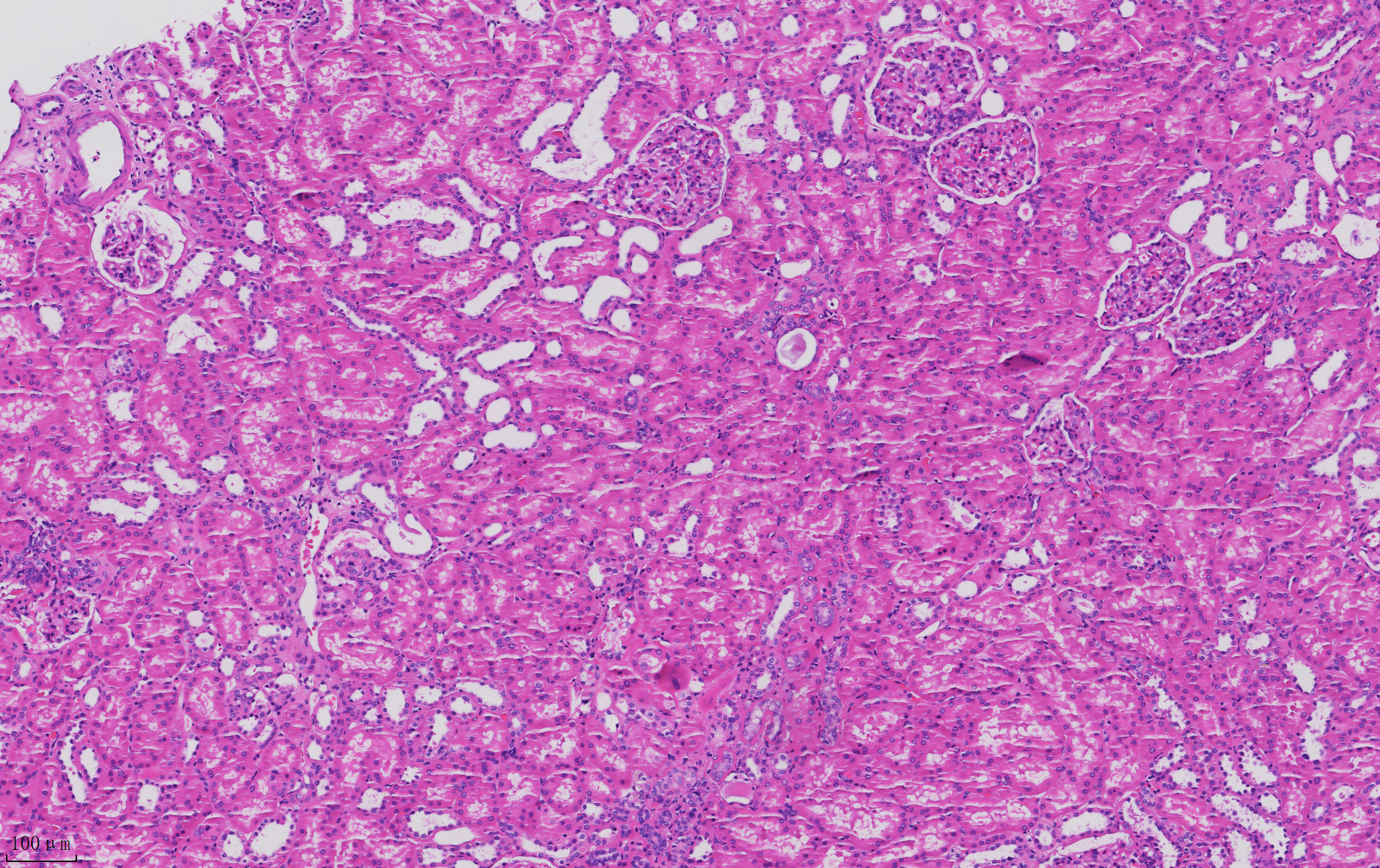

Supplement: Supplementary file 5 [file Presentation3.ZIP › Case2-P/20220819_233215.tiff]

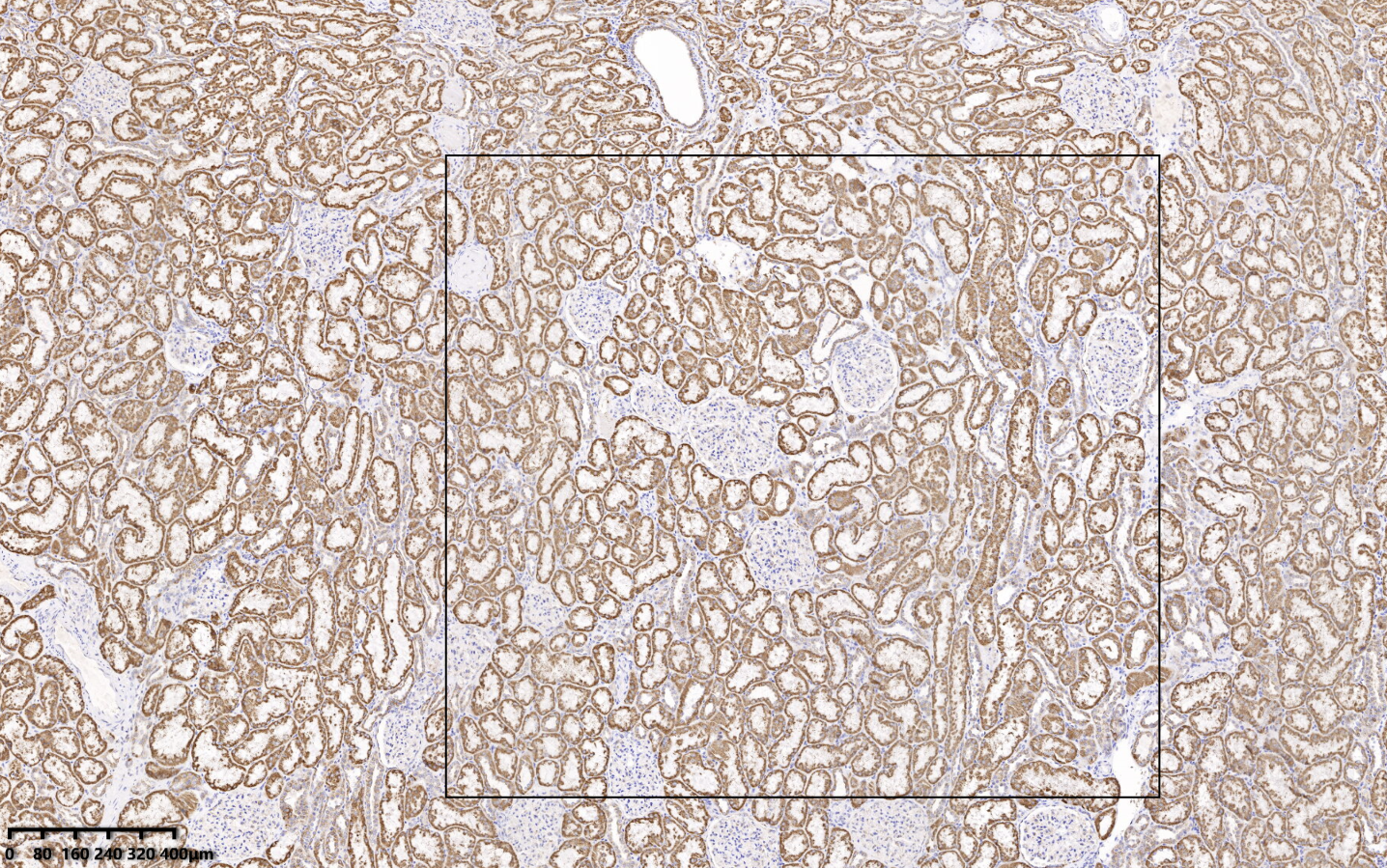

Supplement: Supplementary file 6 [file Presentation5.ZIP › Case3-P/20220819223733.tiff]

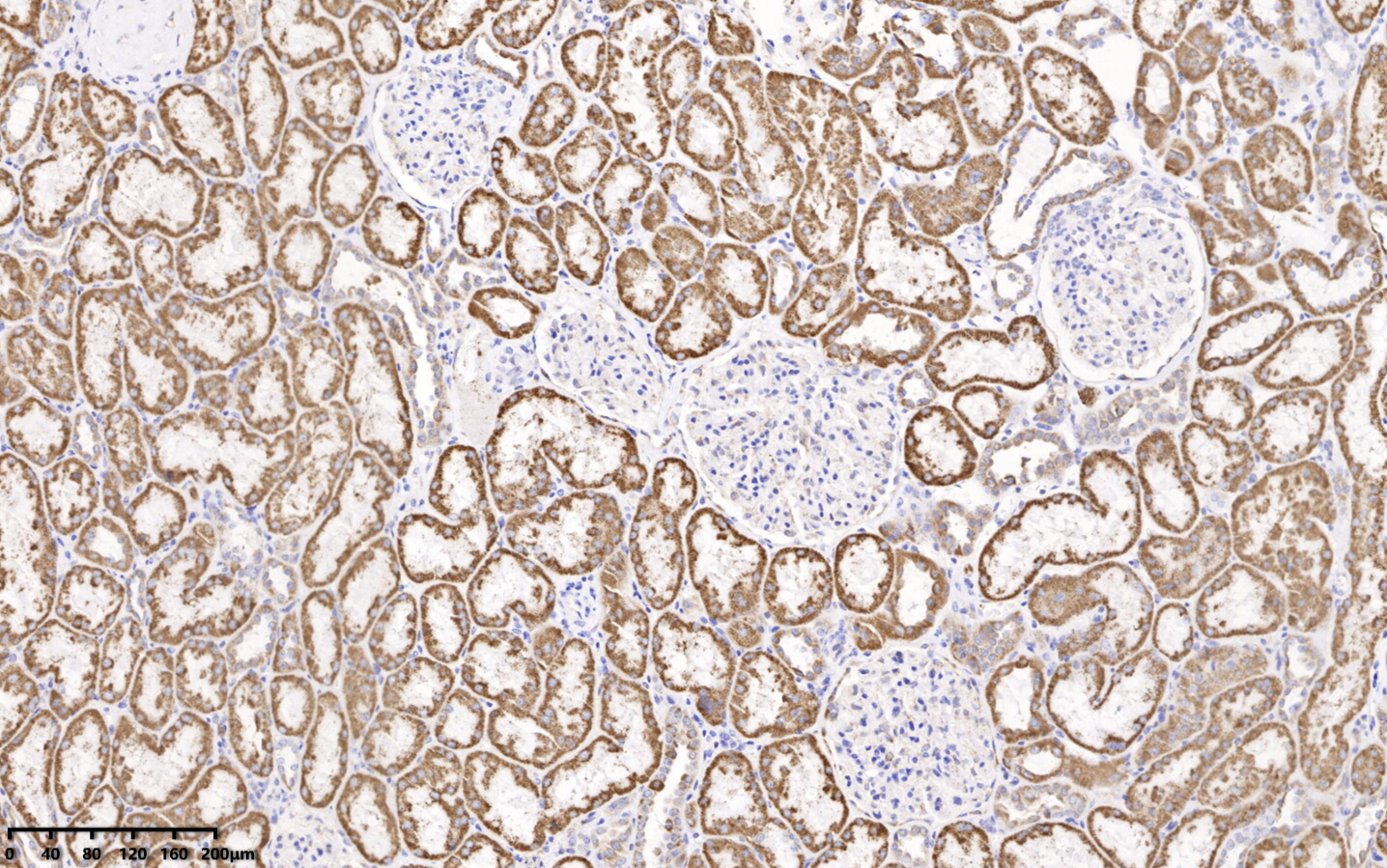

Supplement: Supplementary file 6 [file Presentation5.ZIP › Case3-P/20220819223830.tiff]

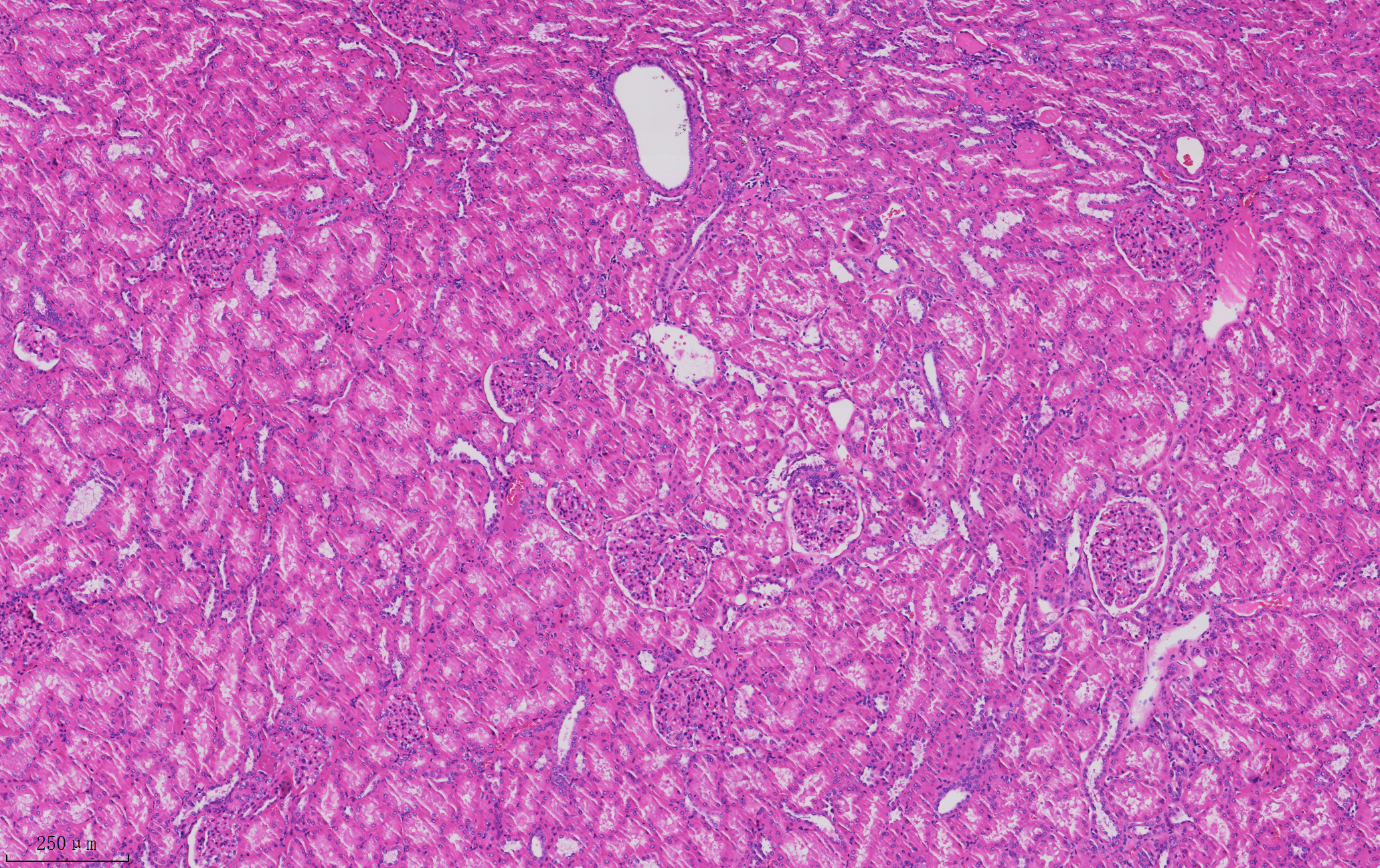

Supplement: Supplementary file 6 [file Presentation5.ZIP › Case3-P/20220819_231727.tiff]

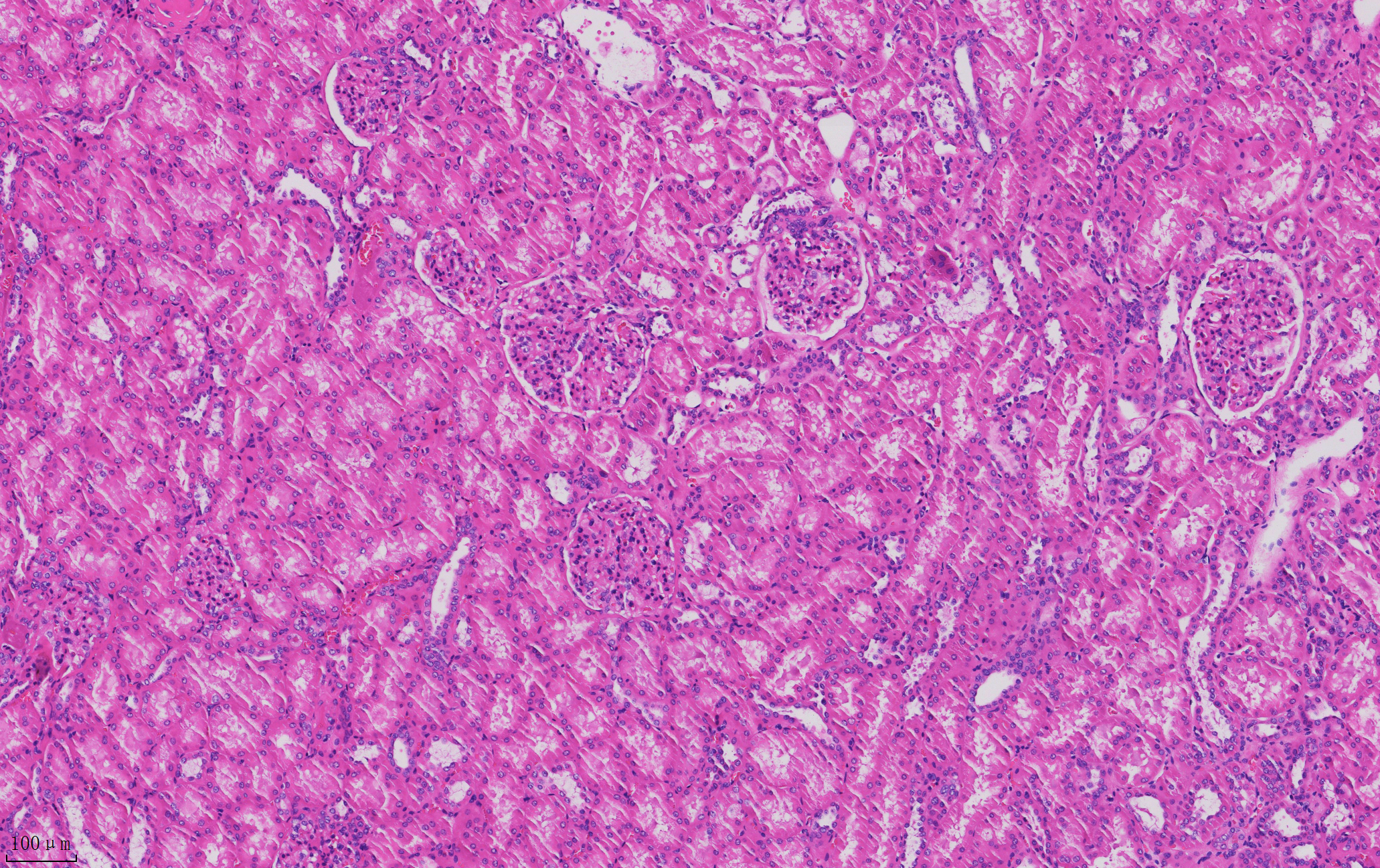

Supplement: Supplementary file 6 [file Presentation5.ZIP › Case3-P/20220819_231840.tiff]

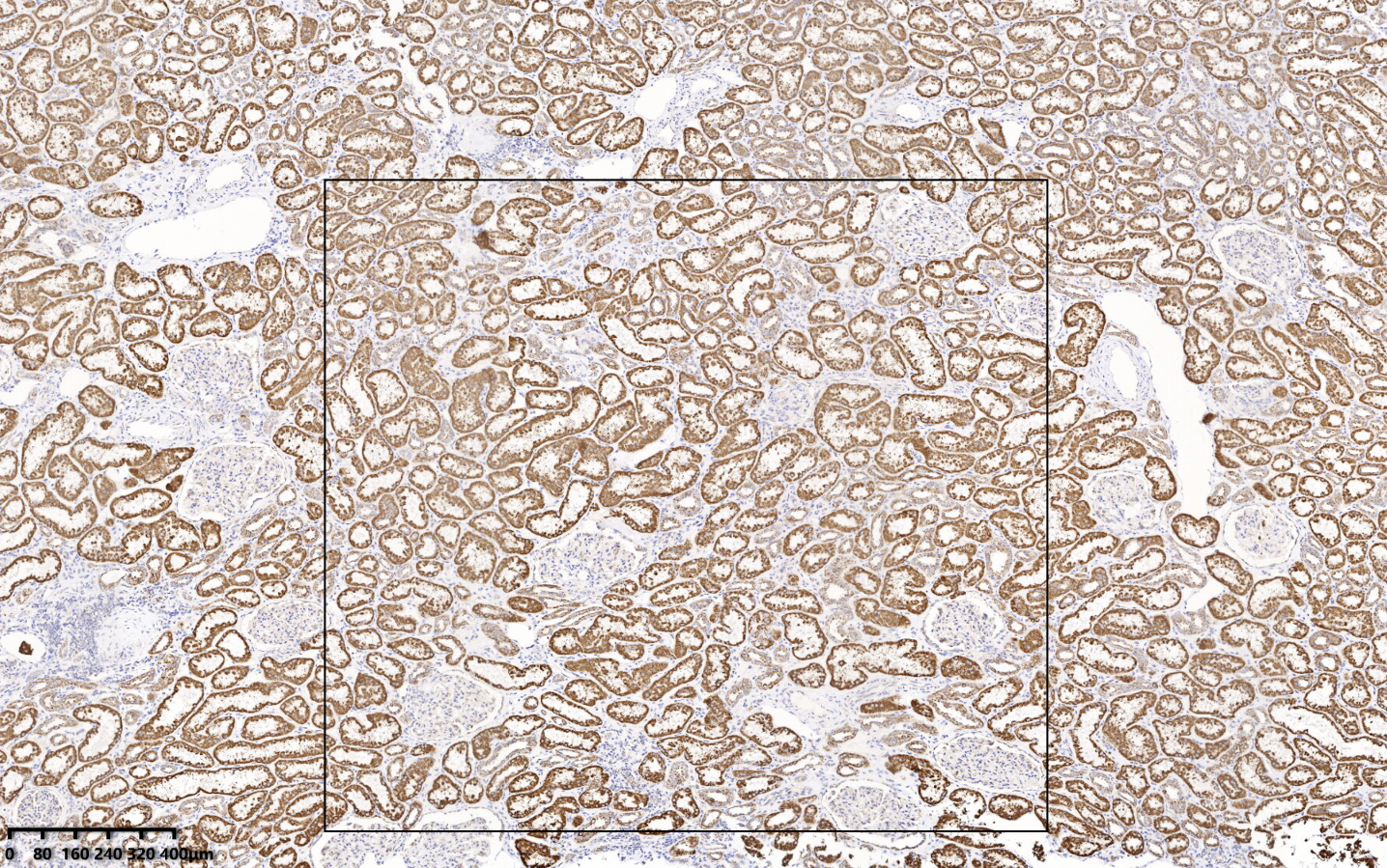

Supplement: Supplementary file 7 [file Presentation7.ZIP › Case4-P/20220819235933.tiff]

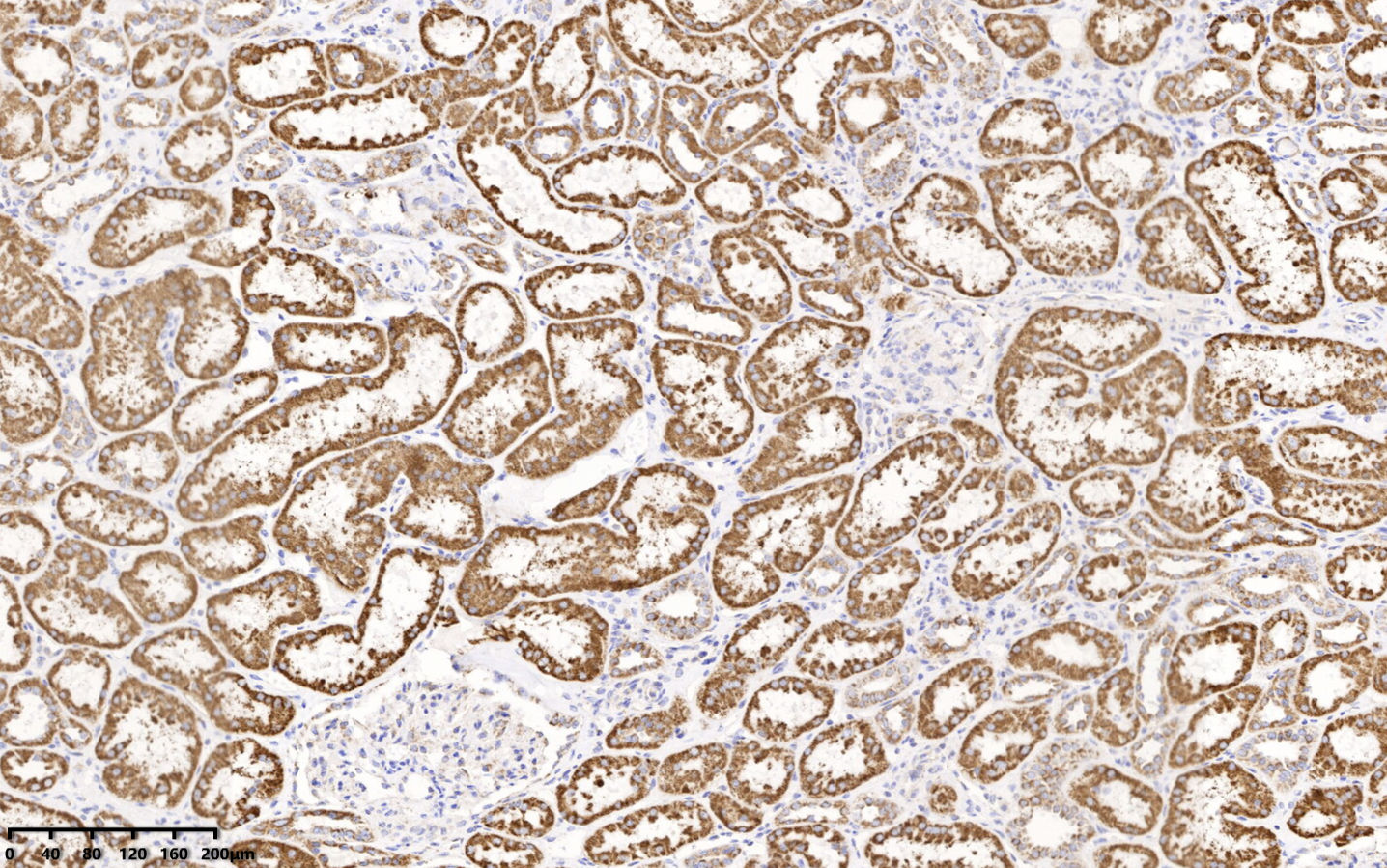

Supplement: Supplementary file 7 [file Presentation7.ZIP › Case4-P/20220819235957.tiff]

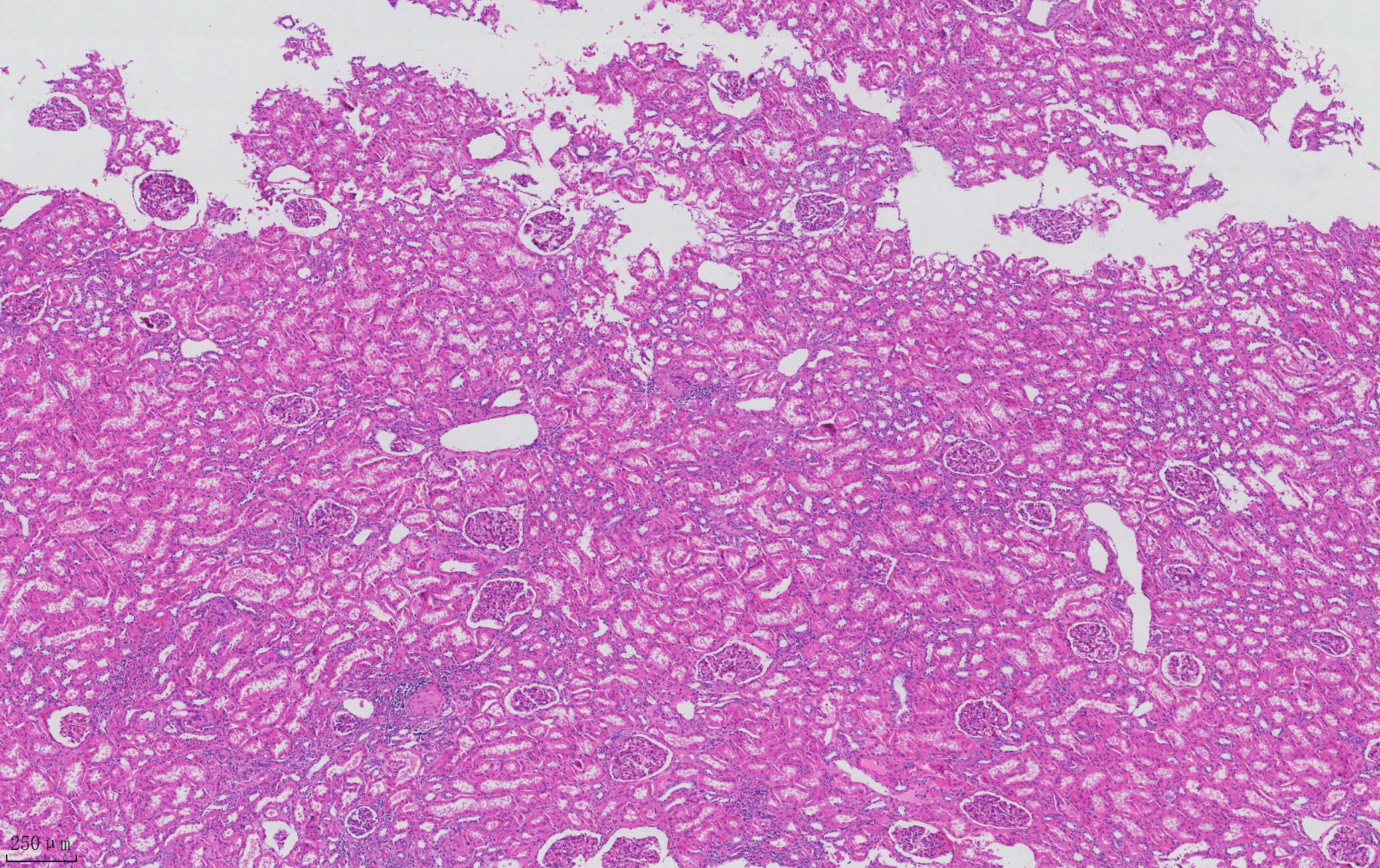

Supplement: Supplementary file 7 [file Presentation7.ZIP › Case4-P/20220819_235759.tiff]

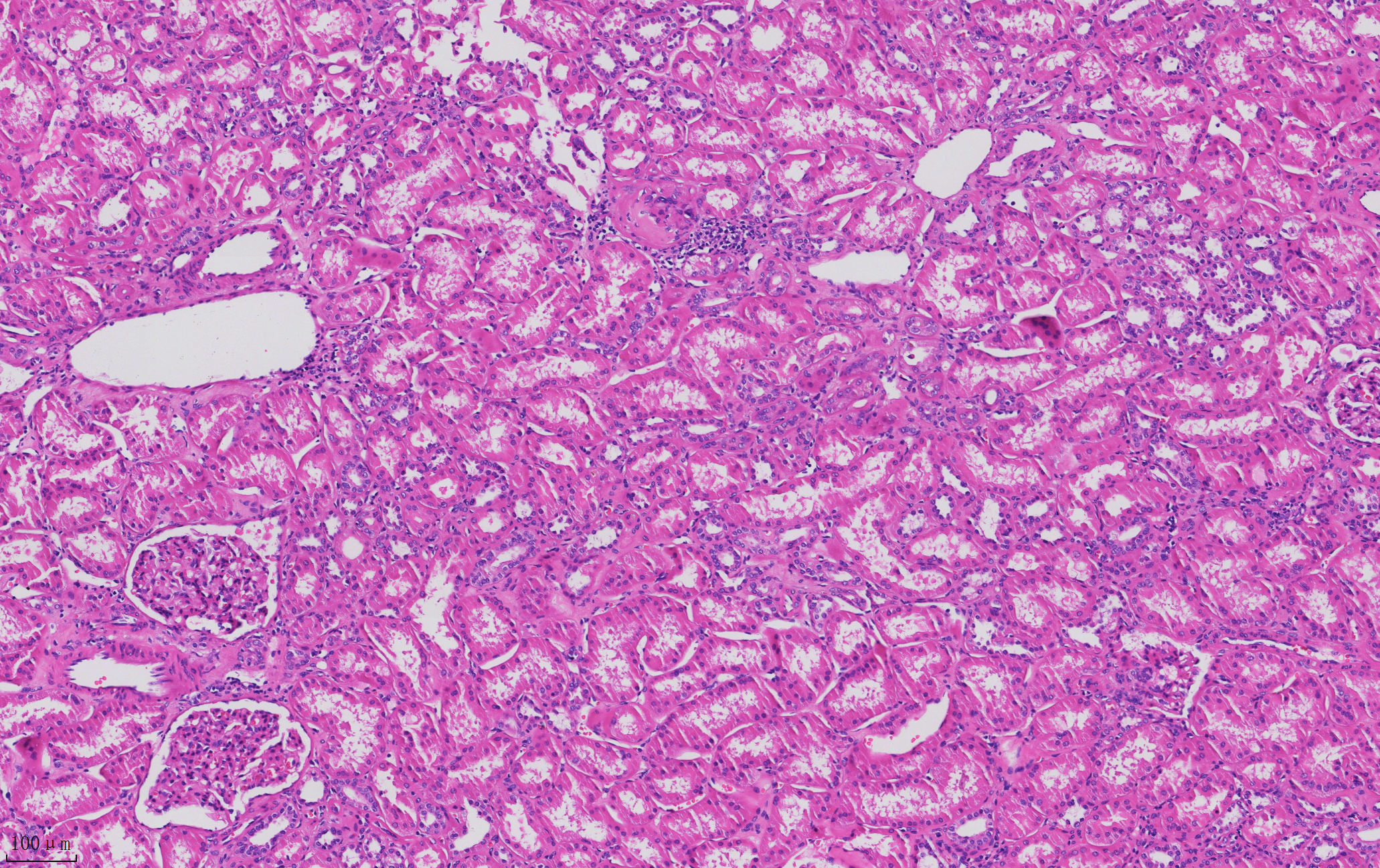

Supplement: Supplementary file 7 [file Presentation7.ZIP › Case4-P/20220819_235828.tiff]

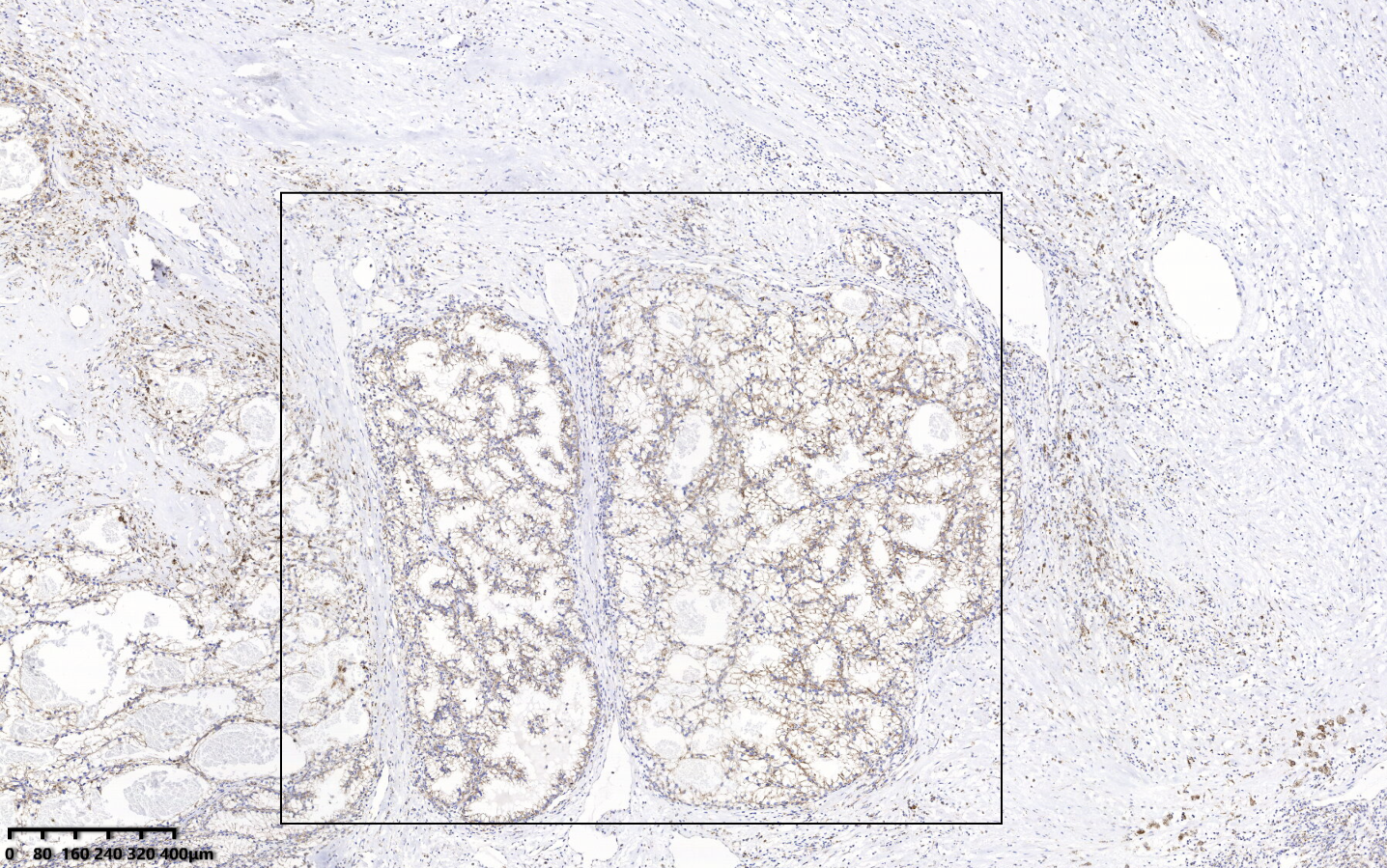

Supplement: Supplementary file 8 [file Presentation8.ZIP › Case4-T/20220819235351.tiff]

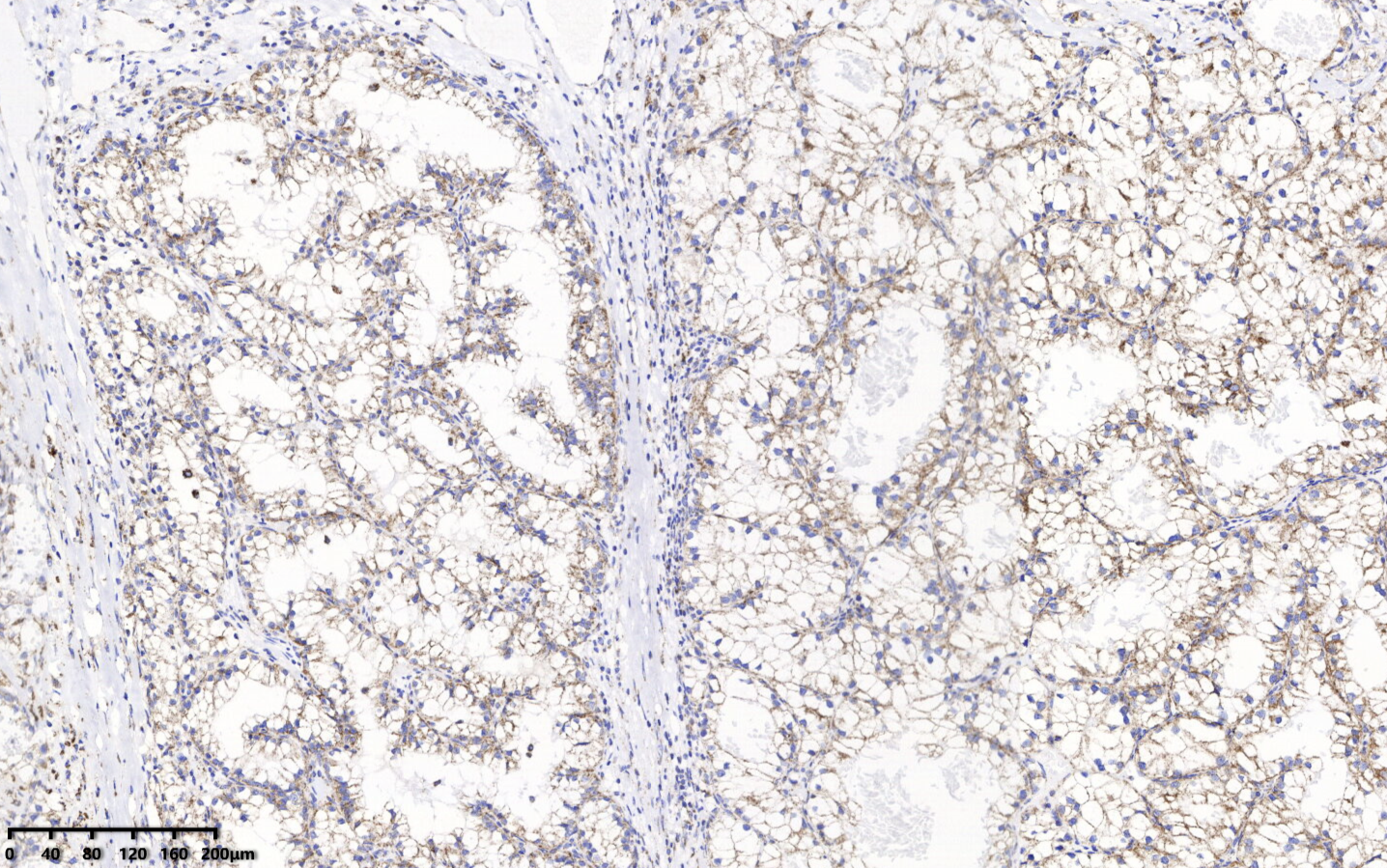

Supplement: Supplementary file 8 [file Presentation8.ZIP › Case4-T/20220819235420.tiff]

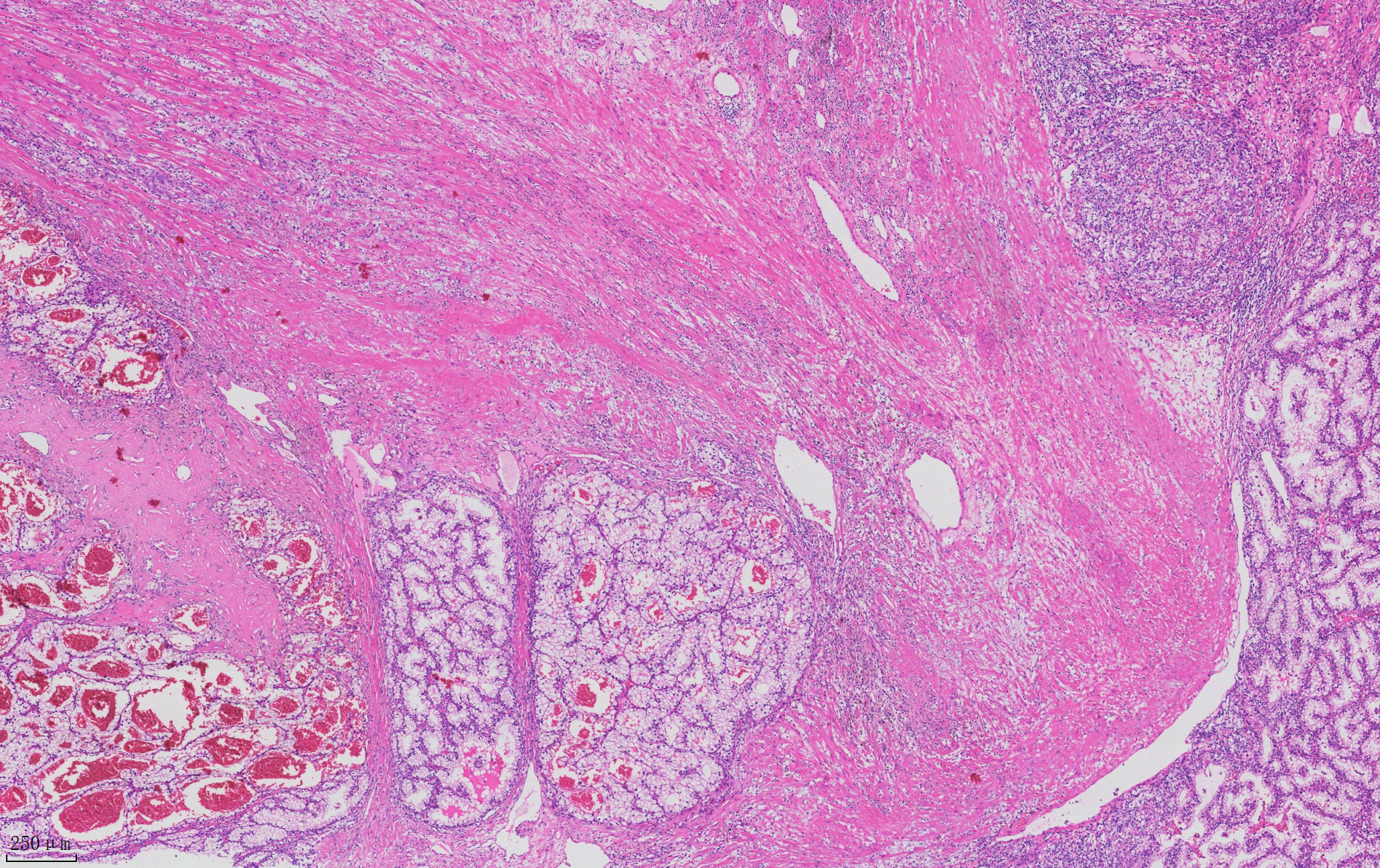

Supplement: Supplementary file 8 [file Presentation8.ZIP › Case4-T/20220819_235142.tiff]

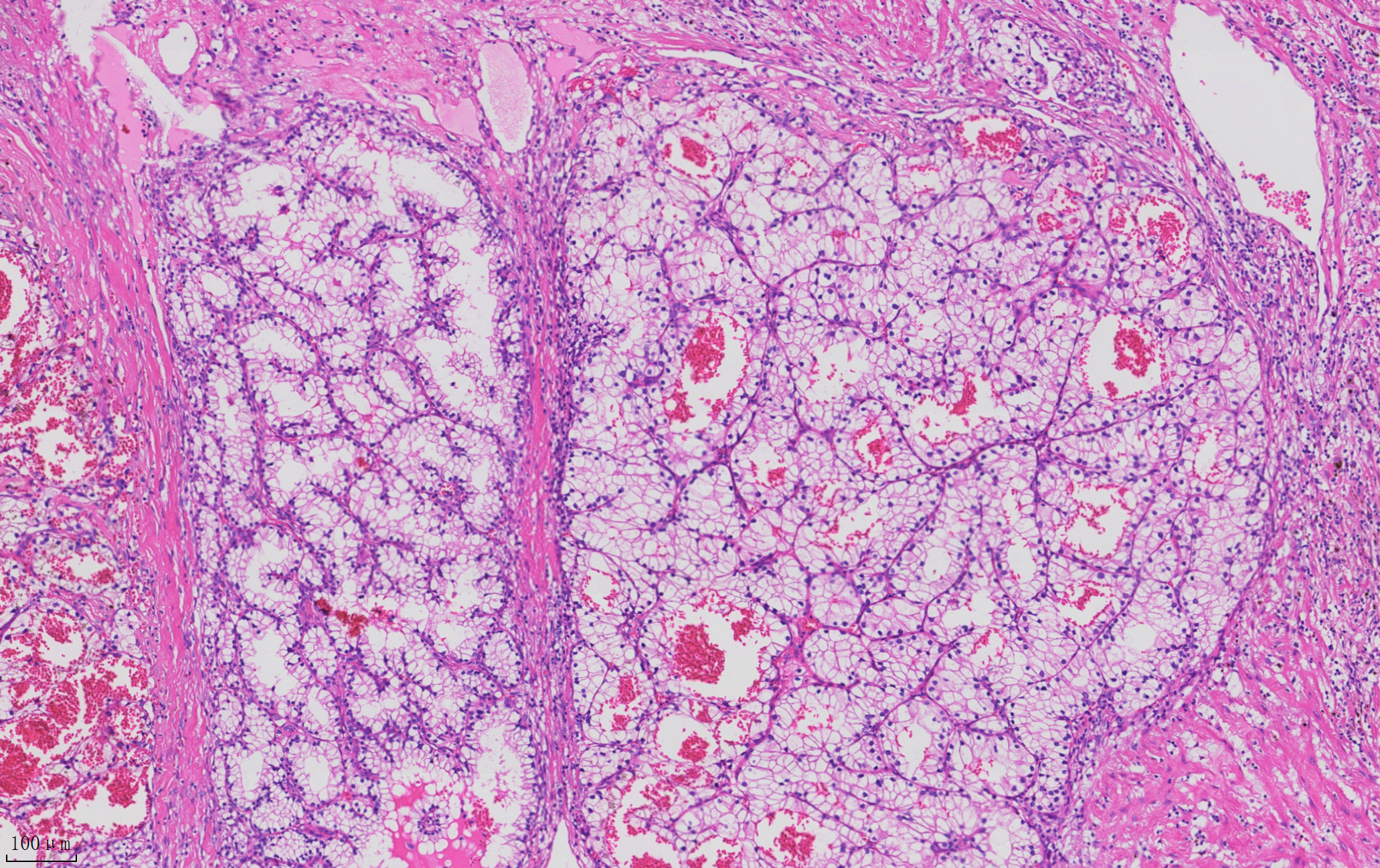

Supplement: Supplementary file 8 [file Presentation8.ZIP › Case4-T/20220819_235228.tiff]

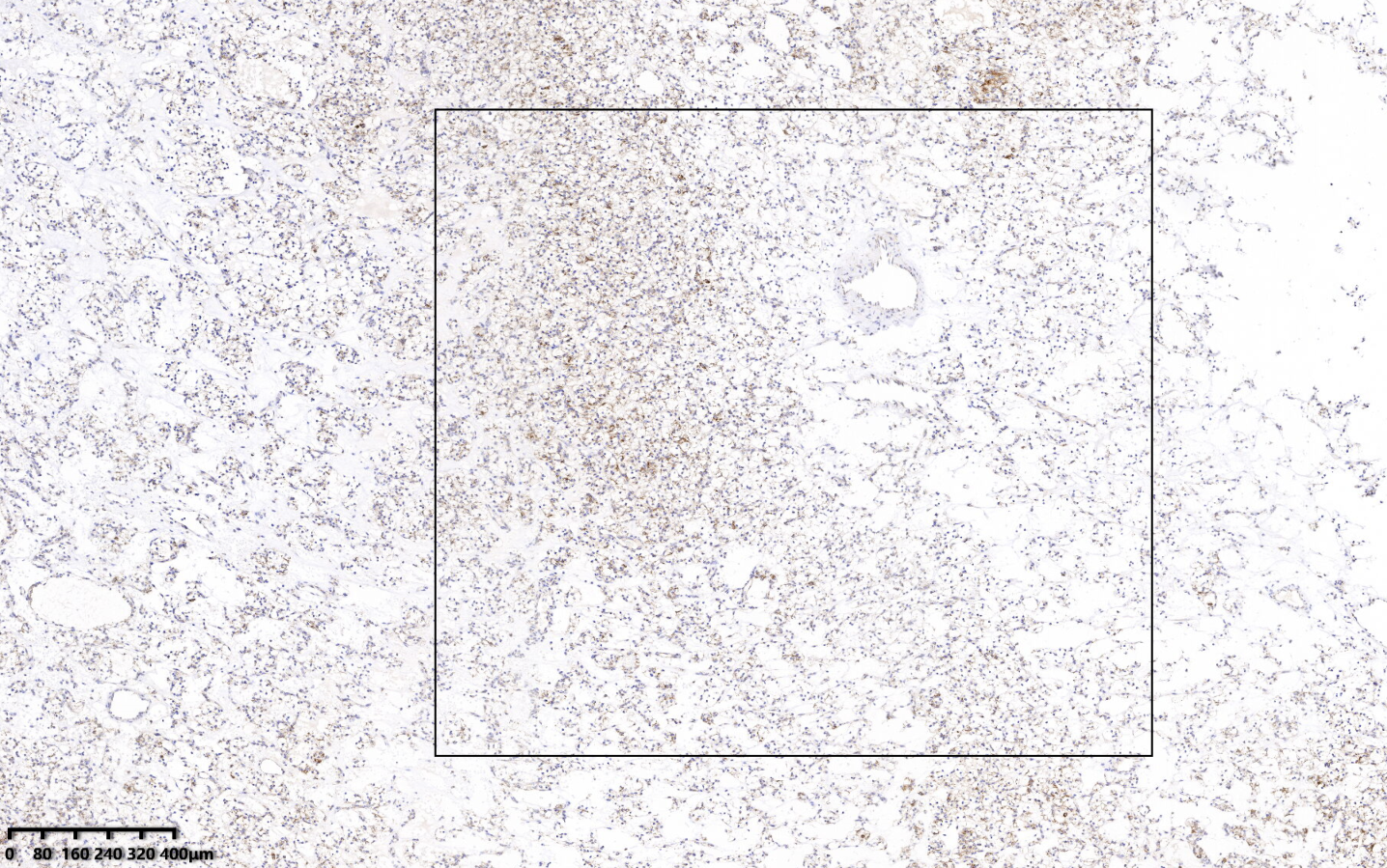

Supplement: Supplementary file 9 [file Presentation6.ZIP › Case3-T/20220819223046.tiff]

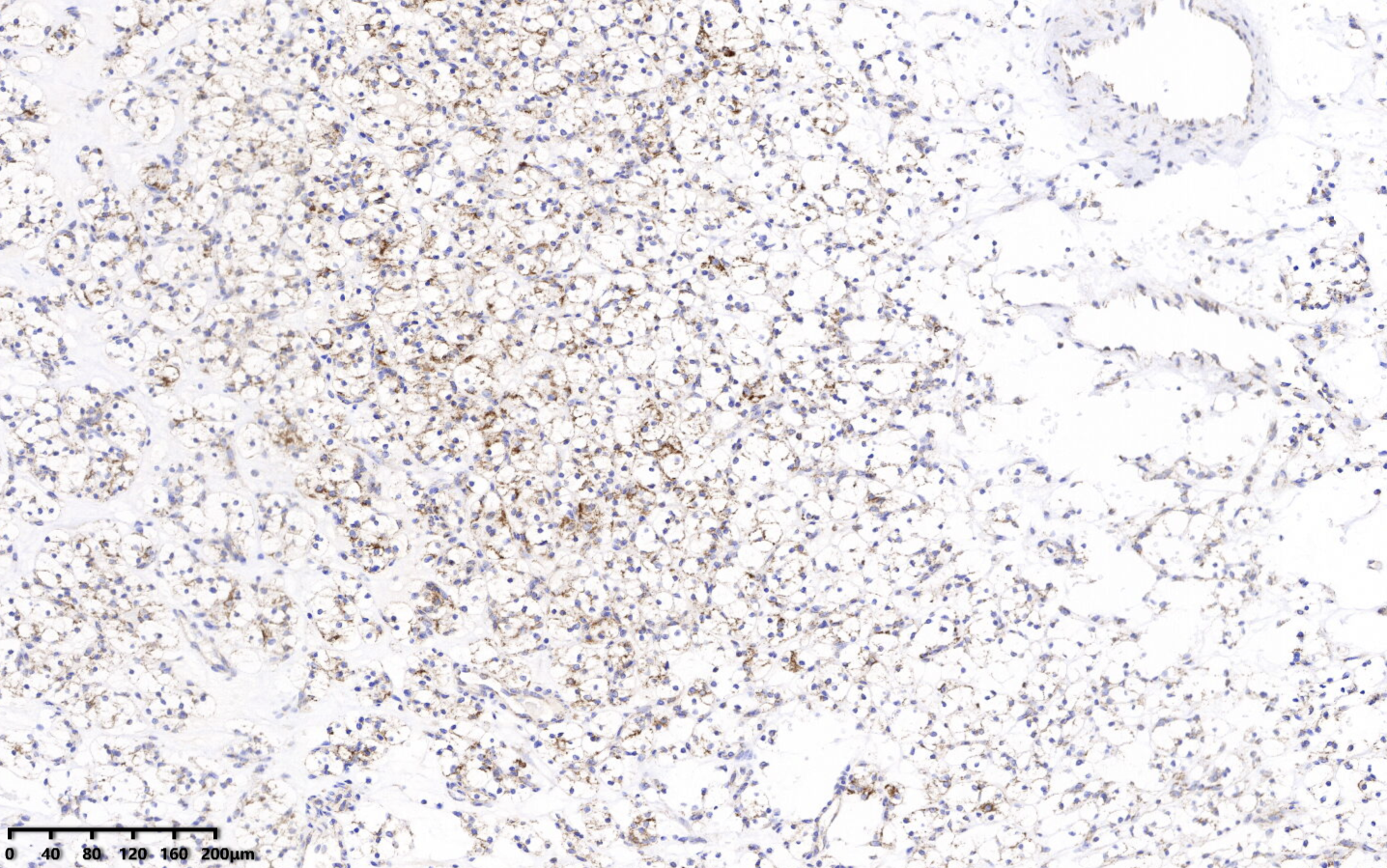

Supplement: Supplementary file 9 [file Presentation6.ZIP › Case3-T/20220819223114.tiff]

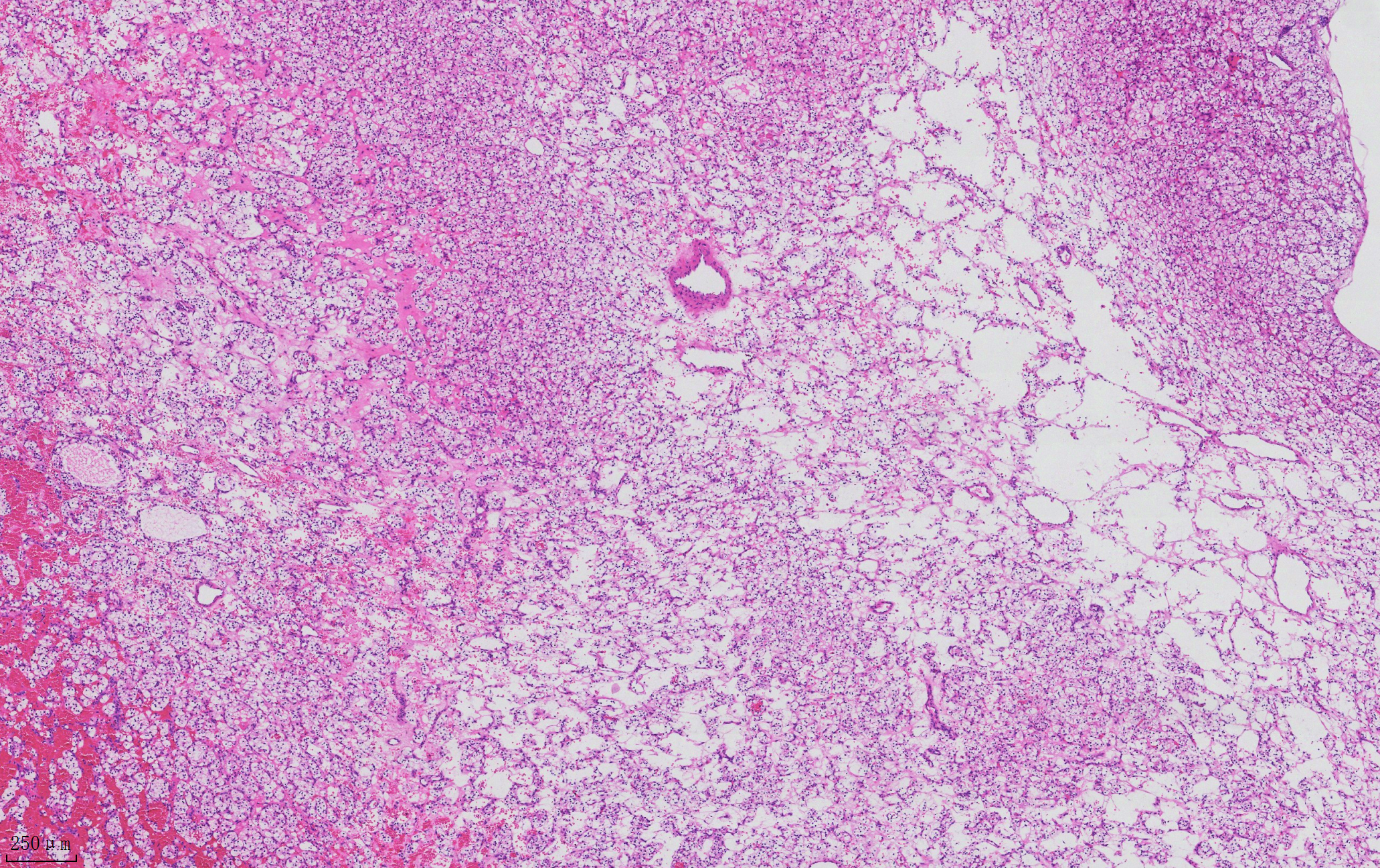

Supplement: Supplementary file 9 [file Presentation6.ZIP › Case3-T/20220819_225611.tiff]

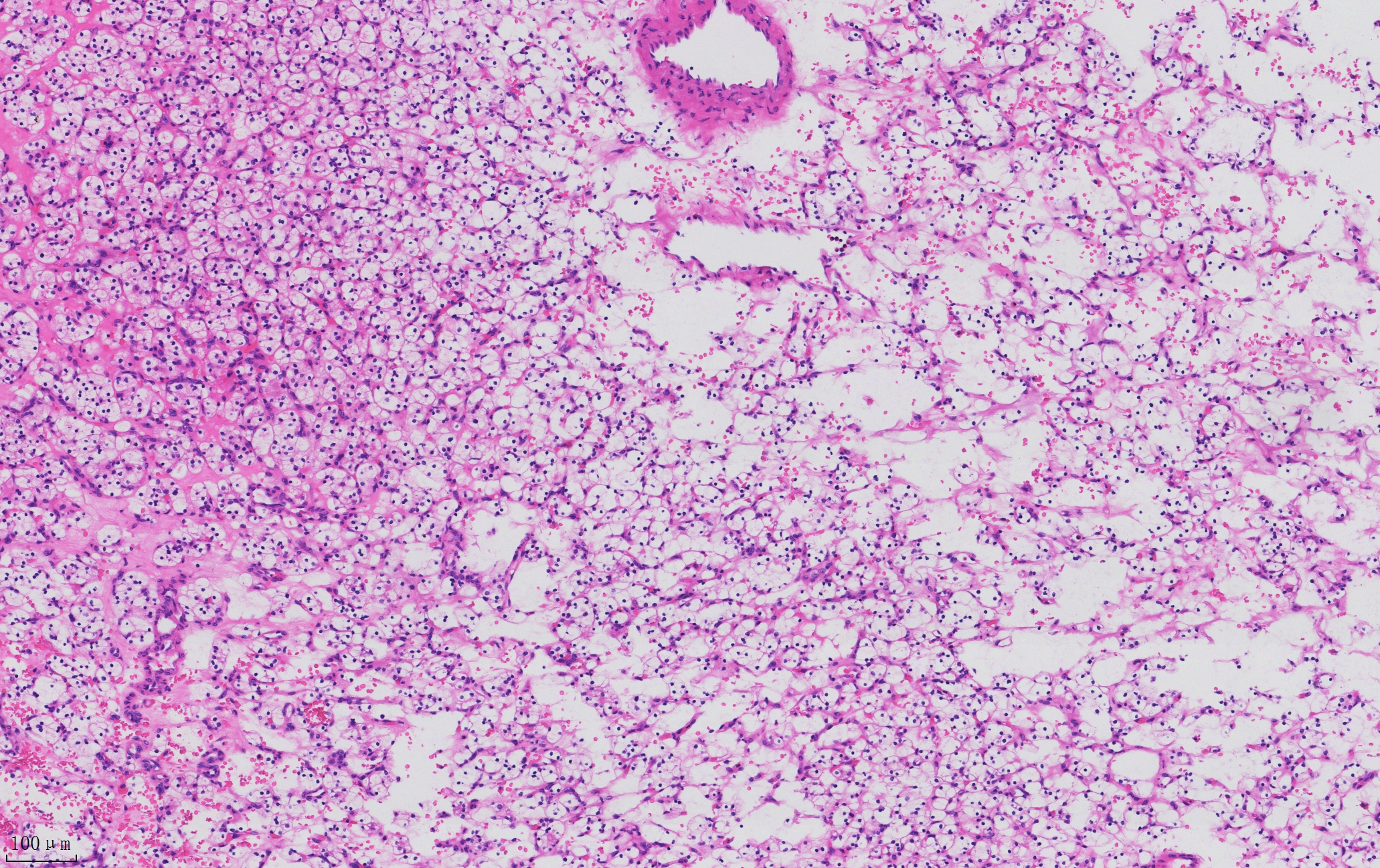

Supplement: Supplementary file 9 [file Presentation6.ZIP › Case3-T/20220819_225635.tiff]
