# Supplementary material for: Cuproptosis-related gene FDX1 as a prognostic biomarker for kidney renal clear cell carcinoma correlates with immune checkpoints and immune cell infiltration
Source: Front Genet. 2023 Jan 23;14:1071694. doi: 10.3389/fgene.2023.1071694 (PMC9900009; doi:10.3389/fgene.2023.1071694)
Supplement: Supplementary file 4 [file DataSheet1.ZIP › Additional files/R packages.docx]

1. Figure 1 Related R packages:

**A：**
# veen

library(tidyverse)

library(ggplot2)

## 2 group ##

# a <- dat[,1][!is.na(dat[,1])]

# b <- dat[,2][!is.na(dat[,2])]

# A <- setdiff(a, b)

# B <- setdiff(b, a)

# AB <- intersect(a, b)

# items <- list(A = A, B = B, AB = AB)

## 3 group ##

# a <- dat[,1][!is.na(dat[,1])]

# b <- dat[,2][!is.na(dat[,2])]

# c <- dat[,3][!is.na(dat[,3])]

# A <- setdiff(a, union(b, c))

# B <- setdiff(b, union(a, c))

# C <- setdiff(c, union(a, b))

# AB <- setdiff(intersect(a, b), c)

# AC <- setdiff(intersect(a, c), b)

# BC <- setdiff(intersect(b, c), a)

# ABC <- intersect(intersect(a, b), c)

# items <- list(A = A, B = B, C = C,

# AB = AB, AC = AC, BC = BC,

# ABC = ABC)

## 4 group ##

# A <- setdiff(a, union(union(b, c), d))

# B <- setdiff(b, union(union(a, c), d))

# C <- setdiff(c, union(union(b, a), d))

# D <- setdiff(d, union(union(b, a), c))

# AB <- setdiff(intersect(a, b), union(c, d))

# AC <- setdiff(intersect(a, c), union(b, d))

# AD <- setdiff(intersect(a, d), union(c, b))

# BC <- setdiff(intersect(c, b), union(a, d))

# BD <- setdiff(intersect(d, b), union(c, a))

# CD <- setdiff(intersect(c, d), union(a, b))

# ABC <- setdiff(intersect(intersect(a, b), c), d)

# ABD <- setdiff(intersect(intersect(a, b), d), c)

# ACD <- setdiff(intersect(intersect(a, d), c), b)

# BCD <- setdiff(intersect(intersect(d, b), c), a)

# ABCD <- intersect(intersect(intersect(a, b), c), d)

# items <- list(A = A, B = B, C = C, D = D, AB = AB, AC = AC,

# AD = AD, BC = BC, BD = BD, CD = CD, ABC = ABC, ABD = ABD,

# ACD = ACD, BCD = BCD, ABCD = ABCD)

## 5 group ##

# A <- setdiff(a, Reduce(union, list(b, c, d, e)))

# B <- setdiff(b, Reduce(union, list(a, c, d, e)))

# C <- setdiff(c, Reduce(union, list(a, b, d, e)))

# D <- setdiff(d, Reduce(union, list(a, b, c, e)))

# E <- setdiff(e, Reduce(union, list(a, b, c, d)))

#

# AB <- setdiff(intersect(a, b), Reduce(union, list(c, d, e)))

# AC <- setdiff(intersect(a, c), Reduce(union, list(b, d, e)))

# AD <- setdiff(intersect(a, d), Reduce(union, list(b, c, e)))

# AE <- setdiff(intersect(a, e), Reduce(union, list(b, c, d)))

# BC <- setdiff(intersect(b, c), Reduce(union, list(a, d, e)))

# BD <- setdiff(intersect(b, d), Reduce(union, list(a, c, e)))

# BE <- setdiff(intersect(b, e), Reduce(union, list(a, c, d)))

# CD <- setdiff(intersect(c, d), Reduce(union, list(a, b, e)))

# CE <- setdiff(intersect(c, e), Reduce(union, list(a, b, d)))

# DE <- setdiff(intersect(d, e), Reduce(union, list(a, b, c)))

#

# ABC <- setdiff(Reduce(intersect, list(a, b, c)), union(d, e))

# ABD <- setdiff(Reduce(intersect, list(a, b, d)), union(c, e))

# ABE <- setdiff(Reduce(intersect, list(a, b, e)), union(c, d))

# ACD <- setdiff(Reduce(intersect, list(a, c, d)), union(b, e))

# ACE <- setdiff(Reduce(intersect, list(a, c, e)), union(b, d))

# ADE <- setdiff(Reduce(intersect, list(a, d, e)), union(b, c))

# BCD <- setdiff(Reduce(intersect, list(b, c, d)), union(a, e))

# BCE <- setdiff(Reduce(intersect, list(b, c, e)), union(a, d))

# BDE <- setdiff(Reduce(intersect, list(b, d, e)), union(a, c))

# CDE <- setdiff(Reduce(intersect, list(c, d, e)), union(a, b))

#

# ABCD <- setdiff(Reduce(intersect, list(a, b, c, d)), e)

# ABCE <- setdiff(Reduce(intersect, list(a, b, c, e)), d)

# ABDE <- setdiff(Reduce(intersect, list(a, b, d, e)), c)

# ACDE <- setdiff(Reduce(intersect, list(a, c, d, e)), b)

# BCDE <- setdiff(Reduce(intersect, list(b, c, d, e)), a)

#

# ABCDE <- Reduce(intersect, list(a, b, c, d, e))

#

# items <- list(A = A, B = B, C = C, D = D, E = E,

# AB = AB, AC = AC, AD = AD, AE = AE,

# BC = BC, BD = BD, BE = BE,

# CD = CD, CE = CE, DE = DE,

# ABC = ABC, ABD = ABD, ABE = ABE, ACD = ACD, ACE = ACE, ADE = ADE,

# BCD = BCD, BCE = BCE, BDE = BDE, CDE = CDE,

# ABCD = ABCD, ABCE = ABCE, ABDE = ABDE,

# ACDE = ACDE, BCDE = BCDE,

# ABCDE = ABCDE)

library(VennDiagram)

venn.diagram(

x = items, filename = "veen.png",

imagetype="png" ,

height = 480,

width = 480,

resolution = 300

)

## the version of ggplot2 is not provided

**B：**

# library(tidyverse)

library(survival)

library(glmnet)

library(readxl)

## read data

data <- read_xlsx("~/file.xlsx")

head(data)

# event time Gene 1 Gene 2 Gene 3 Gene 4 Gene 5 Gene 6 Gene 7 Gene 8 Gene 9 Gene 10 Gene 11

# 1 1 306 -0.02228062 -2.7704356 -0.4670543 0.6597100 -0.5151171 0.02662303 0.9648682 -1.0649103 0.1261321 -1.2472454 0.2857966

# 2 1 455 -1.18321709 -0.3161180 0.6060377 -0.3035985 0.1487279 -1.21013808 1.2133766 -0.1951160 0.6075327 0.3840250 0.6994784

# 3 0 1010 -0.62297439 1.8458862 1.5176689 -0.8392173 -0.2736995 -1.91479449 0.9869319 -0.1859204 0.2624222 0.8615537 -0.5557269

# 4 1 210 -0.96143221 -0.1361290 0.7070270 -2.2407777 -0.1158965 -1.67839318 0.5813433 1.2488396 -0.4216805 -0.3247411 -0.1496818

# 5 1 883 -2.00905791 0.7544725 -1.3601112 0.7434566 1.2420130 0.37301567 0.6557969 -0.6545812 -0.7919030 -0.7085475 -2.8072731

# 6 0 1022 0.79356585 -0.2366209 -0.5012338 0.9380560 -1.2196590 -1.62508198 0.3280815 1.0461292 -0.6751357 1.4510194 -0.7491115

set.seed(2021)

cvfit = cv.glmnet(x = as.matrix(data[,c(-1, -2)]),

y = Surv(time = data$time, event = data$event), family = "cox",

alpha = 1)

# cvfit

# Call: cv.glmnet(x = as.matrix(data[, c(-1, -2)]), y = Surv(time = data$time, event = data$event), family = "cox", alpha = 1)

#

# Measure: Partial Likelihood Deviance

#

# Lambda Index Measure SE Nonzero

# min 0.09095 7 10.16 0.1983 4

# 1se 0.15894 1 10.17 0.1893 0

## Check Coefficients

# coef(cvfit, s = "lambda.1se")

coef.min = coef(cvfit, s = "lambda.min")

## riskscore

as.matrix(dat1[,c(-1,-2)]) %*% as.matrix(coef.min)

## plot

plot(cvfit)

plot(cvfit$glmnet.fit, xvar = "norm")

plot(cvfit$glmnet.fit, xvar = "lambda")

**C：**

# library(tidyverse)

library(survival)

library(glmnet)

library(readxl)

## read data

data <- read_xlsx("~/file.xlsx")

head(data)

# event time Gene 1 Gene 2 Gene 3 Gene 4 Gene 5 Gene 6 Gene 7 Gene 8 Gene 9 Gene 10 Gene 11

# 1 1 306 -0.02228062 -2.7704356 -0.4670543 0.6597100 -0.5151171 0.02662303 0.9648682 -1.0649103 0.1261321 -1.2472454 0.2857966

# 2 1 455 -1.18321709 -0.3161180 0.6060377 -0.3035985 0.1487279 -1.21013808 1.2133766 -0.1951160 0.6075327 0.3840250 0.6994784

# 3 0 1010 -0.62297439 1.8458862 1.5176689 -0.8392173 -0.2736995 -1.91479449 0.9869319 -0.1859204 0.2624222 0.8615537 -0.5557269

# 4 1 210 -0.96143221 -0.1361290 0.7070270 -2.2407777 -0.1158965 -1.67839318 0.5813433 1.2488396 -0.4216805 -0.3247411 -0.1496818

# 5 1 883 -2.00905791 0.7544725 -1.3601112 0.7434566 1.2420130 0.37301567 0.6557969 -0.6545812 -0.7919030 -0.7085475 -2.8072731

# 6 0 1022 0.79356585 -0.2366209 -0.5012338 0.9380560 -1.2196590 -1.62508198 0.3280815 1.0461292 -0.6751357 1.4510194 -0.7491115

set.seed(2021)

cvfit = cv.glmnet(x = as.matrix(data[,c(-1, -2)]),

y = Surv(time = data$time, event = data$event), family = "cox",

alpha = 1)

# cvfit

# Call: cv.glmnet(x = as.matrix(data[, c(-1, -2)]), y = Surv(time = data$time, event = data$event), family = "cox", alpha = 1)

#

# Measure: Partial Likelihood Deviance

#

# Lambda Index Measure SE Nonzero

# min 0.09095 7 10.16 0.1983 4

# 1se 0.15894 1 10.17 0.1893 0

## Check Coefficients

# coef(cvfit, s = "lambda.1se")

coef.min = coef(cvfit, s = "lambda.min")

## riskscore

as.matrix(dat1[,c(-1,-2)]) %*% as.matrix(coef.min)

## plot

plot(cvfit)

plot(cvfit$glmnet.fit, xvar = "norm")

plot(cvfit$glmnet.fit, xvar = "lambda")

1. **E：**

f (!requireNamespace("survminer", quietly = TRUE))

install.packages("survminer")

library(survival)

library(survminer)

# data <- lung

# colnames(data)[5] <- "variable"

fit <- survfit(Surv(time, status) ~ variable, data = data)

print(fit)

# Call: survfit(formula = Surv(time, status) ~ variable, data = data)

#

# n events median 0.95LCL 0.95UCL

# variable=1 138 112 270 212 310

# variable=2 90 53 426 348 550

survdiff(Surv(time, status) ~ variable, data = data)

# survdiff(formula = Surv(time, status) ~ variable, data = data)

#

# N Observed Expected (O-E)^2/E (O-E)^2/V

# variable=1 138 112 91.6 4.55 10.3

# variable=2 90 53 73.4 5.68 10.3

#

# Chisq= 10.3 on 1 degrees of freedom, p= 0.001

fit2 <- coxph(Surv(time, status) ~ variable, data = data)

summary(fit2)

# Call:

# coxph(formula = Surv(time, status) ~ variable, data = data)

#

# n= 228, number of events= 165

#

# coef exp(coef) se(coef) z Pr(>|z|)

# variable -0.5310 0.5880 0.1672 -3.176 0.00149 **

# ---

# Signif. codes: 0 ‘***’ 0.001 ‘**’ 0.01 ‘*’ 0.05 ‘.’ 0.1 ‘ ’ 1

#

# exp(coef) exp(-coef) lower .95 upper .95

# variable 0.588 1.701 0.4237 0.816

#

# Concordance= 0.579 (se = 0.021 )

# Likelihood ratio test= 10.63 on 1 df, p=0.001

# Wald test = 10.09 on 1 df, p=0.001

# Score (logrank) test = 10.33 on 1 df, p=0.001

# plot

ggsurvplot(fit = fit, data = data, pval = T)

library(tidyverse)

library(ggplot2)

library(timeROC)

data <- dat

data <- read.table("~/file.txt", header = T)

fit_timeROC <-

timeROC(`T` = data$time,

delta = data$event,

marker = data$group,

cause = 1, weighting="marginal",

times = c(1*365, 3*365, 5*365), iid = T)

fit_timeROC

# Time-dependent-Roc curve estimated using IPCW (n=418, without competing risks).

# Cases Survivors Censored AUC (%) se

# t=365 30 388 0 82.30 3.64

# t=1095 81 312 25 83.42 2.48

# t=1825 115 197 106 86.23 2.15

#

# Method used for estimating IPCW:marginal

#

# Total computation time : 0.24 secs.

confint(fit_timeROC)

# $CI_AUC

# 2.5% 97.5%

# t=365 75.16 89.44

# t=1095 78.55 88.28

# t=1825 82.02 90.44

#

# $CB_AUC

# 2.5% 97.5%

# t=365 73.97 90.63

# t=1095 77.74 89.09

# t=1825 81.32 91.14

#

# $C.alpha

# 95%

# 2.286114

cut_value = sort(unique(data$group))

tp = rev(fit_timeROC$TP[,1])

fp = rev(fit_timeROC$FP[,1])

idx <- which.max(tp - fp)[1]

SeSpPPVNPV(cutpoint=cut_value[idx - 1],

`T` = data$time,

delta = data$event,

marker = data$group,

cause= 1,

weighting="marginal",

times = c(1*365))

# Predictive accuracy measures at cutpoint c=2.3 estimated using IPCW (n=418, with competing risks).

# No. of positive (X>c) =143, No. of negative (X<=c) =275.

#

# Cases Survivors Censored Se (%) Sp (%) PPV (%) NPV (%)

# t=0 0 418 0 NA 65.79 NA 100.00

# t=365 30 388 0 86.67 69.85 18.18 98.55

#

# Method used for estimating IPCW:marginal

#

# Total computation time : 0 secs.

data2 <- data.frame(group = "1-Year",

x = fit_timeROC$FP[,1],

y = fit_timeROC$TP[,1])

data2 <- data2[order(data2$x, data2$y),]

tmp <- data.frame(group = "3-Year",

x = fit_timeROC$FP[,2],

y = fit_timeROC$TP[,2])

tmp <- tmp[order(tmp$x, tmp$y),]

data2 <- rbind(data2, tmp)

ggplot() +

geom_line(data = data2, aes(x = x, y = y, colour = group)) +

labs(x = "1-Specificity (FPR)", y = "Sensitivity (TPR)")

1. Figure 2 Related R packages:
2. **B：**

library(tidyverse)

library(survival)

library(readxl)

## read data

data <- read_xlsx("~/file.xlsx")

## tidy data

# data$event <- as.numeric(data$event)

# data$time <- as.numeric(data$time)

### numeric

data$Age <- as.numeric(data$Age)

data$Score <- as.numeric(data$Score)

### factor

data$Sex <- factor(data$Sex, levels = c("Male", "Female"))

data$Grade <- factor(data$Grade, levels = c("0", "1", "2"))

data$Stage <- factor(data$Stage, levels = c("Stage1", "Stage2", "Stage3", "Stage4"))

## summary

fit <- survfit(Surv(time, event) ~ Sex, data = data)

fit

# Call: survfit(formula = Surv(time, event) ~ Sex, data = data)

#

# n events median 0.95LCL 0.95UCL

# Sex=Female 90 53 426 348 550

# Sex=Male 138 112 270 212 310

## univariable Cox

fit <- coxph(Surv(time, event) ~ Sex, data = data)

summary(fit)

# Call:

# coxph(formula = Surv(time, event) ~ Sex, data = data)

#

# n= 228, number of events= 165

#

# coef exp(coef) se(coef) z Pr(>|z|)

# SexMale 0.5310 1.7007 0.1672 3.176 0.00149 **

# ---

# Signif. codes: 0 ‘***’ 0.001 ‘**’ 0.01 ‘*’ 0.05 ‘.’ 0.1 ‘ ’ 1

#

# exp(coef) exp(-coef) lower .95 upper .95

# SexMale 1.701 0.588 1.226 2.36

#

# Concordance= 0.579 (se = 0.021 )

# Likelihood ratio test= 10.63 on 1 df, p=0.001

# Wald test = 10.09 on 1 df, p=0.001

# Score (logrank) test = 10.33 on 1 df, p=0.001

fit <- coxph(Surv(time, event) ~ Age, data = data)

summary(fit)

# Call:

# coxph(formula = Surv(time, event) ~ Age, data = data)

#

# n= 228, number of events= 165

#

# coef exp(coef) se(coef) z Pr(>|z|)

# Age 0.019543 1.019735 0.008735 2.237 0.0253 *

# ---

# Signif. codes: 0 ‘***’ 0.001 ‘**’ 0.01 ‘*’ 0.05 ‘.’ 0.1 ‘ ’ 1

#

# exp(coef) exp(-coef) lower .95 upper .95

# Age 1.02 0.9806 1.002 1.037

#

# Concordance= 0.55 (se = 0.025 )

# Likelihood ratio test= 5.13 on 1 df, p=0.02

# Wald test = 5.01 on 1 df, p=0.03

# Score (logrank) test = 5.02 on 1 df, p=0.03

## multivariable Cox

fit <- coxph(Surv(time, event) ~ Age + Sex + Stage + Score, data = data)

summary(fit)

# Call:

# coxph(formula = Surv(time, event) ~ Age + Sex + Stage + Score,

# data = data)

#

# n= 224, number of events= 161

# (4 observations deleted due to missingness)

#

# coef exp(coef) se(coef) z Pr(>|z|)

# Age 0.010922 1.010982 0.008972 1.217 0.2235

# SexMale 0.536001 1.709159 0.169829 3.156 0.0016 **

# StageStage2 0.390113 1.477148 0.204289 1.910 0.0562 .

# StageStage3 0.693144 1.999993 0.283818 2.442 0.0146 *

# StageStage4 1.830114 6.234597 1.035948 1.767 0.0773 .

# Score -0.009346 0.990698 0.007127 -1.311 0.1897

# ---

# Signif. codes: 0 ‘***’ 0.001 ‘**’ 0.01 ‘*’ 0.05 ‘.’ 0.1 ‘ ’ 1

#

# exp(coef) exp(-coef) lower .95 upper .95

# Age 1.0110 0.9891 0.9934 1.029

# SexMale 1.7092 0.5851 1.2252 2.384

# StageStage2 1.4771 0.6770 0.9898 2.205

# StageStage3 2.0000 0.5000 1.1467 3.488

# StageStage4 6.2346 0.1604 0.8185 47.491

# Score 0.9907 1.0094 0.9770 1.005

#

# Concordance= 0.654 (se = 0.025 )

# Likelihood ratio test= 32.28 on 6 df, p=1e-05

# Wald test = 32.66 on 6 df, p=1e-05

# Score (logrank) test = 34.96 on 6 df, p=4e-06

**C、D：**

library(tidyverse)

library(survival)

library(readxl)

library(rms)

## read data

data <- read_xlsx("~/file.xlsx")

## tidy data

# data$event <- as.numeric(data$event)

# data$time <- as.numeric(data$time)

### numeric

data$Age <- as.numeric(data$Age)

data$Score <- as.numeric(data$Score)

### factor

data$Sex <- factor(data$Sex, levels = c("Male", "Female"))

data$Grade <- factor(data$Grade, levels = c("0", "1", "2"))

data$Stage <- factor(data$Stage, levels = c("Stage1", "Stage2", "Stage3", "Stage4"))

colnames(data)[4] <- "Weight_loss"

## summary

fit <- survfit(Surv(time, event) ~ Sex, data = data)

fit

# Call: survfit(formula = Surv(time, event) ~ Sex, data = data)

#

# n events median 0.95LCL 0.95UCL

# Sex=Male 138 112 270 212 310

# Sex=Female 90 53 426 348 550

fit <- coxph(Surv(time = time, event = event) ~ Age + Weight_loss + Sex + Grade + Stage + Score, data = data)

summary(fit)

# Call:

# coxph(formula = Surv(time = time, event = event) ~ Age + Weight_loss +

# Sex + Grade + Stage + Score, data = data)

#

# n= 210, number of events= 148

# (18 observations deleted due to missingness)

#

# coef exp(coef) se(coef) z Pr(>|z|)

# Age 0.012963 1.013047 0.009363 1.384 0.16622

# Weight_loss -0.013179 0.986908 0.007049 -1.870 0.06153 .

# SexFemale -0.648482 0.522839 0.181763 -3.568 0.00036 ***

# Grade1 0.247066 1.280264 0.215206 1.148 0.25095

# Grade2 -0.167493 0.845782 0.243006 -0.689 0.49066

# StageStage2 0.447155 1.563856 0.211797 2.111 0.03475 *

# StageStage3 0.791586 2.206894 0.299925 2.639 0.00831 **

# StageStage4 2.083022 8.028691 1.056227 1.972 0.04859 *

# Score -0.013471 0.986619 0.007642 -1.763 0.07795 .

# ---

# Signif. codes: 0 ‘***’ 0.001 ‘**’ 0.01 ‘*’ 0.05 ‘.’ 0.1 ‘ ’ 1

#

# exp(coef) exp(-coef) lower .95 upper .95

# Age 1.0130 0.9871 0.9946 1.0318

# Weight_loss 0.9869 1.0133 0.9734 1.0006

# SexFemale 0.5228 1.9126 0.3661 0.7466

# Grade1 1.2803 0.7811 0.8397 1.9520

# Grade2 0.8458 1.1823 0.5253 1.3618

# StageStage2 1.5639 0.6394 1.0326 2.3685

# StageStage3 2.2069 0.4531 1.2260 3.9726

# StageStage4 8.0287 0.1246 1.0129 63.6366

# Score 0.9866 1.0136 0.9720 1.0015

#

# Concordance= 0.665 (se = 0.026 )

# Likelihood ratio test= 38.77 on 9 df, p=1e-05

# Wald test = 36.84 on 9 df, p=3e-05

# Score (logrank) test = 39.94 on 9 df, p=8e-06

dat1 = datadist(data)

options(datadist = "dat1")

cph1 <- cph(formula = Surv(time = time, event = event) ~ Age + Weight_loss + Sex + Grade + Stage + Score,

data=data, x=T, y=T, surv = T)

surv <- Survival(cph1)

surv1 <- function(x) surv(365*1,x)

surv2 <- function(x) surv(365*2,x)

fit <- nomogram(cph1, fun = list(surv1, surv2), lp=T,

funlabel = c("1-Year", "2-Year"))

plot(fit)

1. Figure 3 Related R packages:

**A：**

library(ggplot2)

library(reshape2)

set.seed(100)

data <- data.frame(x = rnorm(100, 2, 1), y = rnorm(100, 1, 1))

data$id <- 1:nrow(data)

data2 <- melt(data, id.vars = "id")

lapply(data, function(x) get_summary_stats(data.frame(x)))

# $x

# # A tibble: 1 x 13

# variable n min max median q1 q3 iqr mad mean sd se ci

# <chr> <dbl> <dbl> <dbl> <dbl> <dbl> <dbl> <dbl> <dbl> <dbl> <dbl> <dbl> <dbl>

# 1 x 100 -0.272 4.58 1.94 1.39 2.66 1.26 0.974 2.00 1.02 0.102 0.203

#

# $y

# # A tibble: 1 x 13

# variable n min max median q1 q3 iqr mad mean sd se ci

# <chr> <dbl> <dbl> <dbl> <dbl> <dbl> <dbl> <dbl> <dbl> <dbl> <dbl> <dbl> <dbl>

# 1 x 100 -1.14 3.17 0.927 0.568 1.45 0.878 0.648 1.01 0.796 0.08 0.158

data$diff <- data$x - data$y

shapiro.test(data$diff)

# Shapiro-Wilk normality test

#

# data: data$diff

# W = 0.98792, p-value = 0.5024

t.test(data$x, data$y, paired = T)

# Paired t-test

#

# data: data$x and data$y

# t = 7.2039, df = 99, p-value = 1.165e-10

# alternative hypothesis: true difference in means is not equal to 0

# 95 percent confidence interval:

# 0.7186033 1.2649401

# sample estimates:

# mean of the differences

# 0.9917717

wilcox.test(data$x, data$y, paired = T)

# Wilcoxon signed rank test with continuity correction

#

# data: data$x and data$y

# V = 4287, p-value = 1.39e-09

# alternative hypothesis: true location shift is not equal to 0

ggplot(data = data2, aes(x = variable, y = value)) +

geom_line(aes(group = id))+

geom_point(aes(color = variable))

**B:**

library(tidyverse)

library(ggplot2)

library(reshape2)

library(car)

library(rstatix)

set.seed(100)

data <- data.frame(x = rnorm(100, 2, 1), y = rnorm(100, 1, 1))

data2 <- melt(data)

data3 <- lapply(data, function(x) get_summary_stats(data.frame(x)))

data3

# $x

# # A tibble: 1 x 13

# variable n min max median q1 q3 iqr mad mean sd se ci

# <chr> <dbl> <dbl> <dbl> <dbl> <dbl> <dbl> <dbl> <dbl> <dbl> <dbl> <dbl> <dbl>

# 1 x 100 -0.272 4.58 1.94 1.39 2.66 1.26 0.974 2.00 1.02 0.102 0.203

#

# $y

# # A tibble: 1 x 13

# variable n min max median q1 q3 iqr mad mean sd se ci

# <chr> <dbl> <dbl> <dbl> <dbl> <dbl> <dbl> <dbl> <dbl> <dbl> <dbl> <dbl> <dbl>

# 1 x 100 -1.14 3.17 0.927 0.568 1.45 0.878 0.648 1.01 0.796 0.08 0.158

data3 <- rbind(data3[[1]], data3[[2]])

data3[1] <- c("x", "y")

## Shapiro-Wilk normality test

lapply(data, function(x) shapiro.test(x))

# $x

#

# Shapiro-Wilk normality test

#

# data: x

# W = 0.98836, p-value = 0.535

#

#

# $y

#

# Shapiro-Wilk normality test

#

# data: x

# W = 0.98532, p-value = 0.3348

## Levene's Test

leveneTest(value~variable, data = data2)

# Levene's Test for Homogeneity of Variance (center = median)

# Df F value Pr(>F)

# group 1 4.4476 0.03621 *

# 198

# ---

# Signif. codes: 0 ‘***’ 0.001 ‘**’ 0.01 ‘*’ 0.05 ‘.’ 0.1 ‘ ’ 1

t.test(value~variable, data = data2, var.equal = T)

# Two Sample t-test

#

# data: value by variable

# t = 7.6613, df = 198, p-value = 8.012e-13

# alternative hypothesis: true difference in means is not equal to 0

# 95 percent confidence interval:

# 0.7364913 1.2470521

# sample estimates:

# mean in group x mean in group y

# 2.002913 1.011141

t.test(value~variable, data = data2, var.equal = F)

# Welch Two Sample t-test

#

# data: value by variable

# t = 7.6613, df = 186.92, p-value = 9.657e-13

# alternative hypothesis: true difference in means is not equal to 0

# 95 percent confidence interval:

# 0.7363983 1.2471452

# sample estimates:

# mean in group x mean in group y

# 2.002913 1.011141

wilcox.test(value~variable, data = data2)

# Wilcoxon rank sum test with continuity correction

#

# data: value by variable

# W = 7844, p-value = 3.711e-12

# alternative hypothesis: true location shift is not equal to 0

summary(aov(value~variable, data = data2))

# Df Sum Sq Mean Sq F value Pr(>F)

# variable 1 49.18 49.18 58.7 8.01e-13 ***

# Residuals 198 165.90 0.84

# ---

# Signif. codes: 0 ‘***’ 0.001 ‘**’ 0.01 ‘*’ 0.05 ‘.’ 0.1 ‘ ’ 1

ggplot(data2, aes(x = variable, y = value, color = variable, fill = variable)) +

geom_violin(alpha = 0.2) +

theme_bw()

ggplot(data2, aes(x = variable, y = value, color = variable, fill = variable)) +

geom_violin(alpha = 0.2) +

geom_point(position = position_jitter(0.3)) +

theme_bw()

ggplot(data2, aes(x = variable, y = value, color = variable, fill = variable)) +

geom_boxplot(alpha = 0.2) +

geom_point(position = position_jitter(0.3)) +

theme_bw()

ggplot(data2, aes(x = variable, y = value, color = variable, fill = variable)) +

geom_violin(alpha = 0.1) +

geom_boxplot(alpha = 0.1) +

geom_point(position = position_jitter(0.3)) +

theme_bw()

ggplot() +

geom_violin(data = data2, aes(x = variable, y = value, color = variable, fill = variable), alpha = 0.1) +

geom_errorbar(data = data3, aes(x = variable, ymin=mean-sd, ymax=mean+sd), width = 0.2)

**C、D：**

library(tidyverse)

library(ggplot2)

library(reshape2)

library(car)

library(rstatix)

set.seed(100)

data <- data.frame(x = rnorm(100, 2, 1), y = rnorm(100, 1, 1))

data2 <- melt(data)

data3 <- lapply(data, function(x) get_summary_stats(data.frame(x)))

data3

# $x

# # A tibble: 1 x 13

# variable n min max median q1 q3 iqr mad mean sd se ci

# <chr> <dbl> <dbl> <dbl> <dbl> <dbl> <dbl> <dbl> <dbl> <dbl> <dbl> <dbl> <dbl>

# 1 x 100 -0.272 4.58 1.94 1.39 2.66 1.26 0.974 2.00 1.02 0.102 0.203

#

# $y

# # A tibble: 1 x 13

# variable n min max median q1 q3 iqr mad mean sd se ci

# <chr> <dbl> <dbl> <dbl> <dbl> <dbl> <dbl> <dbl> <dbl> <dbl> <dbl> <dbl> <dbl>

# 1 x 100 -1.14 3.17 0.927 0.568 1.45 0.878 0.648 1.01 0.796 0.08 0.158

data3 <- rbind(data3[[1]], data3[[2]])

data3[1] <- c("x", "y")

## Shapiro-Wilk normality test

lapply(data, function(x) shapiro.test(x))

# $x

#

# Shapiro-Wilk normality test

#

# data: x

# W = 0.98836, p-value = 0.535

#

#

# $y

#

# Shapiro-Wilk normality test

#

# data: x

# W = 0.98532, p-value = 0.3348

## Levene's Test

leveneTest(value~variable, data = data2)

# Levene's Test for Homogeneity of Variance (center = median)

# Df F value Pr(>F)

# group 1 4.4476 0.03621 *

# 198

# ---

# Signif. codes: 0 ‘***’ 0.001 ‘**’ 0.01 ‘*’ 0.05 ‘.’ 0.1 ‘ ’ 1

t.test(value~variable, data = data2, var.equal = T)

# Two Sample t-test

#

# data: value by variable

# t = 7.6613, df = 198, p-value = 8.012e-13

# alternative hypothesis: true difference in means is not equal to 0

# 95 percent confidence interval:

# 0.7364913 1.2470521

# sample estimates:

# mean in group x mean in group y

# 2.002913 1.011141

t.test(value~variable, data = data2, var.equal = F)

# Welch Two Sample t-test

#

# data: value by variable

# t = 7.6613, df = 186.92, p-value = 9.657e-13

# alternative hypothesis: true difference in means is not equal to 0

# 95 percent confidence interval:

# 0.7363983 1.2471452

# sample estimates:

# mean in group x mean in group y

# 2.002913 1.011141

wilcox.test(value~variable, data = data2)

# Wilcoxon rank sum test with continuity correction

#

# data: value by variable

# W = 7844, p-value = 3.711e-12

# alternative hypothesis: true location shift is not equal to 0

summary(aov(value~variable, data = data2))

# Df Sum Sq Mean Sq F value Pr(>F)

# variable 1 49.18 49.18 58.7 8.01e-13 ***

# Residuals 198 165.90 0.84

# ---

# Signif. codes: 0 ‘***’ 0.001 ‘**’ 0.01 ‘*’ 0.05 ‘.’ 0.1 ‘ ’ 1

ggplot(data2, aes(x = variable, y = value, color = variable, fill = variable)) +

geom_violin(alpha = 0.2) +

theme_bw()

ggplot(data2, aes(x = variable, y = value, color = variable, fill = variable)) +

geom_violin(alpha = 0.2) +

geom_point(position = position_jitter(0.3)) +

theme_bw()

ggplot(data2, aes(x = variable, y = value, color = variable, fill = variable)) +

geom_boxplot(alpha = 0.2) +

geom_point(position = position_jitter(0.3)) +

theme_bw()

ggplot(data2, aes(x = variable, y = value, color = variable, fill = variable)) +

geom_violin(alpha = 0.1) +

geom_boxplot(alpha = 0.1) +

geom_point(position = position_jitter(0.3)) +

theme_bw()

ggplot() +

geom_violin(data = data2, aes(x = variable, y = value, color = variable, fill = variable), alpha = 0.1) +

geom_errorbar(data = data3, aes(x = variable, ymin=mean-sd, ymax=mean+sd), width = 0.2)

**F：**

library(ggplot2)

library(reshape2)

set.seed(100)

data <- data.frame(x = rnorm(100, 2, 1), y = rnorm(100, 1, 1))

data$id <- 1:nrow(data)

data2 <- melt(data, id.vars = "id")

lapply(data, function(x) get_summary_stats(data.frame(x)))

# $x

# # A tibble: 1 x 13

# variable n min max median q1 q3 iqr mad mean sd se ci

# <chr> <dbl> <dbl> <dbl> <dbl> <dbl> <dbl> <dbl> <dbl> <dbl> <dbl> <dbl> <dbl>

# 1 x 100 -0.272 4.58 1.94 1.39 2.66 1.26 0.974 2.00 1.02 0.102 0.203

#

# $y

# # A tibble: 1 x 13

# variable n min max median q1 q3 iqr mad mean sd se ci

# <chr> <dbl> <dbl> <dbl> <dbl> <dbl> <dbl> <dbl> <dbl> <dbl> <dbl> <dbl> <dbl>

# 1 x 100 -1.14 3.17 0.927 0.568 1.45 0.878 0.648 1.01 0.796 0.08 0.158

data$diff <- data$x - data$y

shapiro.test(data$diff)

# Shapiro-Wilk normality test

#

# data: data$diff

# W = 0.98792, p-value = 0.5024

t.test(data$x, data$y, paired = T)

# Paired t-test

#

# data: data$x and data$y

# t = 7.2039, df = 99, p-value = 1.165e-10

# alternative hypothesis: true difference in means is not equal to 0

# 95 percent confidence interval:

# 0.7186033 1.2649401

# sample estimates:

# mean of the differences

# 0.9917717

wilcox.test(data$x, data$y, paired = T)

# Wilcoxon signed rank test with continuity correction

#

# data: data$x and data$y

# V = 4287, p-value = 1.39e-09

# alternative hypothesis: true location shift is not equal to 0

ggplot(data = data2, aes(x = variable, y = value)) +

geom_line(aes(group = id))+

geom_point(aes(color = variable))

1. Figure 5 Related R packages:

library(tidyverse)

library(ggplot2)

library(reshape2)

library(car)

library(rstatix)

set.seed(100)

data <- data.frame(x = rnorm(100, 2, 1), y = rnorm(100, 1, 1))

data2 <- melt(data)

data3 <- lapply(data, function(x) get_summary_stats(data.frame(x)))

data3

# $x

# # A tibble: 1 x 13

# variable n min max median q1 q3 iqr mad mean sd se ci

# <chr> <dbl> <dbl> <dbl> <dbl> <dbl> <dbl> <dbl> <dbl> <dbl> <dbl> <dbl> <dbl>

# 1 x 100 -0.272 4.58 1.94 1.39 2.66 1.26 0.974 2.00 1.02 0.102 0.203

#

# $y

# # A tibble: 1 x 13

# variable n min max median q1 q3 iqr mad mean sd se ci

# <chr> <dbl> <dbl> <dbl> <dbl> <dbl> <dbl> <dbl> <dbl> <dbl> <dbl> <dbl> <dbl>

# 1 x 100 -1.14 3.17 0.927 0.568 1.45 0.878 0.648 1.01 0.796 0.08 0.158

data3 <- rbind(data3[[1]], data3[[2]])

data3[1] <- c("x", "y")

## Shapiro-Wilk normality test

lapply(data, function(x) shapiro.test(x))

# $x

#

# Shapiro-Wilk normality test

#

# data: x

# W = 0.98836, p-value = 0.535

#

#

# $y

#

# Shapiro-Wilk normality test

#

# data: x

# W = 0.98532, p-value = 0.3348

## Levene's Test

leveneTest(value~variable, data = data2)

# Levene's Test for Homogeneity of Variance (center = median)

# Df F value Pr(>F)

# group 1 4.4476 0.03621 *

# 198

# ---

# Signif. codes: 0 ‘***’ 0.001 ‘**’ 0.01 ‘*’ 0.05 ‘.’ 0.1 ‘ ’ 1

t.test(value~variable, data = data2, var.equal = T)

# Two Sample t-test

#

# data: value by variable

# t = 7.6613, df = 198, p-value = 8.012e-13

# alternative hypothesis: true difference in means is not equal to 0

# 95 percent confidence interval:

# 0.7364913 1.2470521

# sample estimates:

# mean in group x mean in group y

# 2.002913 1.011141

t.test(value~variable, data = data2, var.equal = F)

# Welch Two Sample t-test

#

# data: value by variable

# t = 7.6613, df = 186.92, p-value = 9.657e-13

# alternative hypothesis: true difference in means is not equal to 0

# 95 percent confidence interval:

# 0.7363983 1.2471452

# sample estimates:

# mean in group x mean in group y

# 2.002913 1.011141

wilcox.test(value~variable, data = data2)

# Wilcoxon rank sum test with continuity correction

#

# data: value by variable

# W = 7844, p-value = 3.711e-12

# alternative hypothesis: true location shift is not equal to 0

summary(aov(value~variable, data = data2))

# Df Sum Sq Mean Sq F value Pr(>F)

# variable 1 49.18 49.18 58.7 8.01e-13 ***

# Residuals 198 165.90 0.84

# ---

# Signif. codes: 0 ‘***’ 0.001 ‘**’ 0.01 ‘*’ 0.05 ‘.’ 0.1 ‘ ’ 1

ggplot(data2, aes(x = variable, y = value, color = variable, fill = variable)) +

geom_violin(alpha = 0.2) +

theme_bw()

ggplot(data2, aes(x = variable, y = value, color = variable, fill = variable)) +

geom_violin(alpha = 0.2) +

geom_point(position = position_jitter(0.3)) +

theme_bw()

ggplot(data2, aes(x = variable, y = value, color = variable, fill = variable)) +

geom_boxplot(alpha = 0.2) +

geom_point(position = position_jitter(0.3)) +

theme_bw()

ggplot(data2, aes(x = variable, y = value, color = variable, fill = variable)) +

geom_violin(alpha = 0.1) +

geom_boxplot(alpha = 0.1) +

geom_point(position = position_jitter(0.3)) +

theme_bw()

ggplot() +

geom_violin(data = data2, aes(x = variable, y = value, color = variable, fill = variable), alpha = 0.1) +

geom_errorbar(data = data3, aes(x = variable, ymin=mean-sd, ymax=mean+sd), width = 0.2)

1. Figure 6 Related R packages:

library(tidyverse)

library(ggplot2)

library(patchwork)

library(reshape2)

data <- read.table("~/file.txt", header = T)

head(data)

# target NAT1 ADH1B BIRC5 AQP9 BCL2A1

# 1 6.395299 2.332422 1.7707953 5.002700 1.4738576 3.233794

# 2 12.270018 2.420924 0.9751920 5.623562 0.8144824 1.188484

# 3 6.297991 3.095432 0.1420562 4.625413 0.7765188 0.445318

# 4 6.954923 2.150208 2.1391032 4.816844 1.3144263 2.868153

# 5 6.317630 2.442149 0.1408322 6.183782 2.5177065 5.370463

# 6 5.479180 2.791275 0.7515575 5.991042 3.5884496 5.269202

data$group <- ifelse(data$target >= median(data$target), "High", "Low")

data$group <- factor(data$group, levels = c("Low", "High"))

data<- data[order(data$target), ]

data$id <- 1:nrow(data)

### plot

p1 <- ggplot() +

geom_bar(data = data,

aes(x = id, y = target, color=group, fill = group),

stat = 'identity', position = 'dodge') +

scale_y_continuous(expand = c(0,0)) +

theme_classic() +

theme(axis.title.x = element_blank(), axis.text.x = element_blank(),

axis.ticks.x = element_blank(), axis.line.x = element_blank())

data1 <- data %>%

select(-group, -target) %>%

remove_rownames() %>%

column_to_rownames("id") %>%

scale() %>% as.data.frame() %>%

rownames_to_column("id") %>%

melt()

p2 <-

ggplot(data = data1, aes(x = id, y = as.numeric(variable), fill = value)) +

geom_raster() +

scale_fill_gradientn(colors = c("#4DBBD5", "#FFFFFF", "#E64B35")) +

scale_y_continuous(expand = c(0,0), limits = c(0.5,5.5), breaks = 1:5,

labels = levels(data1$variable)) +

theme(axis.title = element_blank(), axis.text.x = element_blank(),

axis.ticks.x = element_blank(), axis.line.x = element_blank())

p1 / p2

1. Figure 7 Related R packages:

**A：**

library(tidyverse)

library(ggplot2)

library(reshape2)

library(car)

library(rstatix)

set.seed(100)

data <- data.frame(x = rnorm(100, 2, 1), y = rnorm(100, 1, 1))

data2 <- melt(data)

data3 <- lapply(data, function(x) get_summary_stats(data.frame(x)))

data3

# $x

# # A tibble: 1 x 13

# variable n min max median q1 q3 iqr mad mean sd se ci

# <chr> <dbl> <dbl> <dbl> <dbl> <dbl> <dbl> <dbl> <dbl> <dbl> <dbl> <dbl> <dbl>

# 1 x 100 -0.272 4.58 1.94 1.39 2.66 1.26 0.974 2.00 1.02 0.102 0.203

#

# $y

# # A tibble: 1 x 13

# variable n min max median q1 q3 iqr mad mean sd se ci

# <chr> <dbl> <dbl> <dbl> <dbl> <dbl> <dbl> <dbl> <dbl> <dbl> <dbl> <dbl> <dbl>

# 1 x 100 -1.14 3.17 0.927 0.568 1.45 0.878 0.648 1.01 0.796 0.08 0.158

data3 <- rbind(data3[[1]], data3[[2]])

data3[1] <- c("x", "y")

## Shapiro-Wilk normality test

lapply(data, function(x) shapiro.test(x))

# $x

#

# Shapiro-Wilk normality test

#

# data: x

# W = 0.98836, p-value = 0.535

#

#

# $y

#

# Shapiro-Wilk normality test

#

# data: x

# W = 0.98532, p-value = 0.3348

## Levene's Test

leveneTest(value~variable, data = data2)

# Levene's Test for Homogeneity of Variance (center = median)

# Df F value Pr(>F)

# group 1 4.4476 0.03621 *

# 198

# ---

# Signif. codes: 0 ‘***’ 0.001 ‘**’ 0.01 ‘*’ 0.05 ‘.’ 0.1 ‘ ’ 1

t.test(value~variable, data = data2, var.equal = T)

# Two Sample t-test

#

# data: value by variable

# t = 7.6613, df = 198, p-value = 8.012e-13

# alternative hypothesis: true difference in means is not equal to 0

# 95 percent confidence interval:

# 0.7364913 1.2470521

# sample estimates:

# mean in group x mean in group y

# 2.002913 1.011141

t.test(value~variable, data = data2, var.equal = F)

# Welch Two Sample t-test

#

# data: value by variable

# t = 7.6613, df = 186.92, p-value = 9.657e-13

# alternative hypothesis: true difference in means is not equal to 0

# 95 percent confidence interval:

# 0.7363983 1.2471452

# sample estimates:

# mean in group x mean in group y

# 2.002913 1.011141

wilcox.test(value~variable, data = data2)

# Wilcoxon rank sum test with continuity correction

#

# data: value by variable

# W = 7844, p-value = 3.711e-12

# alternative hypothesis: true location shift is not equal to 0

summary(aov(value~variable, data = data2))

# Df Sum Sq Mean Sq F value Pr(>F)

# variable 1 49.18 49.18 58.7 8.01e-13 ***

# Residuals 198 165.90 0.84

# ---

# Signif. codes: 0 ‘***’ 0.001 ‘**’ 0.01 ‘*’ 0.05 ‘.’ 0.1 ‘ ’ 1

ggplot(data2, aes(x = variable, y = value, color = variable, fill = variable)) +

geom_violin(alpha = 0.2) +

theme_bw()

ggplot(data2, aes(x = variable, y = value, color = variable, fill = variable)) +

geom_violin(alpha = 0.2) +

geom_point(position = position_jitter(0.3)) +

theme_bw()

ggplot(data2, aes(x = variable, y = value, color = variable, fill = variable)) +

geom_boxplot(alpha = 0.2) +

geom_point(position = position_jitter(0.3)) +

theme_bw()

ggplot(data2, aes(x = variable, y = value, color = variable, fill = variable)) +

geom_violin(alpha = 0.1) +

geom_boxplot(alpha = 0.1) +

geom_point(position = position_jitter(0.3)) +

theme_bw()

ggplot() +

geom_violin(data = data2, aes(x = variable, y = value, color = variable, fill = variable), alpha = 0.1) +

geom_errorbar(data = data3, aes(x = variable, ymin=mean-sd, ymax=mean+sd), width = 0.2)

**B：**

library(ggplot2)

set.seed(100)

data <- data.frame(x = rnorm(100, 2, 1), y = rnorm(100, 1, 1))

data2 <- melt(data)

data3 <- lapply(data, function(x) get_summary_stats(data.frame(x)))

data3

# $x

# # A tibble: 1 x 13

# variable n min max median q1 q3 iqr mad mean sd se ci

# <chr> <dbl> <dbl> <dbl> <dbl> <dbl> <dbl> <dbl> <dbl> <dbl> <dbl> <dbl> <dbl>

# 1 x 100 -0.272 4.58 1.94 1.39 2.66 1.26 0.974 2.00 1.02 0.102 0.203

#

# $y

# # A tibble: 1 x 13

# variable n min max median q1 q3 iqr mad mean sd se ci

# <chr> <dbl> <dbl> <dbl> <dbl> <dbl> <dbl> <dbl> <dbl> <dbl> <dbl> <dbl> <dbl>

# 1 x 100 -1.14 3.17 0.927 0.568 1.45 0.878 0.648 1.01 0.796 0.08 0.158

cor.test(data[,1], data[,2], method = "pearson")

# Pearson's product-moment correlation

#

# data: data[, 1] and data[, 2]

# t = -1.1205, df = 98, p-value = 0.2652

# alternative hypothesis: true correlation is not equal to 0

# 95 percent confidence interval:

# -0.30221314 0.08584318

# sample estimates:

# cor

# -0.1124713

cor.test(data[,1], data[,2], method = "spearman")

# Spearman's rank correlation rho

#

# data: data[, 1] and data[, 2]

# S = 192064, p-value = 0.1297

# alternative hypothesis: true rho is not equal to 0

# sample estimates:

# rho

# -0.1524992

ggplot(data, aes(x = x, y = y)) +

geom_point() +

geom_smooth(formula = y ~ x, method = "lm") +

**C：**

if (!requireNamespace("survminer", quietly = TRUE))

install.packages("survminer")

library(survival)

library(survminer)

# data <- lung

# colnames(data)[5] <- "variable"

fit <- survfit(Surv(time, status) ~ variable, data = data)

print(fit)

# Call: survfit(formula = Surv(time, status) ~ variable, data = data)

#

# n events median 0.95LCL 0.95UCL

# variable=1 138 112 270 212 310

# variable=2 90 53 426 348 550

survdiff(Surv(time, status) ~ variable, data = data)

# survdiff(formula = Surv(time, status) ~ variable, data = data)

#

# N Observed Expected (O-E)^2/E (O-E)^2/V

# variable=1 138 112 91.6 4.55 10.3

# variable=2 90 53 73.4 5.68 10.3

#

# Chisq= 10.3 on 1 degrees of freedom, p= 0.001

fit2 <- coxph(Surv(time, status) ~ variable, data = data)

summary(fit2)

# Call:

# coxph(formula = Surv(time, status) ~ variable, data = data)

#

# n= 228, number of events= 165

#

# coef exp(coef) se(coef) z Pr(>|z|)

# variable -0.5310 0.5880 0.1672 -3.176 0.00149 **

# ---

# Signif. codes: 0 ‘***’ 0.001 ‘**’ 0.01 ‘*’ 0.05 ‘.’ 0.1 ‘ ’ 1

#

# exp(coef) exp(-coef) lower .95 upper .95

# variable 0.588 1.701 0.4237 0.816

#

# Concordance= 0.579 (se = 0.021 )

# Likelihood ratio test= 10.63 on 1 df, p=0.001

# Wald test = 10.09 on 1 df, p=0.001

# Score (logrank) test = 10.33 on 1 df, p=0.001

# plot

ggsurvplot(fit = fit, data = data, pval = T)

1. Figure 8 Related R packages:

library(tidyverse)

library(ggplot2)

library(reshape2)

library(car)

library(rstatix)

set.seed(100)

data <- data.frame(x = rnorm(100, 2, 1), y = rnorm(100, 1, 1))

data2 <- melt(data)

data3 <- lapply(data, function(x) get_summary_stats(data.frame(x)))

data3

# $x

# # A tibble: 1 x 13

# variable n min max median q1 q3 iqr mad mean sd se ci

# <chr> <dbl> <dbl> <dbl> <dbl> <dbl> <dbl> <dbl> <dbl> <dbl> <dbl> <dbl> <dbl>

# 1 x 100 -0.272 4.58 1.94 1.39 2.66 1.26 0.974 2.00 1.02 0.102 0.203

#

# $y

# # A tibble: 1 x 13

# variable n min max median q1 q3 iqr mad mean sd se ci

# <chr> <dbl> <dbl> <dbl> <dbl> <dbl> <dbl> <dbl> <dbl> <dbl> <dbl> <dbl> <dbl>

# 1 x 100 -1.14 3.17 0.927 0.568 1.45 0.878 0.648 1.01 0.796 0.08 0.158

data3 <- rbind(data3[[1]], data3[[2]])

data3[1] <- c("x", "y")

## Shapiro-Wilk normality test

lapply(data, function(x) shapiro.test(x))

# $x

#

# Shapiro-Wilk normality test

#

# data: x

# W = 0.98836, p-value = 0.535

#

#

# $y

#

# Shapiro-Wilk normality test

#

# data: x

# W = 0.98532, p-value = 0.3348

## Levene's Test

leveneTest(value~variable, data = data2)

# Levene's Test for Homogeneity of Variance (center = median)

# Df F value Pr(>F)

# group 1 4.4476 0.03621 *

# 198

# ---

# Signif. codes: 0 ‘***’ 0.001 ‘**’ 0.01 ‘*’ 0.05 ‘.’ 0.1 ‘ ’ 1

t.test(value~variable, data = data2, var.equal = T)

# Two Sample t-test

#

# data: value by variable

# t = 7.6613, df = 198, p-value = 8.012e-13

# alternative hypothesis: true difference in means is not equal to 0

# 95 percent confidence interval:

# 0.7364913 1.2470521

# sample estimates:

# mean in group x mean in group y

# 2.002913 1.011141

t.test(value~variable, data = data2, var.equal = F)

# Welch Two Sample t-test

#

# data: value by variable

# t = 7.6613, df = 186.92, p-value = 9.657e-13

# alternative hypothesis: true difference in means is not equal to 0

# 95 percent confidence interval:

# 0.7363983 1.2471452

# sample estimates:

# mean in group x mean in group y

# 2.002913 1.011141

wilcox.test(value~variable, data = data2)

# Wilcoxon rank sum test with continuity correction

#

# data: value by variable

# W = 7844, p-value = 3.711e-12

# alternative hypothesis: true location shift is not equal to 0

summary(aov(value~variable, data = data2))

# Df Sum Sq Mean Sq F value Pr(>F)

# variable 1 49.18 49.18 58.7 8.01e-13 ***

# Residuals 198 165.90 0.84

# ---

# Signif. codes: 0 ‘***’ 0.001 ‘**’ 0.01 ‘*’ 0.05 ‘.’ 0.1 ‘ ’ 1

ggplot(data2, aes(x = variable, y = value, color = variable, fill = variable)) +

geom_violin(alpha = 0.2) +

theme_bw()

ggplot(data2, aes(x = variable, y = value, color = variable, fill = variable)) +

geom_violin(alpha = 0.2) +

geom_point(position = position_jitter(0.3)) +

theme_bw()

ggplot(data2, aes(x = variable, y = value, color = variable, fill = variable)) +

geom_boxplot(alpha = 0.2) +

geom_point(position = position_jitter(0.3)) +

theme_bw()

ggplot(data2, aes(x = variable, y = value, color = variable, fill = variable)) +

geom_violin(alpha = 0.1) +

geom_boxplot(alpha = 0.1) +

geom_point(position = position_jitter(0.3)) +

theme_bw()

ggplot() +

geom_violin(data = data2, aes(x = variable, y = value, color = variable, fill = variable), alpha = 0.1) +

geom_errorbar(data = data3, aes(x = variable, ymin=mean-sd, ymax=mean+sd), width = 0.2)
